# Short-lived AUF1 p42-binding mRNAs of RANKL and BCL6 have two distinct instability elements each

**DOI:** 10.1371/journal.pone.0206823

**Published:** 2018-11-12

**Authors:** Afzal M. Dogar, Ramona Pauchard-Batschulat, Barbara Grisoni-Neupert, Larry Richman, Alexandra Paillusson, Sylvain Pradervand, Otto Hagenbüchle, Giovanna Ambrosini, Christoph D. Schmid, Philipp Bucher, Lukas C. Kühn

**Affiliations:** 1 Ecole Polytechnique Fédérale de Lausanne (EPFL), SV—Sciences de la Vie, ISREC—Swiss Institute for Experimental Cancer Research, Lausanne, Switzerland; 2 Center for Integrative Genomics (CIG), University of Lausanne, Génopode, Lausanne, Switzerland; 3 Swiss Institute of Bioinformatics (SIB), Lausanne, Switzerland; Tsinghua University School of Life Sciences, CHINA

## Abstract

Regulation of mRNA stability by RNA-protein interactions contributes significantly to quantitative aspects of gene expression. We have identified potential mRNA targets of the AU-rich element binding protein AUF1. Myc-tagged AUF1 p42 was induced in mouse NIH/3T3 cells and RNA-protein complexes isolated using anti-myc tag antibody beads. Bound mRNAs were analyzed with Affymetrix microarrays. We have identified 508 potential target mRNAs that were at least 3-fold enriched compared to control cells without myc-AUF1. 22.3% of the enriched mRNAs had an AU-rich cluster in the ARED Organism database, against 16.3% of non-enriched control mRNAs. The enrichment towards AU-rich elements was also visible by ARE*Score* with an average value of 5.2 in the enriched mRNAs versus 4.2 in the control group. Yet, numerous mRNAs were enriched without a high ARE score. The enrichment of tetrameric and pentameric sequences suggests a broad AUF1 p42-binding spectrum at short U-rich sequences flanked by A or G. Still, some enriched mRNAs were highly unstable, as those of TNFSF11 (known as RANKL), KLF10, HES1, CCNT2, SMAD6, and BCL6. We have mapped some of the instability determinants. HES1 mRNA appeared to have a coding region determinant. Detailed analysis of the RANKL and BCL6 3’UTR revealed for both that full instability required two elements, which are conserved in evolution. In RANKL mRNA both elements are AU-rich and separated by 30 bases, while in BCL6 mRNA one is AU-rich and 60 bases from a non AU-rich element that potentially forms a stem-loop structure.

## Introduction

mRNA stability plays a significant role in gene expression as it determines, together with transcription rates, mRNA steady state levels. Various *cis*-acting elements and *trans*-acting proteins can influence the fate of mRNA in the cytoplasm. Proteins that enhance or inhibit mRNA degradation may interact directly with RNA determinants or modulate mRNA decay indirectly by influencing RNA localization or translation. Furthermore, such proteins are targeted by signaling pathways. A “post-transcriptional operon” hypothesis postulates that RNA-binding proteins, analogous to transcription factors, can influence expression of many genes at once [1]. Although this hypothesis is reasonable and appealing, we are far from knowing which mRNAs are influenced by which proteins and mechanisms. To this end it is essential to analyze thoroughly the targets and binding specificities of RNA-binding proteins as they occur *in vivo*. Here we present such a study for AUF1 (also known as hnRNP D).

AUF1 is one of several proteins with a binding specificity for AU-rich elements (AREs) in 3’untranslated regions (3’UTRs) [2]. AUF1 exists in four isoforms as a result of alternative splicing of exons 2 and 7 [3]. AUF1 p45 includes 19 amino acids of exon 2 and 49 amino acids of exon 7, whereas AUF1 p37 has neither of them. p40 has only the exon 2 sequence and p42 the exon 7 sequence. AUF1 p37 and p42 bind more firmly to AREs than the other two isoforms both *in vitro* and *in vivo* [3–5]. However, this may change under conditions of kinase activation [6–8]. Based on RNA interference experiments and AUF1 binding studies, it was concluded that AUF1 is an mRNA destabilizing protein [4, 9–12]. In AUF1 knock-out mice, however, AUF1 is not required for TNF-α and IL-1β mRNA instability, except after lipo-polysaccharide injection and strong induction of proinflammatory cytokines [13]. This indicates that AUF1 is needed as a back-up to other mRNA destabilizing proteins that might be present in limited amount, like tristetraprolin (TTP) and its family members [14–16], or KSRP [17], that bind to AREs with well documented mRNA destabilizing functions. Binding to AREs may in addition be counteracted by HuR that appears to have overlapping RNA-binding specificity with mRNA stabilizing effects [11]. Overexpression of AUF1 on the other hand also stabilizes ARE-containing mRNAs [4, 18, 19] by an unknown mechanism that might relate to its oligomerization properties [5]. Two studies have defined potential AUF1 target mRNAs by microarray analysis of mRNAs co-immunoprecipitated with anti-AUF1 antibodies [11, 20]. More recently AUF1-binding sites were defined by the PAR-CLIP method [21]. In contrast to all previous studies, a consensus for AUF1-binding sites was predicted with highest frequency at U-rich sequences including G-residues.

AREs are among the best characterized mRNA destabilizing determinants which induce a rapid shortening of the poly(A)-tails prior to 3’-5’ degradation of the mRNA [22–24]. Deletion of AREs typically provokes increased mRNA levels, while grafting them into the 3’UTR of an otherwise stable mRNA can be sufficient to induce instability [25, 26]. In some pronounced cases, like GM-CSF mRNA, AREs consist of several AUUUA repeats with at least a tandem repeat of an UUAUUUA(U/A)(U/A) sequence [27, 28]. In other cases, as the mRNAs of G-CSF [29], TNF-α [30], endothelin-1 [31] or interleukin-6 (IL6) [4], the AUUUA repeats are shorter and adjacent regions contribute to rapid mRNA degradation. Although AREs were catalogued in an ARE database [32, 33], it remains impossible to predict which AREs contribute to instability. Predicted AUUUA repeats in 3’UTRs correlate in only 15 to 30 percent of cases with a short mRNA half-life [34–36]. Furthermore, 3–5% of all mRNAs in mouse ES cells [37], human T lymphocytes [35], or the human HepG2 cell line [36] are unstable with half-lives of less than 2 h, but many of them have 3’UTR sequences that do not contain classical AREs. This suggests that sequences other than AREs can induce instability.

The purpose of the present study was to define new AUF1 targets and to explore to which extent AUF1 contributes to mRNA instability. To define AUF1 targets we used RNP-co-immunoprecipitation (RNP-IP) in combination with microarrays. We took advantage of the myc-tagged AUF1 isoform p42, which binds firmly to a critical ARE of IL6 mRNA [4] and expressed it in NIH/3T3 cells prior to RNP-IP with anti-myc antibodies. We established a list of 508 potential target mRNAs that were enriched more than 3-fold in the specific RNP-IP. More than 20 of them were confirmed by quantitative RT-PCR. The list was annotated with ARE scores [38], presence in the ARE database “ARED Organism” [32, 33] and known half-lives [35–37]. It became clear that AUF1 p42 does not preferentially bind to unstable mRNAs. Therefore, it seemed essential to analyze in further detail some unstable AUF1-targets. Three 3’UTRs of particularly unstable mRNAs in our list, HES1, TNFSF11 (more commonly known as Receptor activator of NF-κB ligand (RANKL) or as osteoclast differentiation factor), and BCL6 were dissected for instability elements and protein binding sites. While HES1 mRNA revealed a coding region instability determinant, the RANKL and BCL6 UTR showed two distinct instability elements each, one of which overlapped with an AUF1 p37 binding site.

## Materials and methods

### Cell culture and stable transfections

#### Cell culture

Mouse embryonic NIH/3T3 fibroblasts (ATCC CRL-1658), human embryonic kidney (HEK) 293T cells (ATCC CRL-3216), and Phoenix ecotropic packaging cells (ATCC CRL-3214) were cultured at 37°C with 5% CO_2_ in Dulbecco’s Modified Eagle Medium (DMEM) Glutamax (Invitrogen, Life Technologies, Carlsbad, CA) supplemented with 10% heat-inactivated fetal calf serum (FCS) and Penicillin-Streptomycin-Neomycin antibiotic mixture (Invitrogen).

#### Stable transfection for microarray analysis

To induce expression of mouse AUF1 p42 fused to an amino-terminal myc-tag, two retroviral vectors, pBSwitch and pSLHGC-MYC-AUF1 p42 harboring the mifepristone-inducible GeneSwitch expression system (Invitrogen) were used as described [4]. Retrovirus was produced in Phoenix cells by standard procedures [4], filtered through 0.45 μm filters (Millipore, Billerica, MA), supplemented with 8 μg/ml polybrene and used to infect exponentially growing NIH/3T3 cells seeded 20 h before at 10% confluence. Infected cells were selected for stable integration for 6–8 days with 10 μg/ml blasticidin in the presence of 1 nM mifepristone.

#### Stable transfection of EGFP-UTR constructs

Retrovirus was generated in subconfluent Phoenix cells. Plasmid transfection was performed for 6 h in two 10 cm^2^-wells with 7.5 μg DNA using an improved calcium phosphate method protocol [39]. 40 h later, 4 ml Phoenix culture medium was filtered through 0.45 μm filters, supplemented with 2 ml fresh medium and 8 μg/ml polybrene, and used to infect three 10 cm^2^-wells of exponentially growing NIH/3T3-pBHTTA cells at 15% confluence. 48 h after infection, cells were selected with 10 μg/ml puromycin to generate three independent cell pools with stably integrated virus.

### RNP-IP and RNA isolation for microarray analysis

About 2.5 x 10^6^ logarithmically growing NIH/3T3 cells, stably transfected with pBSwitch and inducible pSLHGC-MYC-AUF1 p42 [4], were induced for myc-AUF1 expression with 1 nM mifepristone for 24 h. Cells were lysed in 750 μl CelLytic-M lysis reagent (C-2978; Sigma, St. Louis, MO), cleared by centrifugation at 10,000x*g* and the supernatant incubated at 4°C for 4 h with anti-c-myc agarose beads (A-7470; Sigma) according to the manufacturer’s protocol. Immunoprecipitates were harvested by 15 sec centrifugation at 10,000x*g* and the beads washed twice with 1 x IP buffer (I-5779; Sigma), and resuspended in 100 μl of 0.1 x IP buffer. RNA was isolated using Qiagen RNeasy minikit (Qiagen, Hilden, Germany) after DNAse I digestion according to the Qiagen manual. Control cells were not induced with mifepristone but the rest of the procedure was kept identical. A small fraction of mRNA was quantified on a NanoDrop apparatus (ND-1000, NanoDrop Technologies, Wilmington, DE) and the RNA quality analyzed using an Agilent Bioanalyzer chip (Agilent Technologies, Santa Clara, CA). All samples showed a similar pattern with a clear 18S rRNA peak, submolar amounts of 28S rRNA, some specific degradation products of rRNA, and mRNA. RNP-IPs of control cells differed only in their threefold lower RNA concentration.

### cRNA synthesis and microarray hybridization

For the analysis on Affymetrix GeneChips (Affymetrix, Santa Clara, CA) 50 ng of immunoprecipitated RNA or of a total RNA control sample were processed with the two-cycle target labelling protocol (Technical Manual; Gene Expression Analysis 701021 Rv.3 of Affymetrix) to produce biotinylated cRNA target. 11 μg of this amplified material of each sample were fragmented and hybridized to GeneChip Mouse 430 2.0 arrays. Washing and staining was performed using the EuGE-WS2v5 protocol. Scanning was done on the Affymetrix GeneChip Scanner 7G. Expression signals were normalized by robust multiarray average (RMA) [40]. Differentially hybridizing features were identified using Limma [41] and adjusted for multiple testing with the Benjamini and Hochberg’s method. Alternatively, Z scores and Z ratios were calculated according to [42]. Microarray data are available at NCBI Gene Expression Omnibus at accession number GSE64761.

### Annotation of microarray data

Besides adding the gene name of NCBI and gene ID of Entrez, we calculated the number of AREs with ARE*Score* [38]. All UTRs with a score of 0 or a 3’UTR length <1000 bases were manually inspected for their polyadenylation signal. In case of doubts, the NCBI database was searched for a better 3’end prediction to calculate the ARE*Score*. In case of alternative 3’UTRs, always the one with the highest score is reported. For qualitative comparison we screened the ARED Organism database [33] with the enriched mRNA list (S7 Table) and a control group of 800 non-enriched mRNAs. For known mRNA stability data, we consulted studies in mouse ES cells [37], human T lymphocytes [35], and human HepG2 cells [36].

### Plasmid constructs

#### Tet-off system

The retroviral plasmid vectors pBHTTA and pZTCTHI were constructed as described in S1 Methods. A stably transfected NIH/3T3-pBHTTA cell line expressing the tetracycline-sensitive trans-activator [43, 44] constitutively was selected with 150 μg/ml hygromycin and used to test all pZPCTHI-constructs.

#### Analysis of 3’UTRs

3’UTR-constructs were made in the EGFP-reporter plasmid pZPCTHI. 3’UTRs of mouse and human RANKL, human KLF10, IL6, HES1, SMAD6 and BCL6 were PCR-amplified from cDNA-containing plasmids with primers shown in S1 Table and inserted as *Eco*RI-*Not*I fragment into corresponding sites of pZPCTHI. For pHES1-CDS-UTR, the human HES1 coding region with its 3’UTR was PCR-amplified and subcloned as a *Bgl*II-*Not*I fragment in frame behind EGFP into pZPCTHI_no_stop.

#### Deletions and scanning mutations in mouse RANKL 3’UTR

They were made by PCR amplification with primers shown in S2 Table. Large deletion constructs pRANKL.2, 8, 11 and 12 and short deletion construct pRANKL.22 were amplified and cloned as *Eco*RI-*Not*I fragments into pZPCTHI. Constructs pRANKL.3 to 5 were amplified and cloned as *Bgl*II-*Not*I fragments into pRANKL.2. Constructs pRANKL.6 and 7 were amplified and cloned as *Eco*RI-*Bgl*II fragments into pRANKL.8 Constructs pRANKL.9 and 10 were amplified and cloned as *Eco*RI-*Bgl*II fragments into pRANKL.5 and 4 respectively. Constructs pRANKL.13 and 14 were amplified from pRANKL.9 and cloned as *Eco*RI-*Not*I fragments into pRANKL.9. Short 50- to 70-base deletion constructs pRANKL.15 to pRANKL.21 were made by generating first two overlapping PCR-fragments that were then mixed and reamplified with external PCR primers for pRANKL.11 and cloned as *Eco*RI-*Not*I fragment into pZPCTHI. The deletion was replaced by the *Bgl*II restriction site AGATCT. The 15-base scanning mutations pRANKL.23 to pRANKL.30 were generated in the same way except that the deleted 15-base sequence was replaced by the 15-base linker sequence AGCAACGCGTAGCTC harbouring a unique *Mlu*I site.

#### Deletions and scanning mutations in human BCL6 3’UTR

They were made by PCR amplification with primers shown in S3 Table. Large deletion constructs pBCL6.1 to pBCL6.10 and short deletion constructs pBCL6.13 and pBCL6.20 were amplified and cloned as *Eco*RI-*Not*I fragments into pZPCTHI. For constructs pBCL6.11 and pBCL6.12, PCR-amplified fragments were inserted as *Eco*RI-*Bgl*II fragments into pBCL6.8 and pBCL6.5 respectively. Short 50-base deletion constructs pBCL6.14 to pBCL6.19 were made by generating first two overlapping PCR-fragments that were then mixed and reamplified with external PCR primers for pBCL6.7 and cloned as *Eco*RI-*Not*I fragment into pZPCTHI. The 50-base deletions were replaced by the *Bgl*II restriction site AGATCT. The 15-base scanning mutations pBCL6.21 to pBCL6.32 were generated in the same way except that the deleted 15-base sequence was replaced by the 15-base linker sequence AGCAACGCGTAGCTC harbouring a unique *Mlu*I site.

#### 3’UTR fragments of mouse RANKL and human BCL6 mRNA for RNP-IP

Plasmids with long or medium 3’UTR inserts were used in transient expression assays to study RNA-protein interactions. Some of these plasmids were identical to those used for EGFP-3’UTR mRNA half-life measurements and are described above. Additional fragments of mouse RANKL and human BCL6 3’UTR were amplified with appropriate primers shown in S4 and S5 Tables and subcloned in corresponding restriction sites of either pZPCTHI or constructs pRANKL.6 and pBCL6.2. Primers for pBCL6.16i, in a region of difficulties for PCR, were annealed and directly ligated to pZPCTHI.

#### Plasmids for transient protein expression and RNP-IP

The interaction of RNA-binding proteins with potential RNA targets was analyzed by transient protein expression with the GeneSwitch expression system (Invitrogen). Myc-tagged mouse AUF p37 was expressed from pSLHGC-MYC-AUF1 p37 [4]. To express myc-tagged HuR, the coding sequence of human HuR (NM_001419.2) was amplified using the forward primer CCGGAATTCCGGATGTCTAATGGTTATGAAGACCACAT and reverse primer ATAAGAATGCGGCCGCTAAACTATTTATTTGTGGGACTTGTTGGTTT. The PCR fragment was digested with *Eco*RI and *Not*I and ligated into corresponding sites of pSLHGC- MYC-AUF1 p37 [4]. A plasmid to express His-tagged KSRP expression plasmid was kindly provided by Helmut Holtmann (Hannover, Germany) [45]. All plasmid inserts were verified by sequencing.

### mRNA half-life measurements by real-time PCR

#### Endogenous mRNAs

Cytoplasmic RNA was isolated with the RNeasy mini kit (Qiagen) from cell cultures at 70% confluence. For RNA half-life measurements, actinomycin D (Sigma) was added at 6 μg/ml for 0, 30, 60, 120, or 180 min prior to RNA extraction. RNA was extracted and reverse-transcribed into cDNA by standard methods. Real-time PCR measurements were carried out with the Roche Lightcycler (Roche Diagnostics, Rotkreuz, Switzerland) in conjunction with Master Sybr green I according to the manufacturer’s recommendations. Primer sets used are shown in S6 Table. Measurements were normalized to mouse acidic ribosomal protein P0 (mARP0) mRNA that were considered to be stable within the time-frame tested [46].

#### mRNA degradation of EGFP-3’UTR constructs

For each pZPCTHI-based construct, three independent stably transfected NIH/3T3-pBHTTA cell pools were analyzed at 60 to 80% confluence. Transcription of pZPCTHI-constructs was blocked by inhibiting the tetracycline-sensitive trans-activator with 40 ng/ml doxycycline. RNA was isolated 8, 68 and 128 min after doxycycline addition, and the 8-min time-point was considered as the start of EGFP mRNA decay. Cytoplasmic RNA was isolated using the RNeasy mini kit (Qiagen) or Nucleospin RNA II kit (Macherey-Nagel, Düren, Germany).

#### cDNA synthesis

0.3 to 1 μg cytoplasmic RNA and 300ng random primer (MWG Biotech, Ebersberg Germany) were denatured 10 min at 65°C and cooled on ice for 5 min. After addition of first strand buffer (1x final concentration) (Invitrogen), DTT (10 mM final), dNTPs (0.5 mM final for each), 5 U RNaseOUT (Invitrogen) and 1 μl (200 U) M-MLV reverse transcriptase (Invitrogen), the reaction in 20 μl total volume was allowed to proceed for 1.5 h at 37°C. The enzyme was inactivated 5 min at 95°C, and 480 μl 5 mM Tris-HCl, pH 8.0, with 50 ng/ml yeast tRNA were added to the reaction.

#### Quantitative real-time PCR (qRT-PCR)

We used the LightCycler FastStart DNA Master^PLUS^ HybProbe kit (Roche Diagnostics). 5 μl diluted cDNA were mixed with 5 μl of 2x reaction master mix in 20 μl LightCycler glass capillaries (Roche) to obtain a final 1x-reaction buffer with 5 mM MgCl_2_, 200 μM dNTPs (dUTP instead of dTTP) and Faststart Taq DNA polymerase, supplemented with 0.1 U Uracil-DNA-glycosylase (Bioline, Taunton, MA), forward and reverse, and specific probe primers. The qRT-PCR conditions for the Roche Lightcycler were 2 min at 50°C, 15 min at 95°C, 37 cycles with 10 sec at 95°C and 1 min at 60°C (single acquisition), and finally 6 min at 37°C. Standard curves for the EGFP, GAPDH or mARP0 were prepared using a dilution series of total cDNA from stably transfected cells. Afterwards, two samples of this dilution series were included in every Lightcycler run as a standard.

#### Primers and probes

EGFP primers were GFPI-F (ACTTCATCTAGGCACGGCAGAA) and GFPI-R (CGTCCAGCTCGACCAGGAT) used at a final concentration of 500 nM and 700 nM, respectively, in combination with 200 nM GFPI-probe (FAM- TCCACTCCCAATTGCCACCATGG-TAMRA). Endogenous human GAPDH or mouse ARP0 mRNA were used to normalize the amplification signal. GAPDH-primers were GAPDH-F (GAAGGTGAAGGTCGGAGTC) and GAPDH-R (GAAGATGGTGATGGGATTTC) at 300nM and GAPDH-probe (FAM-CAAGCTTCCCGTTCTCAGCC-TAMRA) at 200 nM. Mouse ARP0 primers were mARP0-F (CTTTGGGCATCACCACGAA) and mARP0-R (GCTGGCTCCCACCTTGTCT) at 300 nM, and mARP0-probe (FAM-ATCAGCTGCACATCACTCAGAATTTCAATGGT-TAMRA) at 200 nM. Measurements were performed with a GeneAmp 5700 sequence detection system (Applied Biosystems, Foster City, CA).

#### Calculation of mRNA half-life

Half-lives were calculated from three to six independent experiments by plotting on a semi-logarithmic graph in Windows Excel software the percent RNA left at time-points 0, 1, and 2 h after transcription inhibition. Time-point zero was considered as 100%. The decay-slope was calculated by linear regression of all experimental points from different cell pools. The half-life derived from the slope was expressed in min ± deviation of the slope within 95% confidence. Alternatively, decay slopes were calculated separately for each cell pool and final results expressed as the average ± SD. Usually values obtained by the two methods were very close.

### Transient co-transfection of HEK 293T cells with Lipofectamine

HEK 293T cells were plated on 12-well plates, such as to reach 80–90% confluency at the time of transfection. For each well, a total of 1.6 μg plasmid DNA in 50 μl of Opti-MEM I (Gibco) was prepared. For HuR and AUF1 p37, this mix contained 30 ng of the following vectors: appropriate pSLHGC construct, pBSwitch, pBHTTA and appropriate pZPCTHI construct, together with 1.48 μg pGEM-7Zf(-) as inert carrier. For KSRP, we used 30 ng of a plasmid expressing His-KSRP, pBHTTA and appropriate pZPCTHI vector, together with 1.51 μg pGEM-7Zf(-). The quantities were determined such as to obtain a good transient expression, but low enough to permit a full RNP-IP. For each transfection, 3.2 μl of Lipofectamine 2000 (Invitrogen) were mixed with 50 μl Opti-MEM I, incubated 5 min at room temperature and then added to the DNA mixes. The DNA/Lipofectamine mixes were incubated for 20 min at room temperature and then added drop-wise to the wells. For AUF1 p37 and HuR expression only, 1 ml of medium with 4 nM mifepristone (Invitrogen) was added after 5 h and incubated for further 16 h.

### Quantitative RNP-IP

**Fig 1:**
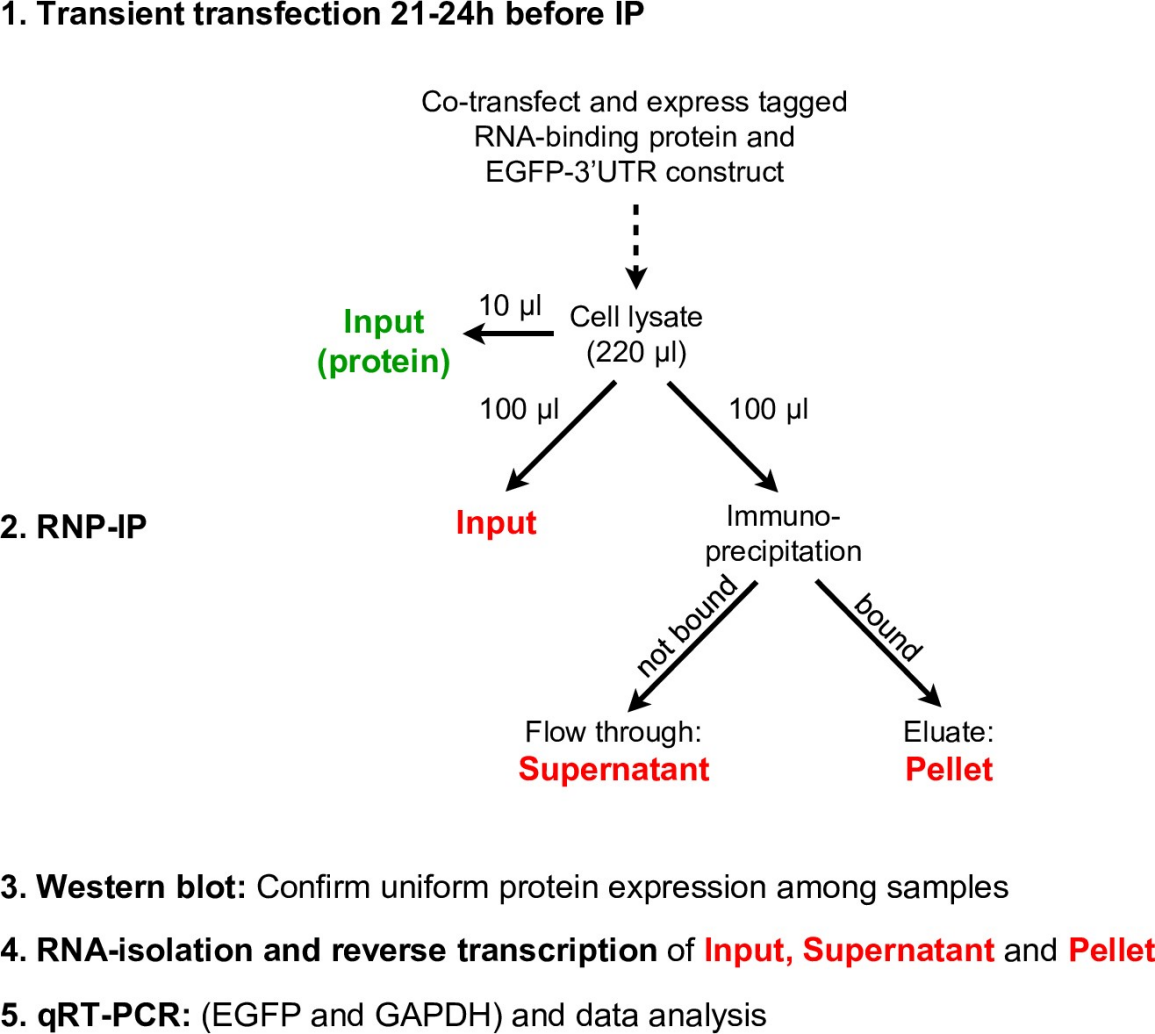
Outline of a co-immunoprecipitation experiment and analysis. Tagged ARE binding proteins as well as an EGFP reporter mRNA containing 3’UTR fragments of interest were transiently co-expressed in HEK 293T cells.

The flow-sheet (Fig 1) was carried out with transiently transfected HEK 293T cells expressing a myc- or His-tagged ARE-binding protein and an EGFP reporter mRNA with a 3’UTR of interest. Cells were washed with 1 ml ice-cold PBS and lysed in 220 μl ice-cold TD lysis buffer (150 mM NaCl, 1% Triton X-100, 50 mM Tris-HCl pH 8.0, 0.5% sodium deoxycholate) containing Complete Mini protease inhibitor (Roche; 1 tablet/7 ml buffer) and ANTI-RNase ribonuclease inhibitor (Ambion/Life Technologies; 1.5 μl/ml lysis buffer). The lysate was immediately transferred to a microfuge tube, kept on ice for 15 min and centrifuged for 15 min at 13000 rpm. 100 μl supernatant were mixed with 300 μl of RA1 buffer of NucleoSpin RNA II kit (Macherey-Nagel) containing 1% β-mercaptoethanol and stored as “Input” fraction at -20°C. 10 μl were mixed with NuPAGE 4x LDS Sample Buffer (NP0008, Novex/Life Technologies) and used for immunoblotting. 100 μl lysate supernatant were mixed with 10 μl of magnetic μMACS anti-c-myc or anti-His microbeads (130-091-123 or 130-091-285, Miltenyi Biotec, Bergisch-Gladbach, Germany) and incubated at 4°C for 60–90 min, slowly rotating on a wheel. One μ column (130-042-701, Miltenyi Biotec) per reaction was placed in the magnetic field of the μMACS Separator (130-042-602, Miltenyi Biotec) attached to a MACS MultiStand (130-042-303, Miltenyi Biotec) and washed once with 200 μl TD lysis buffer. The IP reaction was loaded on top of the column matrix, and the flow-through dripping out of the column was collected as “Supernatant” fraction. The column was washed three times with 200 μl of TD lysis buffer and once with 200 μl of Low-Salt wash buffer (20 mM Tris-HCl pH 7.5). Afterwards, 20 μl of elution buffer (50 mM Tris-HCl pH 6.8, 50 mM DTT, 1% SDS, 1 mM EDTA, 0.005% bromophenol blue, 10% glycerol) pre-heated to 98°C was applied directly on top of the column matrix and incubated for 5min. Then, another 55 μl pre-heated elution buffer was applied on top of the column and the flow-through of about 50 μl was collected in a microfuge tube and kept as “Pellet” fraction. Both the “Supernatant” and “Pellet” fractions were also mixed with 300 μl of RA1 buffer and stored at -20°C. RNA was isolated from all three fractions, reverse transcribed and analyzed by qRT-PCR.

To determine, whether a 3’UTR was bound to a protein, the enrichment of EGFP mRNA in the “Pellet” was compared to that of endogenous GAPDH mRNA. For each IP the sum of mRNA in the “Supernatant” and “Pellet” was designated as the “Total”. The recovery of “Total” was usually between 50 and 80% of the “Input”. Next, the “Pellet” was divided by the “Total” to calculate the percent of mRNA bound by a given protein. To determine the relative enrichment, the percentage obtained for EGFP mRNA was then divided by the percentage for GAPDH mRNA. The significance of the enrichment data was determined with the two-tailed homoscedastic t-test using the RNP-IP with pZPCTHI as the reference.

### Western blots

Gel electrophoresis and western blots were done by standard procedures. The following antibodies were used: anti-c-myc-HRP (R951-25, Novex), anti-His-HRP (130-092-785, Miltenyi Biotec), mouse anti-α-tubulin (T9026, Sigma), goat anti-mouse-HRP (sc-2005, Santa Cruz, Dallas TX, USA), goat anti-rabbit HRP (sc-2004, Santa Cruz), rabbit anti-human AUF1 (07–260, Upstate/Merck Millipore, Billerica, MA), mouse anti-human HuR (sc-5261, Santa Cruz).

### Statistical analysis

Spearman correlation coefficients and corresponding P-values were computed with the R function *cor*.*test*. ARE scores were computed with the program ARE*Score* [38]. Match scores to Gorospe’s signatures were computed with the COVELS program from the COVE software package [47] using the SCGF kindly provided by authors of the signature. Optimal local sequence alignments between selected 3’UTRs and/or parts of it were computed with the program PRSS3 (version 3.4t26) from the FASTA package [48]. Statistical significance (E-value) was computed by shuffling the target sequence 1000 times in windows of 10 bp.

## Results

### mRNA sequences enriched by RNP-IP with myc-AUF1 p42

**Fig 2:**
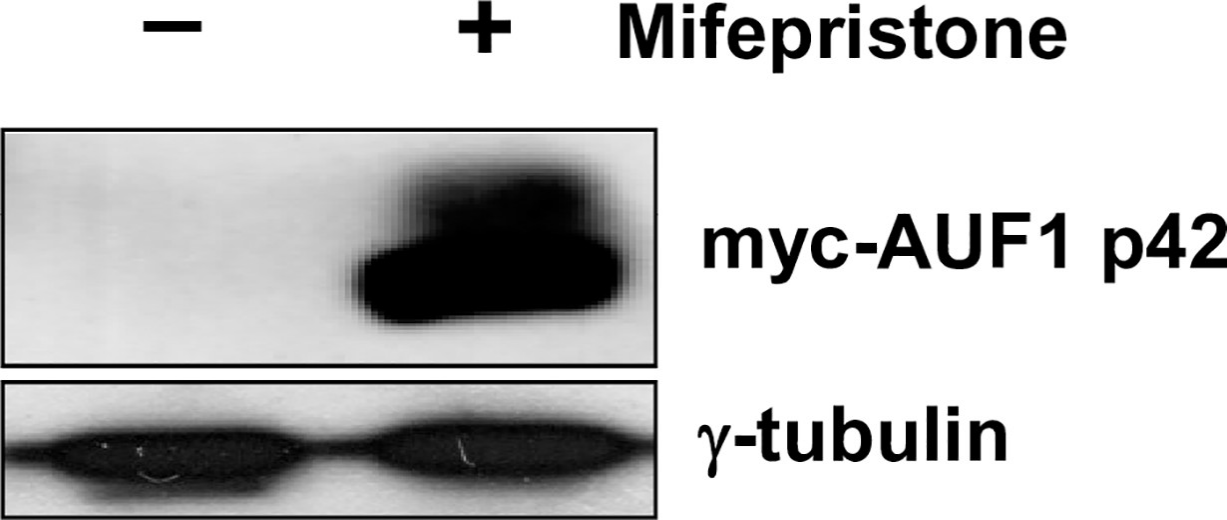
Expression of myc-AUF1 p42 in mouse 3T3 cells. Mouse 3T3 cells were stably transfected with the mifepristone-inducible vector pSLHGCMYC-AUF1 p42 [4] to express the myc-tagged AUF1 p42 isoform. The Western blot of cytoplasmic cell extracts prior (-) and after (+) 1 nM mifepristone induction was probed with anti-myc tag monoclonal antibody and anti γ-tubulin antibody.

To identify new AUF1 target mRNAs, we expressed myc-tagged AUF1 p42 that binds firmly to a specific ARE in the IL6 3’UTR and for which cytoplasmic RNA-AUF1 complexes can be readily enriched by RNP-IP with anti-myc tag antibody beads [4]. We expressed the myc-tagged AUF1 p42 in mouse NIH/3T3 cells using retroviral vectors with the mifepristone-inducible GeneSwitch system (Invitrogen) [4]. These vectors show no background expression prior to induction (Fig 2). After addition of 1 nM mifepristone, myc-AUF1 was strongly induced (Fig 2) with levels up to 10-fold compared to endogenous AUF1 [49]. We then compared mRNAs in RNP-IPs from cell extracts with and without myc-AUF1 p42 by hybridization of fragmented cRNA on GeneChip Mouse 430 2.0 arrays. Independently of myc-AUF1 p42 induction and in addition to specifically enriched mRNAs, anti-c-myc agarose beads adsorbed non-selectively some background material with an mRNA composition very similar to total control mRNA. Rather than being a problem, this background was convenient to normalize systematic differences in the average intensity of hybridization due to sample concentration or other factors.

The microarray analysis was performed with two independent RNP-IPs of myc-AUF1 p42-expressing cells, two RNP-IPs from the same cells without AUF1 induction and once with total mRNA. We made a list of all mRNAs that showed a 3-fold enrichment with an adjusted p-value <0.5 on anti-c-myc beads after AUF1 p42 induction (2 samples) compared to control beads without AUF1 p42 induction and total mRNA (2 + 1 samples). We further removed from the list all redundant sequences, 2 outliers (>70-fold enriched) and all sequences with neither a predicted function nor a human homolog. The remaining list comprises a total of 508 mRNAs (Tables 1 and S7). This corresponds to 3.3% of expressed mRNA sequences in NIH/3T3 cells as judged by the 15100±1520 presence/absence calls of the array hybridization signals. 11 of them were loci just predicted on the basis of open reading frames in mouse and human. Our experiment was validated by the presence of IL6 mRNA, for which we have previously shown that it is enriched at least 4-fold by RNP-IP of myc-AUF1 p42 [4]. Its enrichment in the present microarray analysis was 5.8-fold, confirming that the 3’UTR of IL6 mRNA binds firmly to AUF1 42.

**Table 1:** mRNAs of mouse NIH/3T3 cells enriched by RNP-IP with myc-AUF1 p42.

Gene Symbol	Entrez Gene ID	Gene Description	Refseq RNA Accession	n-Fold Enrich-ment	ARE Score	3'AUUUA Count	3'UTR Length	ARED Cluster	mRNA Half-life (h)
Tnfsf11	21943	tumor necrosis factor superfamily, member 11 (RANKL)	NM_011613	26.5	7.8	6	1138	Cluster 5	1.5(a)
Mbnl3	171170	muscleblind-like 3 (Drosophila)	NM_134163	26.1	19.9	16	7513	no ARE	stable
Dck	13178	deoxycytidine kinase	NM_007832	21.9	7.5	6	2007	no ARE	stable
Dus4l	71916	dihydrouridine synthase 4-like (S. cerevisiae)	NM_028002	19.6	0	0	621	no ARE	stable
Tmed2	56334	transmembrane emp24 domain trafficking protein 2	NM_019770	19.6	7.0	5	1352	no ARE	stable
Sap18	20220	Sin3-associated polypeptide 18	NM_009119	18.1	6.7	4	2916	Cluster 5	stable
Pcsk6	18553	proprotein convertase subtilisin/kexin type 6	NM_011048	16.9	0	0	1358	N/A	stable
Bicc1	83675	Bicaudal C homolog 1 (Drosophila)	XM_006514341	16.8	12.6	9	2541	N/A	stable
Cisd1	52637	CDGSH iron sulfur domain 1	NM_134007	16.7	1	1	543	no ARE	stable
Cct4	12464	chaperonin subunit 4 (delta)	NM_009837	15.7	1.3	1	185	no ARE	stable
Car13	71934	carbonic anhydrase 13	NM_024495	15.6	4.1	2	1428	no ARE	stable
Rmnd5a	68477	required for meiotic nuclear division 5 hom. A (S. cerev.)	NM_024288	15.4	14.2	11	4485	no ARE	stable
Maf	17132	avian musculoaponeurotic fibrosarcoma (v-maf) homolog	NM_001025577	15.3	4.9	4	2452	no ARE	stable
Tnfrsf21	94185	tumor necrosis factor receptor superfamily, member 21	NM_178589	13.6	2.6	2	1176	no ARE	stable
Cnot7	18983	CCR4-NOT transcription complex, subunit 7	NM_011135	13.5	6.2	5	1449	no ARE	stable
Man2a2	140481	mannosidase 2, alpha 2	NM_172903	12.9	1.3	1	2585	no ARE	stable
Klf10	21847	Kruppel-like factor 10	NM_013692	12.9	4.9	4	1499	no ARE	2.0(a)
Lims1	110829	LIM and senescent cell antigen-like domains 1	NM_026148	12.6	5.2	4	3123	no ARE	stable
Slc16a1	20501	solute carrier family 16, member 1	NM_009196	11.9	13.4	11	2740	Cluster 5	stable
Gatad1	67210	GATA zinc finger domain containing 1	NM_026033	11.4	5.2	4	1427	Cluster 5	stable
Tmem62	96957	transmembrane protein 62	NM_175285	11.2	0	0	717	no ARE	stable
Lpgat1	226856	lysophosphatidylglycerol acyltransferase 1	NM_172266	11.2	16.7	14	5726	no ARE	stable
Dnajb4	67035	DnaJ (Hsp40) homolog, subfamily B, member 4	NM_025926	11.1	5.2	4	1276	Cluster 5	stable
Cmbl	69574	carboxymethylenebutenolidase homolog (Pseudomonas)	NM_181588	11.1	0	0	284	no ARE	stable
Mmd	67468	monocyte to macrophage differentiation-associated	NM_026178	11.0	2.6	2	1680	Cluster 5	stable
Atp6v1a	11964	ATPase, H+ transporting, lysosomal V1 subunit A	NM_007508	11.0	7.8	6	2020	no ARE	stable
Serpinb9	20706	serine (or cysteine) peptidase inhibitor, clade B, memb. 9	NM_009256	10.9	6.2	5	2107	no ARE	stable
Rassf6	73246	Ras association (RalGDS/AF-6) domain family 6	NM_028478	10.6	1.3	1	768	no ARE	stable
Tnfaip6	21930	tumor necrosis factor alpha induced protein 6	NM_009398	10.6	8.4	6	710	no ARE	1.8(a)
Erbb3	13867	v-erb-b2 erythrobl. leukemia viral oncogene hom. 3 (avian)	NM_010153	10.6	4.9	4	1830	no ARE	stable

The list comprises the 30 most enriched mRNAs. Complete data are reported in S7 Table. n-Fold enrichment refers to microarray data comparing RNP-IPs from cells with myc-AUF1 p42 to RNP-IPs from cells without. The ARE score, number of AUUUA elements in the 3'UTR, and 3'UTR length were determined by the program ARE*Score* [38]. The ARED cluster refers to the presence in the ARED Organism database [32, 33]. Known mRNA half-lives in mouse ES cells (a) are given in numbers below 3h or as "stable" above 3h [37]. N/A, not available. Microarray data are available at NCBI GEO with ID GSE64761.

In order to confirm the enrichment revealed by the microarray data, a selection of 21 mRNAs of particular interest and with functions in signaling and transcription were submitted to quantitative RT-PCR in 2 independent RNP-IPs of cells expressing myc-AUF1 p42 or non-induced cells (Table 2). 18 of the selected mRNAs showed a strong enrichment, 1 was only 2-fold enriched and for 2, the amount in the control pellet without myc-AUF1 p42 was too low to be measured by RT-PCR (marked with “nd”). It confirms that most mRNAs of Tables 1 and S7 are interacting with AUF1 p42, and that microarray data are reliable in this respect.

**Table 2:** List of selected mRNAs enriched by RNP-IP of myc-AUF1 p42.

Gene symbol	Gene full name	Enriched on array	Enriched RT-PCR	mRNA half-life^a^	mRNA half-life^c^	EGFP-UTR half-life^d^	Number of 3'-AUUUA	ARED cluster
Tnfsf11	Tumor necrosis factor family member 11 (RANKL)	26.5	89	30	90	63.8±7.6 (5)	6	5
Bicc1	Bicaudal C homolog 1 (Drosophila)	16.8	nd	315	780	nd	9	N/A
Maf	v-maf AS42 oncogene homolog (avian)	15.3	5	90	400	nd	4	no
Tnfrsf21	Tumor necrosis factor receptor superfamily member 21	13.6	14	132	460	nd	2	no
Cnot7	CCR4-NOT transcription complex, subunit 7	13.5	11	198	265	nd	5	no
Klf10	Kruppel-like factor 10	12.9	43	45	120	77.0±11.2 (2)	4	no
Lims1	LIM and senescent cell antigen-like domains 1	12.6	2	230	400	nd	4	no
Tnfaip6	Tumor necrosis factor alpha induced protein 6	10.6	43	120	105	nd	6	no
Erbb3	v-erb-b2 erythroblastic leukemia oncogene homolog 3	10.6	49	125	340	nd	4	no
Ptp4a1	Protein tyrosine phosphatase 4a1	9.0	22	130	215	nd	15	5
Sin3a	SIN3 homolog A, transcriptional regulator (yeast)	9.0	4	90	205	nd	2	no
Ptpn2	Protein tyrosine phosphatase, non-receptor type 2	8.8	6	131	260	nd	12	no
Smad5	Mothers against decapentaplegic homolog 5 (Drosophila)	8.7	5	128	235	nd	6	no
Bmpr1a	Bone morphogenetic protein receptor, type 1A	6.3	15	134	260	nd	7	no
Il6	Interleukin 6	5.8	14	27^b^	nd	25.1±5.2 (5)	5	5
Hes1	Hairy and enhancer of split 1 (Drosophila)	5.7	47	20	70	87.4±14.4 (6)	0	no
Fzd4	Frizzled homolog 4	5.4	16	52	nd	nd	3	no
Ccnt2	Cyclin T2	5.0	nd	52	185	nd	5	no
Smad6	Mothers against decapentaplegic homolog 6 (Drosophila)	4.4	14	20	115	111.1±20.5(5)	2	5
Cdc42	Cell division cycle 42 homolog (S. cerevisiae)	4.3	7	418	460	nd	1	no
Bcl6	B-cell leukemia/lymphoma 6	3.8	16	35	80	43.7±7.8 (5)	1	no

nd, not determined; N/A, not available.

^a^ Measured in mouse 3T3 cells (this study; results in S1 Fig).

^b^ Measured in mouse 3T3 cells by Paschoud et al. [4].

^c^ Measured in mouse ES cells by Sharova et al. [37].

^d^ EGFP-human 3'UTR construct expressed in mouse NIH/3T3-BHTTA cells and mRNA half-life tested (this study); in parenthesis, number of independant experiments.

### Co-immunoprecipitation with AUF1 p42 did not enrich for unstable mRNAs

We next wanted to know whether RNP-IP of AUF1 p42 selects for unstable mRNAs. Extensive data for mRNA stability are not available for mouse NIH/3T3 cells, but exist for mouse ES cells [37], and partially for human HepG2 cells [36] and human lymphocytes [35]. We therefore annotated our list of AUF1 p42-bound mRNAs (Tables 1 and S7) with the half-life of these other studies. Of 508 mRNAs, 493 were in the list for ES cells [37] and 141 in the work on HepG2 cells [36]. 23 had a reported half-life of ≤2h in at least one study and 15 scored ≤2h in one study but were reported more stable in another one. Additional 35 mRNAs had reported half-lives between 2 and 3h. Provided mRNA half-lives are similar in different cell types, about 7.5% of the mRNAs in our AUF1 target list would be predicted unstable with a half-life ≤2h, a value close to the random frequency of unstable mRNAs in HepG2 cells [36]. We tentatively concluded that myc-AUF1 p42 does not preferentially bind to unstable mRNAs. We then tested experimentally whether some of our enriched mRNAs coding for proteins in transcription and signal transduction were unstable. 21 mRNAs were analyzed by RT-PCR for their decay after actinomycin D treatment (Table 2; S1 Fig). The half-life of 8 mRNAs (38%) was ≤60 min and of 3 additional ones ≤2 h, with an average for the entire set of about 2 h. Although this set showed a statistically significant enrichment for unstable mRNAs, we believe, in view of the overall comparison with half-lives in ES cells [37], that the selected mRNAs showed a bias towards instability that was probably introduced by the educated choice of mRNAs coding for a specific category of proteins. Previous studies in HepG2 cells reported a frequency of 13.1% unstable mRNAs among transcription factors [36]. Nonetheless, there was a strong correlation between our half-life measurements in NIH/3T3 cells and the ones reported in ES cells (p<0.001; Table 2). 6 of our 9 short-lived mRNAs had a half-life ≤2 h in both studies (Tnfsf11 = RANKL, Klf10, Tnfaip6, Hes1, Smad6 and Bcl6) while 2 additional ones (IL6 and Fzd4) were not present in the ES cell study [37]. It supports the idea that mRNA instability is conserved in different cells types and that it is legitimate to annotate our list with data from ES-cells.

### Presence of enriched mRNAs in ARED database and ARE*Score* values

To be enriched by RNP-IP of AUF1 p42, mRNAs are expected to have binding sites for AUF1, which may consist of a specific sequence element. Alternatively, interactions might also occur at multiple sites or indirectly via other proteins. Since previous studies have shown that classical AREs can bind AUF1, we searched the ARED Organism database [32, 33]. Of the 508 enriched mouse mRNAs in our list, 467 were recognized by the program and 104 (22.3%) identified with an ARE cluster (S7 Table). In a control set of 800 non-enriched mRNAs, 130 (16.3%) were present in the ARED Organism database. We conclude that RNP-IP of myc-AUF1 p42 leads to an enrichment of about 1.4-fold for ARE-containing mRNAs. If we restrict the comparison to the 11 sequences with short half-lives of <120 min (Table 2), 4 were in the ARED database.

**Fig 3:**
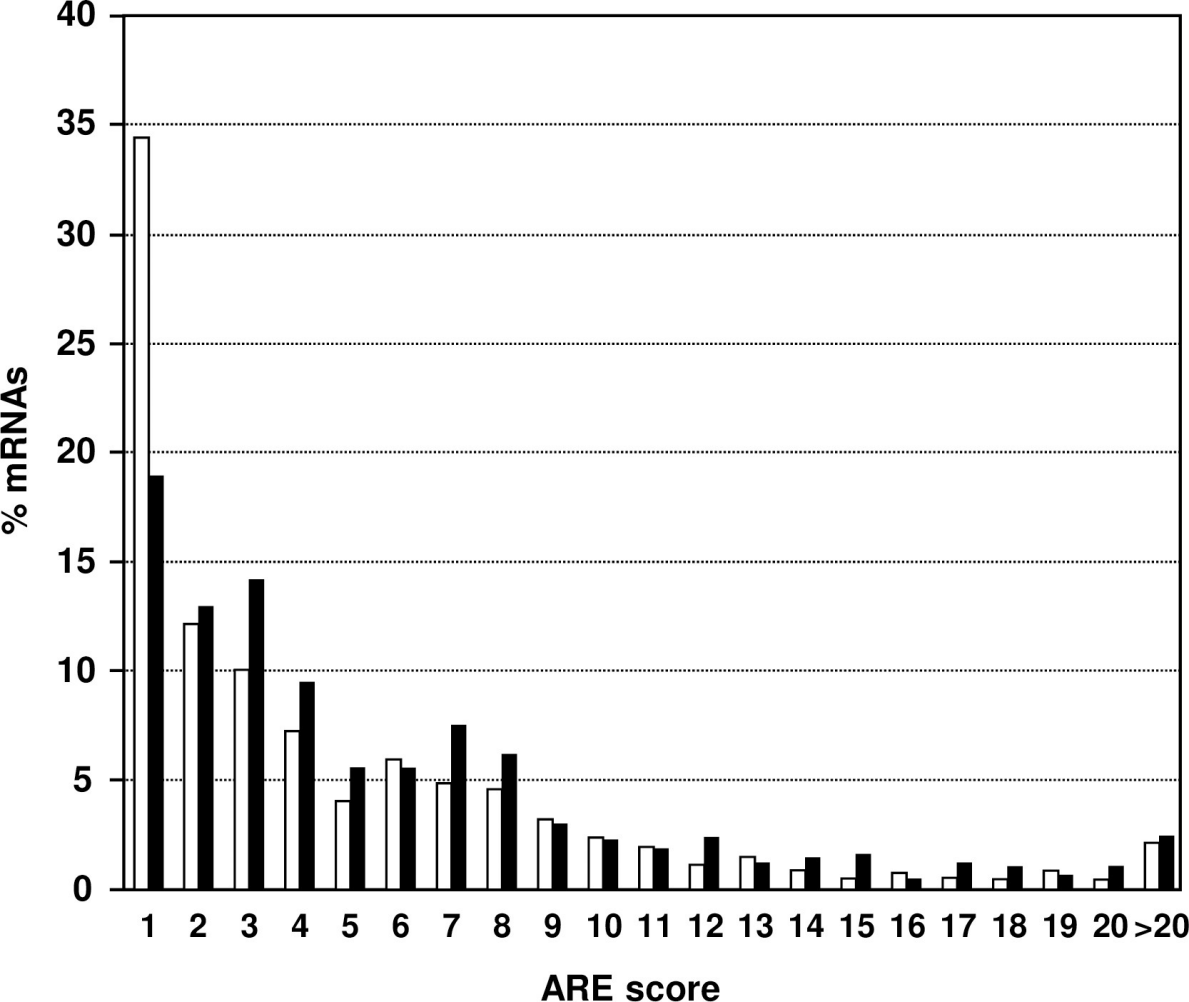
Assessment of ARE*Score*. The 508 AUF1 p42 bound mouse mRNAs that were more than 3-fold enriched on microarrays (black bars) were analyzed by the algorithm of ARE*Score* [38] and compared to 800 non-enriched mRNAs (white bars). Frequencies of ARE scores between 1 to 20 and >20 were plotted.

AU-rich regions in 3’UTR can also be analyzed with the program ARE*Score* that calculates scores based on the number of AUUUA elements and their environment [38]. We submitted the 508 enriched 3’UTR sequences and 800 3’UTR sequences of non-enriched mRNAs to this program. The results are reported in Tables 1 and S7 with the RefSeq numbers used for the analysis. We found an average score of 5.2 for the enriched sequences and 4.3 for the non-enriched controls. The average 3’UTR length was close to 1700 b in both sets. The score distribution was significantly different in the two sets. The control list comprised 34.4% of sequences with scores of 0 and 1, similar to total mouse mRNAs [38], while only 18.9% had such low scores in the list of AUF1 p42 enriched mRNAs (Fig 3). In addition the enriched list showed higher number of sequences with scores of 3 to 8 (48.2%) versus the control list (36.8%). Again, we conclude on a moderate selection towards AUUUA sequences by AUF1 p42.

We further analyzed by statistical methods whether enrichment on myc-AUF1 p42 correlates with a specific sequence motifs (Table 3). For this, the list of 508 enriched mRNAs was combined with a control list of 800 non-enriched mRNAs. We used the Spearman’s Rank correlation coefficient statistics to estimate the association of AUF1 p42 enrichment with various sequence-derived predictor variables: percentage of individual nucleotides, combined A+U and G+U content, the number of various oligonucleotides, sequence length, presence or absence of the mRNA in the ARED database, ARE motif score and best match score to the AUF1 binding signature published by Gorospe and coworkers [50]. In addition, we analyzed the 5 to 6 most highly enriched trimers, tetramers and pentamers identified by experimentally derived fold enrichment scores, as well as consensus sequences identified by PAR-CLIP analysis [21]. Whether this was done on the entire mRNA or the 3’UTR, the results were similar (Table 3). The most significant positive and negative correlations were obtained for % U- and % C-content, respectively. The combined % A+U as well as % G+U contents correlated positively. Oligonucleotides with highest correlation coeffients were U-rich sequences flanked by A or G, and the sequences GUUA and UUAA were most prominent among tetramers and pentamers. Of the consensus sequences identified by PAR-CLIP [21] the number of AGUUU sequences correlated also positively with AUF1 p42 enrichment in our 3’UTRs, but the GUUUG or UUAGA sequences did less. Similarly there was a positive correlation for the number of AUUUA or AUUUAA sequences and the ARE score. The correlation with the presence in the ARED Database was weak and no correlation was found with the RNA sequence length or Gorospe’s signature [50].

**Table 3:** Spearman's rank correlation coefficient of enrichment for specific sequence features.

Correlation of enrichment for	Only 3'UTR stat. value	p-value	Full mRNA stat. value	p-value
% of A nucleotides	0.102	2.37E-04	0.118	1.86E-05
% of C nucleotides	-0.249	<2.20E-16	-0.279	<2.20E-16
% of G nucleotides	-0.129	3.06E-06	-0.157	1.23E-08
% of U nucleotides	0.278	<2.20E-16	0.287	<2.20E-16
% of A + U nucleotides	0.231	<2.20E-16	0.246	<2.20E-16
% of G + U nucleotides	0.231	1.51E-16	0.246	<2.20E-16
number of UUA sequences	0.161	4.76E-09	0.108	9.26E-05
number of UUU sequences	0.140	4.03E-07	0.069	1.16E-02
number of UAA sequences	0.162	3.62E-09	0.113	4.22E-05
number of GUU sequences	0.139	4.32E-07	0.038	1.73E-01
number of UUG sequences	0.129	2.74E-06	0.019	4.93E-01
number of GUUA sequences	0.168	1.07E-09	0.109	7.57E-05
number of UUAA sequences	0.194	1.68E-12	0.159	7.38E-09
number of UUUU sequences	0.162	4.39E-09	0.117	2.25E-05
number of UUUA sequences	0.162	3.78E-09	0.127	3.95E-06
number of GUUU sequences	0.142	2.61E-07	0.073	8.15E-03
number of CGUUA sequences	0.088	1.43E-03	0.033	2.26E-01
number of GUUAA sequences	0.170	5.71E-10	0.141	3.07E-07
number of GUUAC sequences	0.119	1.51E-05	0.028	3.17E-01
number of CGAUA sequences	0.041	1.41E-01	0.017	5.42E-01
number of UUAAU sequences	0.182	3.46E-11	0.142	2.78E-07
number of UUGUU sequences	0.148	8.35E-08	0.119	1.54E-05
number of AGUUU sequences ^a^	0.135	9.64E-07	0.074	7.33E-03
number of GUUUG sequences ^a^	0.108	9.27E-05	0.015	5.91E-01
number of UUAGA sequences ^a^	0.094	6.73E-04	0.076	6.21E-03
number of AUUUA sequences	0.136	7.75E-07	0.113	4.58E-05
number of AUUUAA sequences	0.145	1.52E-07	0.158	1.01E-08
sequence length	0.036	1.54E-01	-0.101	2.54E-04
Gorospe signature ^b^	0.0054	8.86E-01	0.037	3.08E-01
ARE Scores ^c^	0.141	3.08E-07	nd	nd
ARED clusters ^d^	0.062	2.98E-02	nd	nd

Calculations are based on 508 enriched mRNAs and 800 non-enriched mRNAs. nd, not determined.

^a^ Refers to consensus motifs identified by PAR-CLIP analysis [21].

^b^ Refers to a previously published AUF1 binding motif [50].

^c^ Based on ARE*Score* [38] (see also Fig 3).

^d^ Refers to presence in the ARED Organism database [32, 33].

### GO-term analysis and comparison to other studies

To see whether enriched mRNAs code for specific protein classes, we analyzed our list at the DAVID Bioinformatics Resources [51]. There was no striking enrichment for any specific terms, but the highest scores of annotation clusters were obtained for “protein localization” and “regulation of cell death”. Whether this has any specific meaning remains unclear.

In addition we checked whether the human homologues of our enriched list overlap with published lists from two similar microarray studies [11, 20]. To our surprise we found no significant number of shared sequences between any of the three studies. Moreover, a direct comparison with the PAR-CLIP analysis of Yoon et al. [21] is difficult due to major technical differences. A limited number of 84 sequences of our list was found among 1365 targets with +-strand hits in 3’UTRs of the PAR-CLIP study. But as we do not know how good the genome-wide coverage of PAR-CLIP was, we cannot decide whether the overlap is significant.

### Identification of mRNA instability elements

**Fig 4:**
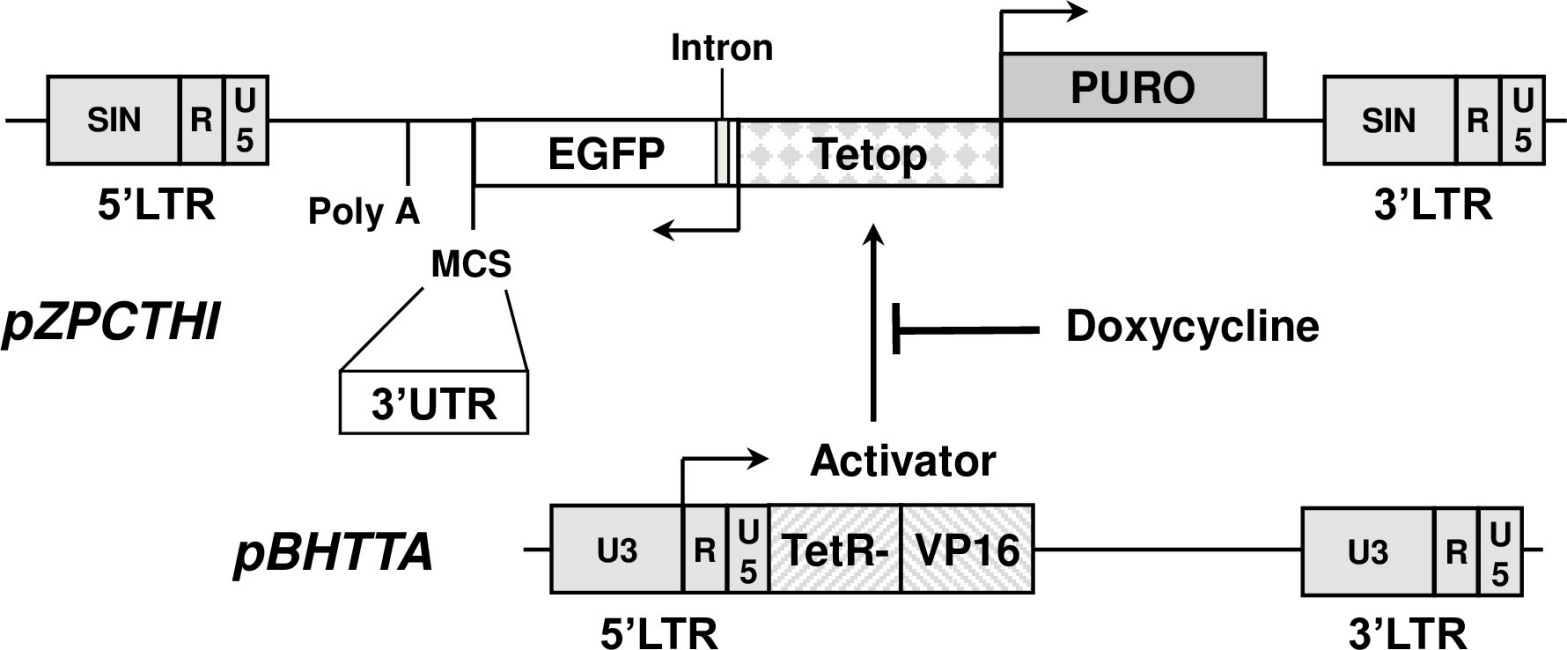
Tet-off vector-system used for RNA-decay measurements. A newly constructed retroviral vector pZPCTHI (for details see S1 Methods) comprises a 7x tet-operator [43] located between a CMV minimal promoter [43] to express the puromycin-resistance gene and a heterologous minimal promoter [44] to transcribe EGFP reporter constructs. Multiple cloning sites (MCS) for *Eco*RI, *Bgl*II and *Not*I immediately downstream of the GFP coding sequence serve to insert various 3'UTRs. A chimeric intron [52] was inserted into the EGFP 5’UTR. The polyadenylation signal is from the bovine growth hormone gene of vector pcDNA3 (Invitrogen). A second vector, pBHTTA, which encodes the tetracycline-sensitive transactivator protein (tTA) [43] was stably expressed in NIH/3T3 cells to generate a NIH/3T3-BHTTA cell line. All ZPCTHI constructs were subsequently tested in this cell line.

In order to identify mRNA instability elements, we constructed two retroviral pBabe-derived vectors, pZPCTHI and pBHTTA, that support the analysis of mRNA decay after Tet-off transcriptional inactivation (Fig 4). Various 3’UTRs of the most unstable mRNAs in Table 2 including those of human RANKL, KLF10, IL6, HES1, SMAD6 and BCL6 were fused to the stable EGFP reporter coding region of pZPCTHI and tested in NIH/3T3-pBHTTA cells. mRNA decay was measured after doxycycline addition inactivating the constitutive tetracycline-sensitive trans-activator. pZPCTHI without an insert showed an EGFP mRNA half-life of 400 min, whereas each of the inserted 3’UTRs reduced this half-life considerably. For IL6 [4] and BCL6 the EGFP-UTR constructs were as unstable as the endogenous mRNAs, whereas for RANKL and KLF10 they were somewhat more stable. For HES1 and SMAD6, the 3’UTR appeared to account only partially for the instability. In the case of HES1 we proceeded to test an additional construct including the HES1 coding region and 3’UTR in frame behind EGFP in the vector pZPCTHI_no_stop. This mRNA showed a half-life of 50.0 ± 12.2 min (3 experiments) and was clearly more unstable than with the 3’UTR alone (Table 2), indicating that HES1 mRNA has instability elements in the coding region.

Based on the premise that relevant instability elements should be conserved in evolution, we tested the EGFP-3’UTR with human UTRs on purpose. For reasons of verification and other projects at our Institute, we proceeded to verify the instability of RANKL mRNA with the mouse 3’UTR. The half-life was 58.3 ± 16.2 min (5 experiments) and thus very close to the one with the human 3’UTR (Table 2), suggesting an evolutionary conservation of mRNA decay determinants.

**Fig 5:**
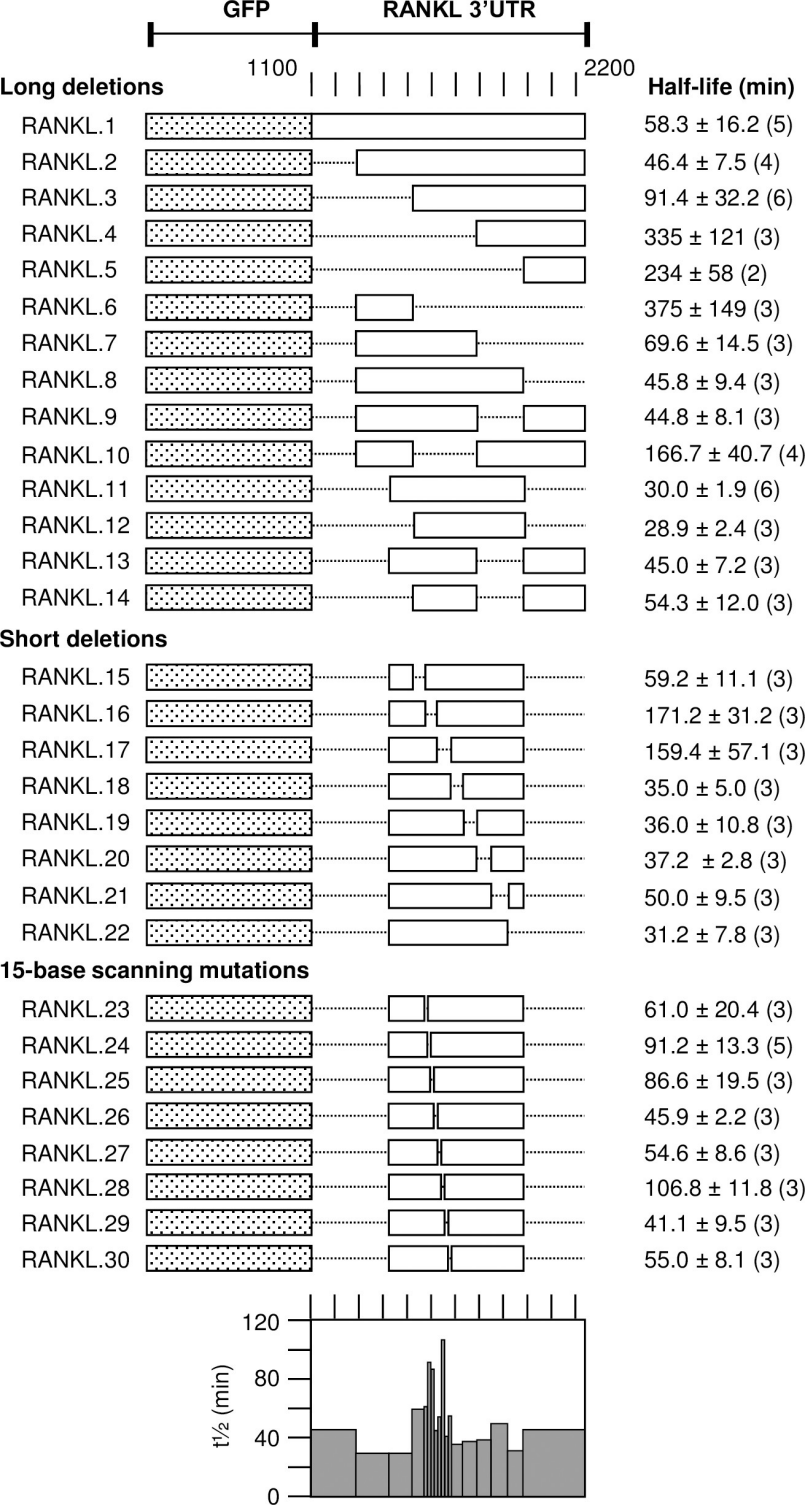
Identification of instability elements in RANKL mRNA. The 3’UTR of the mouse RANKL sequence 1106–2235 (NM_011613.3) with its own natural polyadenylation signal was amplified and inserted into pZPCTHI and transfected stably together with pBHTTA into NIH/3T3 cells. The EGFP-RANKL mRNA half-life was determined by inhibition of the transcriptional transactivator, measuring the mRNA level at 8, 68 and 128 min after doxycycline addition in independently transfected cell cultures, the number of which is indicated for each construct in parenthesis. The first order decay was calculated by linear regression of semi-logarithmic plots. The graphical representation at the bottom summarizes the half-lives for each construct and the increase of mRNA stability in specific mutants.

To identify the precise elements that confer instability, systematic deletions were made in the mouse RANKL and human BCL6 3’UTRs in the ZPCTHI vector (Fig 4) and tested whether they affect the short half-life. We initially tested large deletions of 200–250 bases, then small deletions of 50–70 bases within a reduced region and finally made linker scanning mutants of 15 bases. Each construct was stably transfected into mouse NIH/3T3 cells and the half-life measured using the Tet-off system. For the mouse RANKL 3’UTR we indentified two regions that affect the half-life in deletions pRANKL.16 and pRANKL.17 (Fig 5). Particularly, the sequences 1584–1613 (mutants pRANKL.23 and pRANKL.24) and 1644–1658 (mutant pRANKL.28) of mouse RANKL mRNA (NCBI reference sequence NM_011613.3) showed a significantly increased half-life when mutated. Both sequences were AU-rich, although not conforming to classical AREs. The first region showed a sequence conservation of 90% among all mammalian species analyzed (S2 Fig) and was present with 80% conservation in chicken. The second instability region was conserved to 93% among mammals analyzed (S2 Fig) and present in the chicken with 87% conservation. The two elements in RANKL mRNA are 30 bases apart. RNA-folding predictions showed no structural elements in this region. However, sequence alignment of the first instability element TTTTTATATAATGTCTAAAGTTATATTTCAGG with other unstable AUF1 targets revealed a statistically significant alignment with the SMAD6 3’UTR sequence TTTATATATTATATGGAAATATATATTATACT (position 2647–2673 of NM_008542.3) with 20 out of 27 nucleotides at the same position.

**Fig 6:**
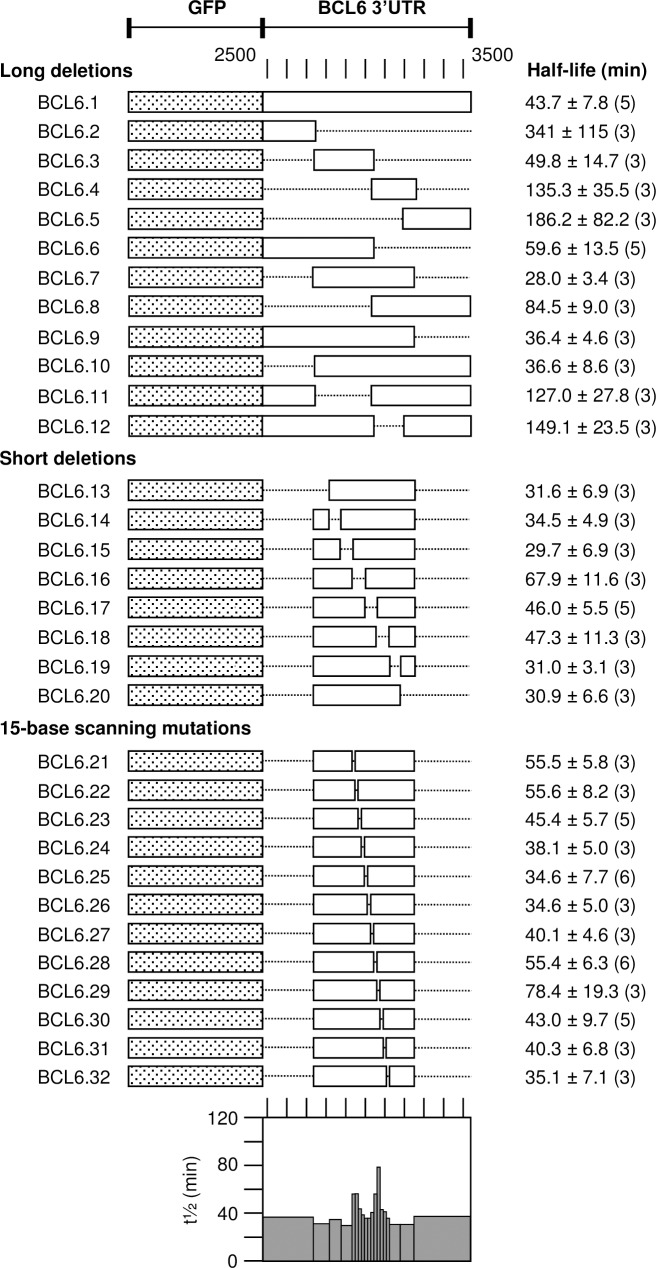
Identification of instability elements in BCL6. The 3’UTR of the human BCL6 sequence 2484–3529 (NM_001706.4) was amplified and inserted into pZPCTHI and stably transfected together with pBHTTA into NIH/3T3 cells. The sequence is followed by the polyadenylation signal of the bovine growth hormone gene. Mutants of the EGFP-BCL6 construct are described in detail in S3 Table and Materials and Methods. The mRNA half-life was determined by inhibition of the transcriptional transactivator, measuring the mRNA level at 8, 68 and 128 min after doxycycline addition in independently transfected cell cultures, the number of which is indicated for each construct in parenthesis. The first order decay was calculated by linear regression of semi-logarithmic plots. The graphical representation at the bottom summarizes the half-lives for each construct and the increase of mRNA stability in specific mutants.

For the human BCL6 3’UTR we also indentified two regions that affect the half-life contained within deletions pBCL6.16 to pBCL6.18 (Fig 6). The sequences 2946–2990 (mutants pBCL6.21 to pBCL6.23) and 3051–3080 (mutants pBCL6.28 and pBCL6.29) of human BCL6 mRNA (NCBI reference sequence NM_001706.4) showed a significantly increased half-life when mutated. The first sequence was highly AU-rich, at least in the second half, lacking again the precise signature of an ARE as defined in the ARED database. It was conserved to 95% among mammals analyzed (S2 Fig) and also present in chicken with some additionally inserted nucleotides. However, the second sequence had no AU-rich features at all. It was to 100% conserved among mammals analyzed (S2 Fig) and 70% in chicken. Analysis of the human sequence CUCGAUUUUGUAUCUGCAGGCAGACACGGAUCUGAG with the RNA-fold program predicted its folding into a hairpin structure that is conserved in the chicken. The two elements in BCL6 mRNA were separated by 60 bases.

### Analysis of in vivo RNA-protein interactions

**Fig 7:**
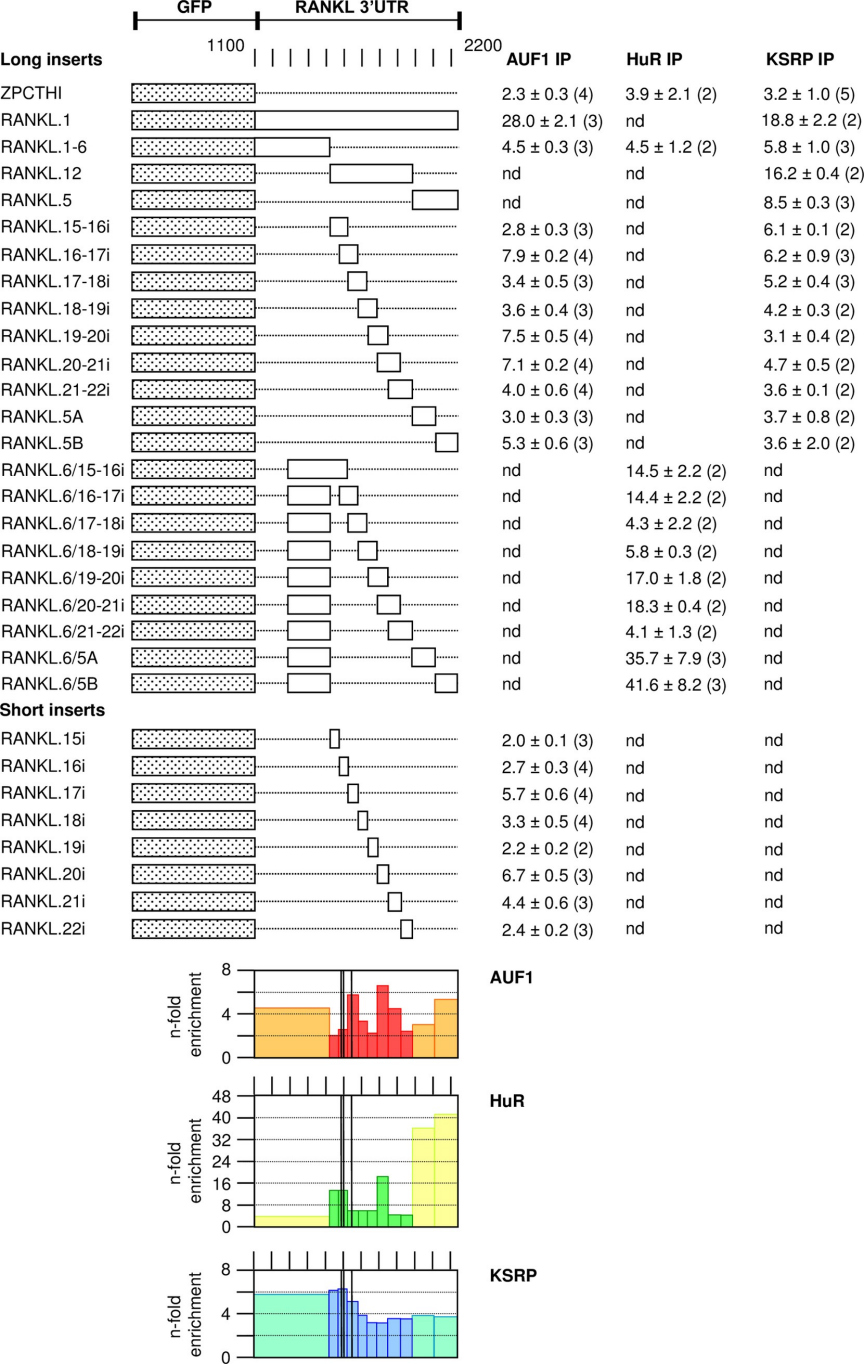
Binding-sites for AUF1 p37, HuR and KSRP in the mouse RANKL 3’UTR. Various pZPCTHI constructs with long or short inserts of the 3’UTR of mouse RANKL mRNA were transiently transfected with Lipofectamine 2000 into HEK 293T cells along with vectors to express tagged ARE-binding proteins. 21 h post transfection, cells were lysed and protein-bound RNA co-immunoprecipitated and recovered as shown in Fig 1 and described in Materials and Methods. RNA was isolated from input, microbead-adsorbed pellet and non-bound supernatant fractions, and EGFP and endogenous GAPDH mRNA quantified by RT-PCR. For each recombinant ARE-binding protein, the % adsorbed versus totally recovered RNA was calculated, and the relative enrichment of EGFP mRNA versus GAPDH mRNA expressed as “n-fold enrichment” ± standard deviation (number independent experiments). The graphs below show a graphical representation of the results. Vertical black bars indicate the location of instability elements.

We further analyzed whether AUF1 p37, HuR and KSRP, bind to the 3’UTRs of RANKL and BCL6 mRNA. EGFP-3’UTR constructs were transiently expressed from pZPCTHI together with appropriate protein expression vectors for myc-tagged AUF1 p37 [4], myc-tagged HuR or His-tagged KSRP. Experiments were carried out in human HEK293T cells that are more reliable than 3T3 cells in transient transfections. These cells only served as recipients for measuring the interaction of RNA constructs with tagged proteins in an intracellular environment. The tagged proteins were then isolated on magnetic beads by RNP-IP with antibodies directed against their tag as explained in Fig 1. Endogenous GAPDH mRNA that did not specifically bind to tested proteins was used as a control for normalization. Results were expressed as n-fold enrichment of EGFP mRNA on beads compared to GAPDH mRNA. Depending on the presence or absence of protein-binding sites in 3’UTR fragments, EGFP mRNA was better or less well co-precipitated. Presence of the total RANKL 3’UTR showed a strong enrichment with all three proteins (Fig 7). AUF1 p37 was most significant binding to 3’UTR fragments RANKL.17i and RANKL.20i, the first of which overlapped with sequences relevant for mRNA decay (indicated as black lines in the graphs below). In contrast, HuR interacted most strongly with the end of the 3’UTR, significantly with a region near RANKL.20i, and more weakly with a region common to RANKL.15-16i and RANKL.16-17i. This same region was also weakly bound by KSRP. In summary, HuR and KSRP may bind near the first instability element, while AUF1 p37 showed clear binding at the sequence 1628–1685 overlapping with the second instability element of mouse RANKL mRNA. This region is to 90% conserved in mammals analyzed (S2 Fig), and in its first 36 bases to 86% in the chicken. It comprises an AUUUA sequence in an AU-rich environment. The second clear binding site of AUF1 p37 at RANKL.20i lies outside the instability elements. It is U-rich and appears to coincide with a binding site for HuR. It is only to 57% conserved among the mammalian mRNAs aligned in S2 Fig.

**Fig 8:**
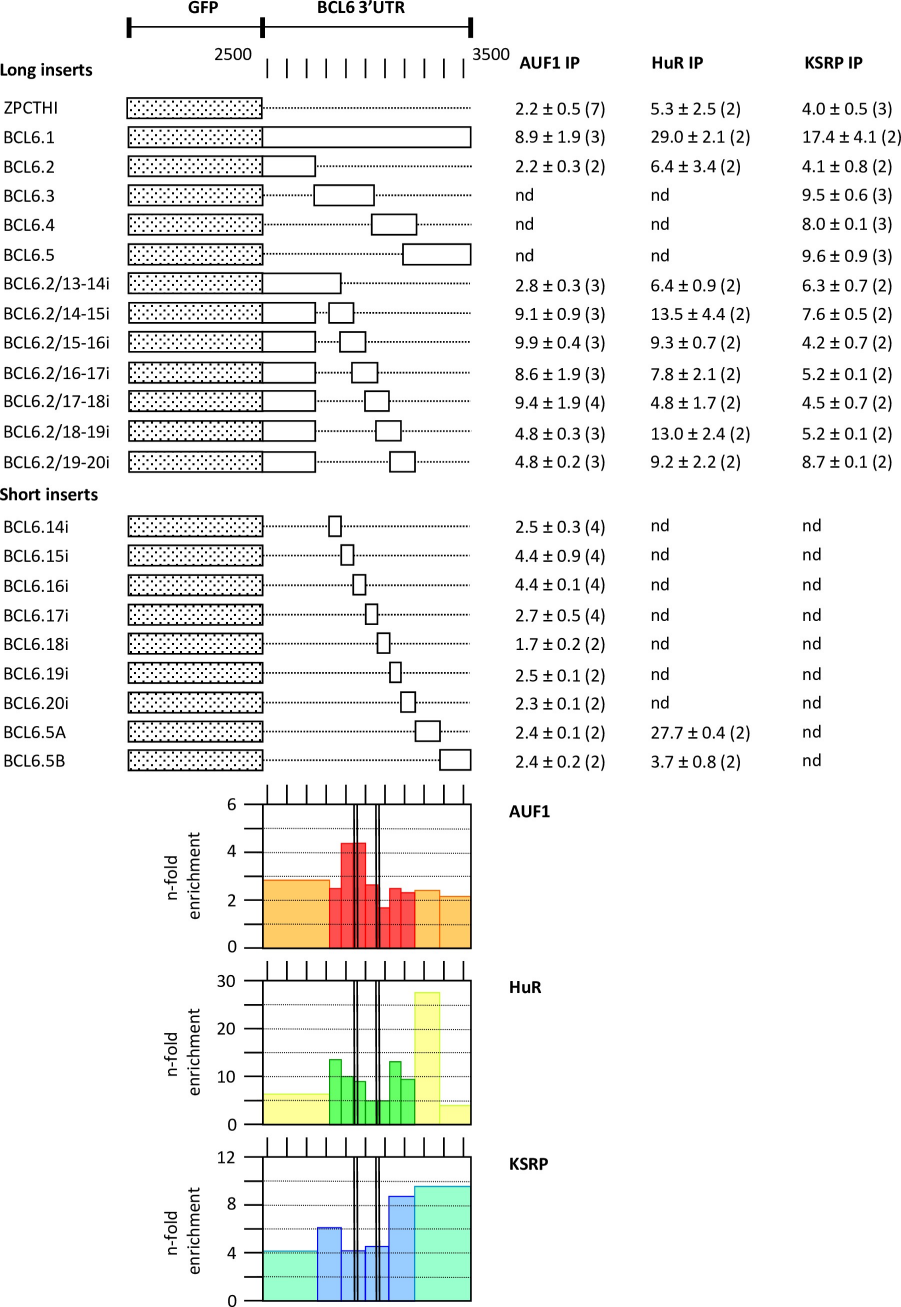
Binding-sites for AUF1 p37, HuR and KSRP in the human BCL6 3’UTR. Various pZPCTHI constructs with long or short inserts of the 3’UTR of human BCL6 mRNA were transiently transfected with Lipofectamine 2000 into HEK 293T cells along with vectors to express tagged ARE-binding proteins. 21 h post transfection, cells were lysed and protein-bound RNA co-immunoprecipitated and recovered as shown in Fig 1 and described in Materials and Methods. RNA was isolated from input, microbead-adsorbed pellet and non-bound supernatant fractions, and EGFP and endogenous GAPDH mRNA quantified by RT-PCR. For each recombinant ARE-binding protein, the % adsorbed versus totally recovered RNA was calculated, and the relative enrichment of EGFP mRNA versus GAPDH mRNA expressed as “n-fold enrichment” ± standard deviation (number independent experiments). The graphs below show a graphical representation of the results. Vertical black bars indicate the location of instability elements.

An identical survey was made for the human BCL6 3’UTR (Fig 8). Here again mainly AUF1 p37 showed a specific peak of RNP-IP with UTR transcripts of pBCL6.15i and pBCL6.16i at the sequence 2887–3011 that overlap with the first sequence of the instability region as determined in Fig 6. None of the proteins was convincingly bound to the second instability sequence. HuR and KSRP showed peaks of binding outside these sequences, mainly towards the end of the 3’UTR. The binding region of AUF1 p37 is conserved to 89% among 8 mammalian species analyzed (S2 Fig) and largely present in the chicken. In summary, both in RANKL and BCL6 mRNA, one of the two mRNA instability sequences (S2 Fig) overlapped with a strong AUF1 p37 binding site (Figs 7 and 8).

### Further analysis of instability elements and AUF1 p37-binding regions

To test whether the instability elements and AUF-binding regions of RANKL and BCL6 3’UTRs studied here and of IL6 3’UTR studied previously [4] are representative for similar elements in other unstable mRNAs, we carried out optimal sequence alignments. Test sequences corresponding to instability elements and AUF1 binding sites (S8 Table) were aligned with the UTRs of all unstable mRNAs with a half-life of ≤2h present in our AUF1 p42-bound mRNA list. We added to this analysis some well-established 3’UTRs with typical AREs, some 3’UTRs that were previously studied as AUF1-targets and some 3’UTRs with the potential of being degraded by Zc3h12a endonuclease (S9 Table). A potential stem-loop that confers instability in IL6 mRNA [4] was postulated to be the target of Zc3h12a endonuclease (also known as monocyte chemo-attractant protein-induced protein 1 (MCPIP1) or Regnase-1)[53, 54]. The same RNase was implicated in the degradation of IL-12β mRNA [53], IL-1β mRNA [55], IL-2 mRNA [56], and possibly other upregulated mRNAs including c-Rel, Ox40, TnfR2, and Icos, in Regnase-1 knock-out mice [57].

Sequence alignments with statistic significance are reported in S10 Table. Most strikingly, each tested sequence showed similarities with just a few 3’UTRs. RANKL instability element 1 aligned best with AU-rich sequences in Smad6, Gadd45a [11], and classical AREs of Csf2 3’UTR [26]. RANKL instability element 2 showed highest similarity with AU-rich 3’UTR sequences of Gadd45a, Hivep2 and Myc mRNA. We tentatively conclude that these instability elements have characteristics shared with AREs although they contain no classical AUUUA sequences. BCL6 instability element 1 showed good alignments with sequences of Suv420h1, Bmp4, Tnfaip1, and Cdkn2a, but these sequences did not show any good alignments between each other. The IL6 element 2, which itself has features of AREs, aligned readily with a large number of 3’UTRs usually at ARE-like regions some of which are known as instability regions, for example in mRNA of Ptgs2, Csf2, Tnf, or Fos. In contrast, the two instability elements with non-AU-rich sequences but potential stem-loop structures, the BCL6 element 2 and IL6 element 1 [4], generated only few significant alignments for which we found no evidence of conservation of potential hairpin structures. Overall, besides RANKL and BCL6 mRNA, only 16 of the 30 unstable AUF1 p42-binding mRNAs of our study aligned with one of the test sequences. In conclusion, besides the AU-richness, we found no evidence for a specific shared sequence element among the unstable or AUF1-bound 3’UTRs.

## Discussion

We provide here information on target mRNAs of AUF1 p42 in mouse NIH/3T3 cells using RNP-IP followed by microarray analysis of cDNA derived of bound mRNA. Two previous microarray studies have taken a similar approach, but have generated a different set of data each [11, 20]. The poor overlap of results may have its cause in technical differences. Unlike in our study where we over-expressed myc-tagged AUF1 p42, did not UV-crosslink RNPs, and used antibodies against the myc-tag, Lal et al. (2004) isolated AUF1 target mRNAs from UV-crosslinked cytoplasmic RNPs of human HeLa cells with polyclonal anti-AUF1 antibodies against all 4 isoforms [11]. We also noticed overall much lower Z-scores in the study of Lal et al. than those calculated for our enriched mRNAs suggesting a less effective selection. Wu et al. (2013) have analyzed enriched RNAs by incubating an *in vitro* transcribed human cDNA library with recombinant His-AUF1 p37 and isolating RNPs with anti-His-tag antibodies [20]. The *in vitro* assembly may have generated different interactions than those observed for RNPs *in vivo*. The more recent approach of PAR-CLIP [21] is impressive and certainly more direct towards the goal of identifying AUF1 binding sites than our present approach. However, as PAR-CLIP identifies target mRNAs on the basis of the frequency of short sequence runs at cross-linked binding sites, the coverage might be a limitation in identifying mRNAs with low expression levels. This might explain why RANKL, BCL6 or HES1 do not appear in the PAR-CLIP data set [21]. Alternatively, specific gene expression in different cell lines may also cause discrepancies.

Our study reveals that AUF1 p42 binds to many different mRNAs with a preference for U-rich sequences flanked by A or G residues (Table 3). The motives with the highest significant enrichment are compatible both with studies that concluded on AUF1 binding to AREs [2, 4] and with the PAR-CLIP study [21] that identified GU-rich consensus sequences for AUF1 target sites. In spite of a moderate enrichment for classical AUUUA-motives by anti-AUF1 RNP-IP (see ARE*Score* analysis [38] in Fig 3), we conclude that AUF1 p42 has a broader binding specificity. In accordance only a limited number of enriched mRNAs were represented in the ARED Organism database [32, 33]. AUUUA was clearly less enriched than in a similar microarray study for TTP-bound targets [38, 58] and thus the classical ARE may have a higher affinity for TTP. In spite of progress in defining new mRNA targets, the precise specificity range of most RNA-binding proteins remains largely unsolved. Pentameric motives as those identified in Table 3 are rather redundant and certainly insufficient to predict AUF1-binding sites. It is therefore essential to define binding sites experimentally as done here.

We cannot exclude that there are a certain number of false positive mRNAs in our list, mainly because we overexpressed the myc-tagged AUF1 p42. This may provoke complexes with lower affinity targets, and at the same time saturate real target sites that may become more easily detectable. The potential distortion resulting from this is difficult to estimate. We do not know, how low-affinity complexes in absence of cross-linking survive washing steps after co-immunoprecipitation. As it is technically convenient to use tagged proteins expressed from inducible vectors both for the specificity of antibodies and for being able to estimate the background of non-specific co-precipitation, others have similarly adopted overexpression of tagged AUF1 isoforms in their approach [21].

An important goal of this study was to find out whether RNP-IP of myc-AUF1 p42 enriched for unstable mRNAs. In absence of genome-wide information on mRNA stability in NIH/3T3 cells, we annotated our AUF1 p42 target list with half-life data from ES cells [37]. We consider this justified because RNA instability is often a conserved feature among mammalian species and tissues. In addition we found a strong correlation for ES-cell mRNA half-lives and half-lives of 20 mRNAs tested in our study (p<0.001) (Table 2). Following the assumption that the ES cell data are representative, only about 7.5% of AUF1-bound mRNAs are unstable. Thus, it seems unlikely that binding of AUF1 *per se* can be sufficient to induce mRNA instability. This is not surprising, as AUF1 interacts with RNA through RRM motifs but lacks enzymatic activity. Unstable AUF1-bound mRNAs probably comprise additional sequences or structures to explain their instability.

Taking advantage of newly designed tet-off vectors we analyzed three particularly unstable mRNAs for their instability elements. We conclude that Hes1 mRNA comprises an instability element in the coding region while instability regions of RANKL and BCL6 mRNA are in their 3’UTRs. We show newly that RANKL and BCL6 mRNA have each two instability regions separated by 30 to 60 bases, one of which is AU-rich and overlaps with an AUF1 p37 binding site. The existence of two or more instability regions in a single mRNA has not been widely high-lighted so far, but might be frequent in mRNA decay. We have previously identified a similar pattern in the human IL6 3’UTR, where a destabilizing AUF1-binding site was within about 50 bases of a second destabilizing element with a putative stem-loop structure [4].

The easiest way to explain our instability data of RANKL and BCL6 UTRs, is to postulate that two independent elements contribute each partially to mRNA instability. This idea is entirely compatible with our deletion analysis where missing one of the two elements makes mRNA half-way more stable (Figs 5 and 6). Based on the instability sequences observed in RANKL and BCL6 mRNA (S2 Fig), or in IL6 mRNA previously [4], we conclude that they can be distinct in nature: either a non-AU-rich sequence with the potential of forming a RNA stem-loop, or short U-rich sequences flanked by A or G nucleotides without necessarily conforming to a classical AUUUA motif. Each of these might independently favor a degradation pathway.

The unstable mRNAs studied here were previously only partially analyzed for instability elements. HES1 is a basic helix-loop-helix transcription factor, which regulates Notch signaling proteins that are central in development and cancer. HES1 acts as a repressor with oscillations of 2 h periodicity. These oscillations result from negative autoregulation of HES1 mRNA transcription and rapid HES1 degradation by the proteasome implicating that both the mRNA and protein are highly unstable with half-lives below 25 min [59]. HES1 oscillation is thought to be essential for maintaining neural progenitors in embryonic brain [60]. HES1 mRNA is itself negatively regulated by miR-9 that binds to the 3’UTR [61, 62]. Overexpression of miR-9 reduces HES1 mRNA half-life to 20 min while miR-9 knockdown increases it to about 40 min and dampens HES1 oscillations [61, 62]. Here we confirm the HES1 mRNA instability, but show newly that the 3’UTR does only partially account for it, and that additional coding region determinants are important. Thus, HES1 mRNA appears to belong to a group of mRNAs, the stability of which is also controlled during translation, like c-Fos and c-Myc mRNA [63, 64]. The precise elements in the coding region remain to be defined.

RANKL is a cell surface protein with important functions in bone formation [65] and mammary gland development [66]. RANKL mRNA was previously found to be unstable in ES cells [37] and more recently in the mammary gland [66] where its stability is influenced by progesterone through an unknown mechanism. Our identification of two AU-rich instability elements may help us to investigate this further.

The proto-oncogene BCL6 is a repressing transcription factor [67]. Mainly due to chromosomal translocations it is often misregulated in B cell lymphomas [68]. The instability of BCL6 mRNA was noticed previously [36, 37] but not further investigated. Our results now indicate two 3’UTR instability elements composed of an AU-rich region and a highly conserved adjacent non-AU-rich element with the potential of forming a hairpin.

The pathways which lead to the rapid degradation of RANKL and BCL6 mRNA need to be further studied. For RANKL mRNA one may speculate that even in absence of a classical AUUUA sequence at the two instability elements, these sequences are highly AU-rich and may conform to degradation pathways of classical ARE-containing mRNAs that are thought to be initiated by poly-A tail shortening followed by a 3’ to 5’ exonuclease attack [69]. In BCL6 mRNA one of the two elements may trigger the same pathway. In addition, BCL6 mRNA, as IL6 mRNA [4] has a second non-AU-rich element with the potential to form RNA hairpin, a feature also postulated for instability elements of IL-2 [56] and c-Rel mRNA [57]. Such elements have been postulated to be targets of Zc3h12a (MCPIP1, Regnase-1) [53, 54], which may exert effects on RNA stability either as a riboendonuclease or by translational silencing [70]. Whether the BCL6 hairpin element is a target of this protein remains to be investigated. Alignments of the BCL6 hairpin element with the unstable mRNAs of this study or with other postulated mRNA targets of Zc3h12a, such as IL-12β mRNA [53], IL-1β mRNA [55], IL-2 mRNA [56], and c-Rel [57] showed no clear sequence homologies (S10 Table). Thus, we cannot find a conserved element as for example the hairpin of iron-responsive elements [71] that might serve as a shared recognition element in mRNA decay.

We notice that in each of our carefully dissected 3’UTRs at least one AU-rich instability element overlaps with a preferential binding site for AUF1 p37. Possibly, these AU-rich sequences may interact as well with other AU-binding proteins. While we have not found any preferred binding for HuR or KSRP at these sites, we were missing a functional tagged TTP for our assays. We also found little evidence for an overlap of specificity comparing our list with microarray data obtained by others for KSRP [45], HuR [11], and TTP [58], suggesting that targets are indeed separate. As shown by others, binding sites for AUF1 and HuR, dependent on the target, can either be distinct or concurrent [11, 21].

We have no clues why AU-rich sequences near or at the AUF1 p42 binding sites of RANKL, BCL6 and IL6 mRNA contribute to instability and in what respect they differ from other, non-destabilizing AUF1 binding sites. Preliminary experiments with AUF1 siRNA suggest that AUF1 contributes to BCL6 mRNA instability, similar to our previous findings on IL6 mRNA [4]. A possible direct role of AUF1 in mRNA destabilization seems plausible as previously shown after AUF1 knock-down [20] or AUF1 deletion in mice [13]. AUF1 was reported to interact directly with exosomal components [72, 73] or to cooperate with Argonaute 2 in the degradation of a subset of mRNAs [20]. We have noticed three potential miR-410 seed sequences (TTATAT) in the RANKL instability elements and similarly two potential seed sequences (GGCAGA) of mIR-346 in the BCL6 instability elements. Others have implicated mIR-142-3p in the regulation of IL6 mRNA [74] with interaction sites at or near the putative stem-loop instability element [4]. Such predictions require further experimental testing in the case of RANKL and BCL6 mRNA. Alternatively, AUF1 was shown to act as a regulator of translation, impeding or promoting polysome size depending on the target [21].

In conclusion, AUF1 binding may be sufficient in certain mRNAs to provoke mRNA instability, but not all AUF1-bound mRNAs are unstable and there must be additional features that decide on rapid mRNA decay. It seems possible that this is due to two or more instability elements as uncovered here. It remains to be seen whether these elements play a role in facilitating endonuclease attack or rather ARE-mediated mRNA decay that starts with deadenylation.

## Supporting information

S1 Methods Details of plasmids for mRNA half-life measurements by the Tet-off system. (DOC)

S1 Fig mRNA half-life of a selected set of enriched mRNAs that bind to AUF1 p42. Mouse 3T3 cells were exposed to medium with 6 μg/ml actinomycin D at time-point 0. mRNA was isolated at 0, 30, 60 and 120 min and individual mRNAs, including mouse GAPDH mRNA as a control, quantified by RT-PCR. Values are of two independent experiments. Note that the log-scale was extended to 1% in the case of Hes1 and Smad6 mRNA. Data of IL-6 mRNA were previously published [4]. Half-life values were calculated by linear regression and are reported in Table 2.
(TIF)

S2 Fig 3’UTR sequence alignment of instability regions of mouse RANKL and human BCL6 mRNA. For **A**, the sequence 1539–1717 of mouse RANKL (NM_011613.3) was aligned by ClustalW with corresponding RANKL sequences of human (NM_003701.3), bovine (NM_001205770.1), dog (XM_846672.2), killer whale (XM_004274560.1), pig (XM_001925694.4) and sheep (XM_004012051.1). For **B**, the sequence 2877–3112 of human BCL6 (NM_001706.4), was aligned by ClustalW with corresponding RANKL sequences of mouse (NM_009744.3), bovine (NM_001206450.1), horse (XM_003991804.1), dog (XM_005639719.1), killer whale (XM_004278481.1), sheep (XM_004003049.1) and *Dasypus novemcinctus* (nine-banded armadillo; XM_004467199.1). Stars below the alignments indicate full sequence conservation. Elements that confer instability are highlighted in bold and a preferred AUF1-binding region is shown as a grey background.
(TIF)

S1 Table Primers used for 3'UTR amplification and subcloning into pZPCTHI. Numbering is based on NCBI reference sequences. Bold letters indicate restriction sites used for cloning. When the natural polyadenylation site is absent, a site in the vector is used for poladenylation.
(PDF)

S2 Table Primers used for 3'UTR deletion mutants of mouse RANKL. Numbering is based on NCBI reference sequence NM_011613.3. Bold letters indicate restriction sites used for cloning or linker sequence in scanning mutants.
(PDF)

S3 Table Primers used for amplification of 3'UTR fragments of human BCL6. Numbering is based on NCBI reference sequence NM_001706.4. Bold letters indicate restriction sites used for cloning or linker sequence in scanning mutants.
(PDF)

S4 Table Primers used for amplification of 3'UTR fragments of mouse RANKL. Numbering is based on NCBI reference sequence NM_011613.3. Bold letters indicate restriction sites used for cloning.
(PDF)

S5 Table Primers used for amplification of 3'UTR fragments of human BCL6. Numbering is based on NCBI reference sequence NM_001706.4. Bold letters indicate restriction sites used for cloning.
(PDF)

S6 Table Primers used for microarray data confirmation and endogenous half-life measurements by RT-PCR. (PDF)

S7 Table Complete list of mRNAs of mouse NIH/3T3 cells enriched by RNP-IP of myc-AUF1 p42. The list comprises all mRNAs that were more than 3-fold enriched on microarrays, sorted according to the n-fold enrichment. The ARE score and number of AUUUA elements in the 3'UTR was determined by the program ARE*Score* (Spasic et al., 2012)[38]. The ARED cluster refers to the presence in the ARED Organism database (Halees et al., 2008)[33]. N/A, not available, indicates mRNAs not found in the ARED database. mRNA decay rates known prior to this study in mouse ES cells (a) (Sharova et al., 2009)[37], or for human homologs in lymphocytes (b) (Raghavan et al., 2002)[35] and HepG2 cells (c) (Yang et al., 2003)[36], or known from other studies (d) (Paschoud et al., 2006)[4]. Known half-lives >3h in Sharova et al. (2009)[37] are reported as stable. Original data can be obtained from the authors.
(PDF)

S8 Table Test sequences used for optimal local sequence alignments between instability elements and selected 3’UTRs. The test sequences correspond to the instability elements (pink sequences) determined for mouse Rankl mRNA and human BCL6 mRNA in this study, and for human IL6 mRNA in a previous study (Paschoud et al., 2006)[4]. They include adjacent AUF1-binding regions. In additon a mouse Smad6 sequence with a predicted good alignment with the mouse Rankl A test sequence was used as a test sequence. Each test sequence was aligned with 3'UTRs of mRNAs listed in S9 Table.
(PDF)

S9 Table Target 3'UTR sequences used in optimal local sequence alignments. These target sequences were aligned with the test sequences of S8 Table. They correspond to 3'UTRs of the most unstable mRNAs of this study (S7 Table), to 3'UTRs of mRNAs previously recognized as unstable, to known binding targets of AUF1, or to potential targets of Zc3h12a. mRNA half-lives are either known prior to this study in mouse ES cells (a) (Sharova et al., 2009)[37], or for human homologs in lymphocytes (b) (Raghavan et al., 2002)[35] and HepG2 cells (c) (Yang et al., 2003)[36], or known from other studies (d) (Paschoud et al., 2006)[4], (e) (Shaw and Kamen, 1986)[26], (f) (Li et al., 2012)[56], (h) (Gorospe et al., 1993), or were determined in this study (g). For Cdkn2a, Rel, and Zfp266 the alignment between mouse and human UTRs were very poor.
(PDF)

S10 Table Significant local sequence alignments in 3'UTRs. 3'UTRs of mouse mRNAs listed in S9 Table were aligned with specific instability elements and AUF1-binding regions described in detail in S8 Table. Most alignments are in regions with conservation between mouse and human mRNAs.
(PDF)

## References

[1] Keene JD, Tenenbaum SA. Eucaryotic mRNPs may represent posttranscriptional operons. Mol Cell. 2002;9:1161–7. 12086614 10.1016/s1097-2765(02)00559-2

[2] Zhang W, Wagner BJ, Ehrenman K, Schaefer AW, DeMaria CT, Crater D, et al Purification, characterization, and cDNA cloning of an AU-rich element RNA-binding protein, AUF1. Mol Cell Biol. 1993;13:7652–65. 8246982 10.1128/mcb.13.12.7652-7665.1993 PMC364837

[3] Wagner B J., DeMaria C T., Sun Y, Wilson G, M., Brewer G. Structure and genomic organization of the human AUF1 gene: alternative pre-mRNA splicing generates four protein isoforms. Genomics. 1998;48:195–202. 10.1006/geno.1997.5142 9521873

[4] Paschoud S, Dogar AM, Kuntz C, Grisoni-Neupert B, Richman L, Kühn LC. Destabilization of interleukin-6 mRNA requires a putative RNA stem-loop structure, an AU-rich element and the RNA-binding protein AUF1. Mol Cell Biol. 2006;26:8228–41. 10.1128/MCB.01155-06 16954375 PMC1636780

[5] Zucconi B, Ballin J, Brewer B, Ross C, Huang J, Toth E, et al Alternatively expressed domains of AU-rich element RNA-binding protein 1 (AUF1) regulate RNA-binding affinity, RNA-induced protein oligomerization, and the local conformation of bound RNA ligands. J Biol Chem. 2010;285:39127–39. 10.1074/jbc.M110.180182 20926381 PMC2998080

[6] Neininger A, Kontoyiannis D, Kotlyarov A, Winzen R, Eckert R, Volk HD, et al MK2 targets AU-rich elements and regulates biosynthesis of tumor necrosis factor and interleukin-6 independently at different post-transcriptional levels. J Biol Chem. 2002;277:3065–8. 10.1074/jbc.C100685200 11741878

[7] Winzen R, Kracht M, Ritter B, Wilhelm A, Chen CY, Shyu AB, et al The p38 MAP kinase pathway signals for cytokine-induced mRNA stabilization via MAP kinase-activated protein kinase 2 and an AU-rich region-targeted mechanism. EMBO J. 1999;18:4969–80. 10.1093/emboj/18.18.4969 10487749 PMC1171568

[8] Wilson GM, Lu J, Sutphen K, Suarez Y, Sinha S, Brewer B, et al Phosphorylation of p40AUF1 regulates binding to A + U-rich mRNA-destabilizing elements and protein-induced changes in ribonucleoprotein structure. J Biol Chem. 2003;278:33039–48. 10.1074/jbc.M305775200 12819194

[9] Loflin P, Chen CY, Shyu AB. Unraveling a cytoplasmic role for hnRNP D in the in vivo mRNA destabilization directed by the AU-rich element. Genes Dev. 1999;13:1884–97. 10421639 10.1101/gad.13.14.1884 PMC316883

[10] Sarkar B, Xi Q, He C, Schneider RJ. Selective degradation of AU-rich mRNAs promoted by the p37 AUF1 protein isoform. Mol Cell Biol. 2003;23:6685–93. 10.1128/MCB.23.18.6685-6693.2003 12944492 PMC193711

[11] Lal A, Mazan-Mamczarz K, Kawai T, Yang X, Martindale JL, Gorospe M. Concurrent versus individual binding of HuR and AUF1 to common labile target mRNAs. EMBO J. 2004;23:3092–102. 10.1038/sj.emboj.7600305 15257295 PMC514922

[12] Raineri I, Wegmueller D, Gross B, Certa U, Moroni C. Roles of AUF1 isoforms, HuR and BRF1 in ARE-dependent mRNA turnover studied by RNA interference. Nucleic Acids Res. 2004;32:1279–88. 10.1093/nar/gkh282 14976220 PMC390274

[13] Lu J-Y, Sadri N, Schneider RJ. Endotoxic shock in AUF1 knockout mice mediated by failure to degrade proinflammatory cytokine mRNAs. Genes Dev. 2006;20:3174–84. 10.1101/gad.1467606 17085481 PMC1635151

[14] Carballo E, Lai WS, Blackshear PJ. Feedback inhibition of macrophage tumor necrosis factor-alpha production by tristetraprolin. Science. 1998;281:1001–5. 9703499 10.1126/science.281.5379.1001

[15] Stoecklin G, Colombi M, Raineri I, Leuenberger S, Mallaun M, Schmidlin M, et al Functional cloning of BRF1, a regulator of ARE-dependent mRNA turnover. EMBO J. 2002;21:4709–18. 10.1093/emboj/cdf444 12198173 PMC126184

[16] Lai WS, Kennington EA, Blackshear PJ. Tristetraprolin and its family members can promote the cell-free deadenylation of AU-rich element-containing mRNAs by poly(A) ribonuclease. Mol Cell Biol. 2003;23:3798–812. 10.1128/MCB.23.11.3798-3812.2003 12748283 PMC155217

[17] Gherzi R, Lee KY, Briata P, Wegmüller D, Moroni C, Karin M, et al A KH domain RNA binding protein, KSRP, promotes ARE-directed mRNA turnover by recruiting the degradation machinery. Mol Cell. 2004;14:571–83. 10.1016/j.molcel.2004.05.002 15175153

[18] Xu N, Chen CY, Shyu AB. Versatile role for hnRNP D isoforms in the differential regulation of cytoplasmic mRNA turnover. Mol Cell Biol. 2001;21:6960–71. 10.1128/MCB.21.20.6960-6971.2001 11564879 PMC99872

[19] Chen CY, Xu N, Zhu W, Shyu AB. Functional dissection of hnRNP D suggests that nuclear import is required before hnRNP D can modulate mRNA turnover in the cytoplasm. RNA. 2004;10:669–80. 10.1261/rna.5269304 15037776 PMC1370557

[20] Wu X, Chesoni S, Rondeau G, Tempesta C, Patel R, Charles S, et al Combinatorial mRNA binding by AUF1 and Argonaute 2 controls decay of selected target mRNAs. Nucleic Acids Res. 2013;41:2644–58. 10.1093/nar/gks1453 23303783 PMC3575833

[21] Yoon JH, De S, Srikantan S, Abdelmohsen K, Grammatikakis I, Kim J, et al PAR-CLIP analysis uncovers AUF1 impact on target RNA fate and genome integrity. Nat Commun. 2014;5:5248 10.1038/ncomms6248 25366541 PMC4291169

[22] Wilson T, Treisman R. Removal of poly(A) and consequent degradation of c-fos mRNA facilitated by 3' AU-rich sequences. Nature. 1988;336:396–9. 10.1038/336396a0 3194021

[23] Greenberg ME, Shyu AB, Belasco JG. Deadenylylation: a mechanism controlling c-fos mRNA decay. Enzyme. 1990;44:181–92. 2133650 10.1159/000468756

[24] Xu N, Chen CY, Shyu AB. Modulation of the fate of cytoplasmic mRNA by AU-rich elements: key sequence features controlling mRNA deadenylation and decay. Mol Cell Biol. 1997;17:4611–21. 9234718 10.1128/mcb.17.8.4611 PMC232314

[25] Treisman R. Transient accumulation of c-fos RNA following serum stimulation requires a conserved 5' element and c-fos 3' sequences. Cell. 1985;42:889–902. 2414012 10.1016/0092-8674(85)90285-5

[26] Shaw G, Kamen R. A conserved AU sequence from the 3' untranslated region of GM-CSF mRNA mediates selective mRNA degradation. Cell. 1986;46:659–67. 3488815 10.1016/0092-8674(86)90341-7

[27] Lagnado CA, Brown CY, Goodall GJ. AUUUA is not sufficient to promote poly(A) shortening and degradation of an mRNA: the functional sequence within AU-rich elements may be UUAUUUA(U/A)(U/A). Mol Cell Biol. 1994;14:7984–95. 7969138 10.1128/mcb.14.12.7984-7995.1994 PMC359337

[28] Zubiaga AM, Belasco JG, Greenberg ME. The nonamer UUAUUUAUU is the key AU-rich sequence motif that mediates mRNA degradation. Mol Cell Biol. 1995;15:2219–30. 7891716 10.1128/mcb.15.4.2219 PMC230450

[29] Putland RA, Sassinis TA, Harvey JS, Diamond P, Coles LS, Brown CY, et al RNA destabilization by the granulocyte colony-stimulating factor stem-loop destabilizing element involves a single stem-loop that promotes deadenylation. Mol Cell Biol. 2002;22:1664–73. 10.1128/MCB.22.6.1664-1673.2002 11865046 PMC135610

[30] Stoecklin G, Lu M, Rattenbacher B, Moroni C. A constitutive decay element promotes tumor necrosis factor alpha mRNA degradation via an AU-rich element-independent pathway. Mol Cell Biol. 2003;23:3506–15. 10.1128/MCB.23.10.3506-3515.2003 12724409 PMC164766

[31] Mawji IA, Robb GB, Tai SC, Marsden PA. Role of the 3'-untranslated region of human endothelin-1 in vascular endothelial cells. Contribution to transcript lability and the cellular heat shock response. J Biol Chem. 2004;279:8655–67. 10.1074/jbc.M312190200 14660616

[32] Bakheet T, Williams BR, Khabar KS. ARED 3.0: the large and diverse AU-rich transcriptome. Nucleic Acids Res. 2006;34:D111–4. 10.1093/nar/gkj052 16381826 PMC1347415

[33] Halees AS, El-Badrawi R, Khabar KSA. ARED Organism: expansion of ARED reveals AU-rich element cluster variations between human and mouse. Nucleic Acids Res. 2008;36:D137–40 10.1093/nar/gkm959 17984078 PMC2238997

[34] Lam LT, Pickeral OK, Peng AC, Rosenwald A, Hurt EM, Giltnane JM, et al Genomic-scale measurement of mRNA turnover and the mechanisms of action of the anti-cancer drug flavopiridol. Genome Biol. 2001;2:RESEARCH0041 11597333 10.1186/gb-2001-2-10-research0041 PMC57796

[35] Raghavan A, Ogilvie RL, Reilly C, Abelson ML, Raghavan S, Vasdewani J, et al Genome-wide analysis of mRNA decay in resting and activated primary human T lymphocytes. Nucleic Acids Res. 2002;30:5529–38. 12490721 10.1093/nar/gkf682 PMC140061

[36] Yang E, van Nimwegen E, Zavolan M, Rajewsky N, Schroeder M, Magnasco M, et al Decay rates of human mRNAs: correlation with functional characteristics and sequence attributes. Genome Res. 2003;13:1863–72. 10.1101/gr.1272403 12902380 PMC403777

[37] Sharova LV, Saharov AA, Nedorezov T, Piao Y, Shaik N, Ko MSH. Database for mRNA half-life of 19 977 genes obtained by DNA microarray analysis of pluripotent and differentiating mouse embryonic stem cells. DNA Res. 2009;16:45–58. 10.1093/dnares/dsn030 19001483 PMC2644350

[38] Spasic M, Friedel C, Schott J, Kreth J, Leppek K, Hofmann S, et al Genome-wide assessment of AU-rich elements by the AREScore algorithm. PLoS Genetics. 2012;8:e1002433 10.1371/journal.pgen.1002433 22242014 PMC3252268

[39] Jordan M, Schallhorn A, Wurm F. Transfecting mammalian cells: Optimization of critical parameters affecting calcium-phosphate precipitate formation. Nucleic Acids Res. 1996;24:596–601. 8604299 10.1093/nar/24.4.596 PMC145683

[40] Irizarry RA, Hobbs B, Collin F, Beazer-Barclay YD, Antonellis KJ, Scherf U, et al Exploration, normalization, and summaries of high density oligonucleotide array probe level data. Biostat. 2003;4:249–64. 10.1093/biostatistics/4.2.249 12925520

[41] Smyth GK. Linear models and empirical Bayes methods for assessing differential expression in microarray experiments. Stat Appl Genet Mol Biol. 2004;3:Article 3. 10.2202/1544-6115.1027 16646809

[42] Cheadle C, Vawter MP, Freed WJ, Becker KG. Analysis of microarray data using Z score transformation. J Mol Diagn. 2003;5:73–81. 10.1016/S1525-1578(10)60455-2 12707371 PMC1907322

[43] Gossen M, Bujard H. Tight control of gene expression in mammalian cells by tetracycline-responsive promoters. Proc Natl Acad Sci USA. 1992;89:5547–51. 1319065 10.1073/pnas.89.12.5547 PMC49329

[44] Imhof MO, Chatellard P, Mermod N. A regulatory network for the efficient control of transgene expression. J Gene Med. 2000;2:107–16. 10.1002/(SICI)1521-2254(200003/04)2:2<107::AID-JGM91>3.0.CO;2-E 10809144

[45] Winzen R, Kumar Thakur BK, Dittrich-Breiholz O, Shah M, Redich N, Dhamija S, et al Functional analysis of KSRP interaction with the AU-rich element of interleukin-8 and identification of inflammatory mRNA targets. Mol Cell Biol. 2007;27:8388–400. 10.1128/MCB.01493-07 17908789 PMC2169186

[46] Krowczynska AM, Coutts M, Makrides S, Brawerman G. The mouse homologue of the human acidic ribosomal phosphoprotein P0: a highly conserved polypeptide that is under translational control. Nucleic Acids Res. 1989;17:6408 2771657 10.1093/nar/17.15.6408 PMC318307

[47] Eddy SR, Durbin R. RNA sequence analysis using covariance models. Nucleic Acids Res. 1994;22:2079–88. 8029015 10.1093/nar/22.11.2079 PMC308124

[48] Pearson WR. Effective protein sequence comparison. Methods Enzymol 1996;266:227–58. 8743688 10.1016/s0076-6879(96)66017-0

[49] Dogar AM. The role of AUF1 in AU-rich interleukin-6 mRNA regulation. PhD Thesis. University of Lausanne, Switzerland; 2007. p. 1–94.

[50] Mazan-Mamczarz K, Kuwano Y, Zhan M, White EJ, Martindale JL, Lal A, et al Identification of a signature motif in target mRNAs of RNA-binding protein AUF1. Nucleic Acids Res. 2009;37:204–14. 10.1093/nar/gkn929 19033365 PMC2615618

[51] DennisGJ, ShermanBT, HosackDA, YangJ, GaoW, LaneHC, et al DAVID: Database for annotation, visualization, and integrated discovery. Genome Biol. 2003;4:R60.12734009

[52] Brondyk B. pCI and pSI mammalian expression vectors. Promega Notes Magazine. 1994;49:7–11.

[53] Matsushita K, Takeuchi O, Standley DM, Kumagai Y, Kawagoe T, Miyake T, et al Zc3h12a is an RNase essential for controlling immune responses by regulating mRNA decay. Nature. 2009;458:1185–90. 10.1038/nature07924 19322177

[54] Xu J, Peng W, Sun Y, Wang X, Xu Y, Li X, et al Structural study of MCPIP1 N-terminal conserved domain reveals a PIN-like RNase. Nucleic Acids Res. 2012;40:6957–65. 10.1093/nar/gks359 22561375 PMC3413151

[55] Mizgalska D, Wegrzyn P, Murzyn K, Kasza A, Koj A, Jura J, et al Interleukin-1-inducible MCPIP protein has structural and functional properties of RNase and participates in degradation of IL-1® mRNA. FEBS J. 2009;276:7386–99. 10.1111/j.1742-4658.2009.07452.x 19909337

[56] Li M, Cao W, Liu H, Zhang W, Liu X, Cai Z, et al MCPIP1 down-regulates IL-2 expression through an ARE-independent pathway. PLoS One. 2012;7:e49841 10.1371/journal.pone.0049841 23185455 PMC3504106

[57] Uehata T, Iwasaki H, Vandenbon A, Matsushita K, Hernandez-Cuellar E, Kuniyoshi K, et al Malt1-induced cleavage of Regnase-1 in CD4(+) helper T cells regulates immune activation. Cell. 2013;153 1036–49. 10.1016/j.cell.2013.04.034 23706741

[58] Stoecklin G, Tenenbaum SA, Mayo T, Chittur SV, George AD, Baroni TE, et al Genome-wide analysis identifies interleukin-10 mRNA as target of tristetraprolin. J Biol Chem. 2008;283:11689–99. 10.1074/jbc.M709657200 18256032 PMC2431067

[59] Hirata H, Yoshiura S, Ohtsuka T, Bessho Y, Harada T, Yoshikawa K, et al Oscillatory expression of the bHLH factor Hes1 regulated by a negative feedback loop. Science. 2002;298:840–3. 10.1126/science.1074560 12399594

[60] Shimojo H, Ohtsuka T, Kageyama R. Oscillations in notch signaling regulate maintenance of neural progenitors. Neuron. 2008;58:52–64 10.1016/j.neuron.2008.02.014 18400163

[61] Bonev B, Stanley P, Papalopulu N. MicroRNA-9 modulates Hes1 ultradian oscillations by forming a double-negative feedback loop. Cell Reports. 2012;2:10–8 10.1016/j.celrep.2012.05.017 22840391 PMC4103481

[62] Tan S, Ohtsuka T, Gonzalez A, Kageyama R. MicroRNA9 regulates neural stem cell differentiation by controlling Hes1 expression dynamics in the developing brain. Genes Cells. 2012;17:952–61 10.1111/gtc.12009 23134481

[63] Bonni A, Greenberg ME, Schiavi SC. Multiple elements in the c-fos protein-coding region facilitate mRNA deadenylation and decay by a mechanism coupled to translation. Cell. 1994;77:713–25. 8106384

[64] Herrick DJ, Ross J. The half-life of c-myc mRNA in growing and serum-stimulated cells: influence of the coding and 3' untranslated regions and role of ribosome translocation. Mol Cell Biol. 1994;14:2119–28. 8114742 10.1128/mcb.14.3.2119-2128.1994 PMC358572

[65] Kartsogiannis V, Zhou H, Horwood N, Thomas R, Hards D, Quinn J, et al Localization of RANKL (Receptor activator of NF kappa B ligand) mRNA and protein in skeletal and extraskeletal tissues. Bone. 1999;25:525–34 10574572 10.1016/s8756-3282(99)00214-8

[66] Tanos T, Sflomos G, Echeverria P, Ayyanan A, Gutierrez M, Delaloye J, et al Progesterone/RANKL is a major regulatory axis in the human breast. Sci Transl Med. 2013;5:182ra55 10.1126/scitranslmed.3005654 23616122

[67] Polo J, Dell'Oso T, Ranuncolo S, Cerchietti L, Beck D, Da Silva G, et al Specific peptide interference reveals BCL6 transcriptional and oncogenic mechanisms in B-cell lymphoma cells. Nat Med. 2004;10:1329–35 10.1038/nm1134 15531890

[68] Ye B, Chaganti S, Chang C, Niu H, Corradini P, Chaganti R, et al Chromosomal translocations cause deregulated BCL6 expression by promoter substitution in B cell lymphoma. EMBO J. 1995;14:6209–17 8557040 10.1002/j.1460-2075.1995.tb00311.x PMC394745

[69] Chen CY, Shyu AB. Selective degradation of early-response-gene mRNAs: functional analyses of sequence features of the AU-rich elements. Mol Cell Biol. 1994;14:8471–82. 7969180 10.1128/mcb.14.12.8471-8482.1994 PMC359386

[70] Behrens G, Winzen R, Rehage N, Dörrie A, Barsch M, Hoffmann A, et al A translational silencing function of MCPIP1/Regnase-1 specified by the target site context. Nucleic Acids Res. 2018;46:4256–70. 10.1093/nar/gky106 29471506 PMC5934641

[71] Kühn LC. Iron regulatory proteins and their role in controlling iron metabolism. Metallomics. 2014;7:232–43. 10.1039/c4mt00164h 25306858

[72] Chen CY, Gherzi R, Ong SE, Chan EL, Raijmakers R, Pruijn GJ, et al AU binding proteins recruit the exosome to degrade ARE-containing mRNAs. Cell. 2001;107:451–64. 11719186 10.1016/s0092-8674(01)00578-5

[73] Torrisani J, Unterberger A, Tendulkar SR, Shikimi K, Szyf M. AUF1 cell cycle variations define genomic DNA methylation by regulation of DNMT1 mRNA stability. Mol Cell Biol. 2007;27:395–410 10.1128/MCB.01236-06 17030625 PMC1800664

[74] Sun Y, Varambally S, Maher C, Cao Q, Chockley P, Toubai T, et al Targeting of microRNA-142-3p in dendritic cells regulates endotoxin-induced mortality Blood. 2011;117:6172–83 10.1182/blood-2010-12-325647 21474672 PMC3122940

