# Supplementary material for: Short-lived AUF1 p42-binding mRNAs of RANKL and BCL6 have two distinct instability elements each
Source: PLoS One. 2018 Nov 12;13(11):e0206823. doi: 10.1371/journal.pone.0206823 (PMC6231638; doi:10.1371/journal.pone.0206823)
Supplement: S1 Methods — (DOC) [file pone.0206823.s001.doc]

**S1 Methods. Details of plasmids for mRNA half-life measurements by the Tet-off system.**

**pBHTTA.** A constitutive expression vector for the tTA transcription factor was constructed**.** pBIBCTTA was obtained by digesting pBabe-bleo with *Sal*I and *Nco*I to remove the SV40 promoter and ligating it simultaneously to a *Bam*HI-*Xho*I fragment of pUHD15-1 with the tTA transcription factor and a *Nco*I-*Bam*HI fragment with the IRES of pSINCMVIRESGFPNLSWTTV (gift from O. Hagenbüchle). To derive pBHTTA, tTA from pBIBCTTA was ligated as an *Eco*RI-fragment to pBabe-hygro digested with *Eco*RI.

**pZPCTHI.** The reporter vector pZPCTHI was constructed by a series of seven ligations as follows:

(1) pBPTCG was derived from the retroviral based vector pBPSTluc1 with the tetracycline regulatory sequences by digesting with *Bam*HI and *Eco*RI to remove the tTA expression cassette and luciferase reporter. It was then ligated simultaneously to the enhanced green fluorescent protein (EGFP) coding region of pEGFP-N1 (Clontech) digested with *Bam*HI and *Not*I, and to a 313-bp fragment with the bovine growth hormone polyadenylation signal obtained from pCDNA3 (Invitrogen, Carlsbad, CA) by PCR using the forward primer TGACTGTCAGGGCCCAATACGACTCACTATAGGGTGATCTAAGAATTCGCGGCCGCCCTATTCTATAGTGTCACCTAAATGCTAGA and reverse primer ACTGACTATCAATTGAGATCTGCATCCCCAGCATGCCT, digested with *Psp*OMI and *Mfe*I, compatible with *Not*I and *Eco*RI sites. This modifies the original pBPSTluc1 vector by removing the tTA expression cassette and by replacing the luciferase with an EGFP reporter followed by region with multiple cloning sites and stop codons in all three reading frames, as well as a strong polyadenylation signal at 188 bases 3’ from the EGFP stop codon.

(2) In order to derive pBPTHG, pBPTCG was digested with *Xho*I, blunt-ended by Klenow fill-in, redigested with *Bam*HI and then ligated to a fragment from 5x GTTI-GAL4-VV (gift of M. Imhof) that was cut by *Asp718*I, blunt-ended by Klenow fill-in and cut by *Bam*HI. This vector resembles 5x GTT-GAL4-VP16 of Imhof et al. and the fragment used comprises the 7x tet-operator enhancer element of Gossen and Bujard fused to a heterologous minimal promoter composed of an adenovirus major late promoter TATA box and an initiator element of the murine terminal deoxynucleotidyl transferase promoter . pBPTHG substitutes the heterologous minimal promoter for the original CMV minimal promoter .

(3) A self-inactivating (SIN) retrovirus-derivative pSBPTHG of pBPTHG was obtained by deletion of the U3 direct repeats enhancer region in the 3’LTR . pBPTHG was digested partially with *Xba*I, blunt-ended by Klenow fill-in at the 3’LTR site and redigested with *Bam*HI completely to remove the 3’LTR U3 enhancer and then ligated to an enhancer-less 3’LTR fragment obtained by digesting pBPTHG with *Bam*HI and *Pvu*II.

(4) In order to delete the puromycin resistance gene, pSBPTHG was digested partially with *Bsr*GI in the GAG region and completely with *Bgl*II and ligated to a *Bam*HI**-***Bsr*GI fragment from the GAG region of pBabe-puro to obtain pSBTHG.

(5) pSPCTHG was derived from pSBTHG by digesting it with *Xho*I and *Nhe*I to remove the tetracycline-responsive enhancer. It was then ligated simultaneously to a *Hind*III-*Nhe*I fragment of pBabe-puro with the puromycin resistance gene and an *Xho*I-*Hind*III fragment from the tetracycline-responsive enhancer/CMV minimal promoter fusion of pUHC13-3 . Prior to this step, an extra ATG start codon had been deleted from pUHC13-3 by *Hind*III-*Sal*I digestion and blunt-end ligation. The pSPCTHG construct reintroduces the CMV minimal promoter in order to create a bidirectional tetracycline-responsive promoter for the expression of the resistance gene as well as the GFP reporter.

(6) pZPCTHG was derived from pSPCTHG by partially digesting with *Eco*RI, followed by Klenow fill-in and blunt-end ligation to remove the *Eco*RI-site after the CMV minimal promoter, while leaving the *Eco*RI site in the multiple cloning site region intact.

(7) pZPCTHI was derived from pZPCTHG by the insertion of a 133 bp chimeric intron immediately upstream of the EGFP coding region. The intron sequence was amplified from plasmid pCI-neo (Promega, Madison, WI) with the forward primer CACACCGGTATCCACACTTCATCTAGGCACTGGGCAGGTAAG and reverse primer GTGCCATGGTGGCAATTGGGAGTGGACACCTGTG**.** The amplified 199-bp fragment was cut with *Age*I and *Nco*I and inserted into corresponding sites in pZPCTHG. The advantage of pZPCTHI is a potentially higher expression of EGFP hybrid constructs and lower RT-PCR background with a PCR forward primer over the intron-junction.

pZPCTHI_no_stop, lacking stop codons was constructed in order to test RNA instability elements of coding regions in frame behind the EGFP-coding region. ZPCTHI_no_stop was derived from pZPCTHI by site-directed mutagenesis with appropriate PCR primers to replace the initial sequence GAGCTGTACAAGTAAAGCGGCCCAATACGACTCA-CTATAGGGTGATCTAAGAATTCGCGGCCGC at the end of the EGFP coding region with the shorter sequence GAGATCTACAAGAATTCAGCGGCCGC. It removes the natural EPFP stop codon and 3 downstream stop-codons, but maintains the *Eco*RI and *Not*I sites and adds a new *Bgl*II site.

**References**

Brondyk, B. (1994). pCI and pSI mammalian expression vectors. Promega Notes Magazine *49*, 7-11.

Gossen, M., and Bujard, H. (1992). Tight control of gene expression in mammalian cells by tetracycline-responsive promoters. Proc Natl Acad Sci USA *89*, 5547-5551.

Imhof, M.O., Chatellard, P., and Mermod, N. (2000). A regulatory network for the efficient control of transgene expression. J Gene Med *2*, 107-116.

Morgenstern, J.P., and Land, H. (1990). Advanced mammalian gene transfer: high titer retroviral vectors with multiple-drug selection markers and a complementary helper-free packaging cell line. Nucleic Acids Res *18*, 3587-3596.

Paulus, W., Baur, I., Boyce, F.M., Breakefield, X.O., and Reeves, S.A. (1996). Self-contained, tetracycline-regulated retroviral vector system for gene delivery to mammalian cells. J Virol *70*, 62-67.

Smale, S.T., and Baltimore, D. (1989). The "initiator" as a transcription control element. Cell *57*, 103-113.

Soriano, P., Friedrich, G., and Lawinger, P. (1991). Promoter interactions in retrovirus vectors introduced into fibroblasts and embryonic stem cells. J Virol *65*, 2314-2319.
