# Supplementary figures and images for: Short-lived AUF1 p42-binding mRNAs of RANKL and BCL6 have two distinct instability elements each
Source: PLoS One. 2018 Nov 12;13(11):e0206823. doi: 10.1371/journal.pone.0206823 (PMC6231638; doi:10.1371/journal.pone.0206823)

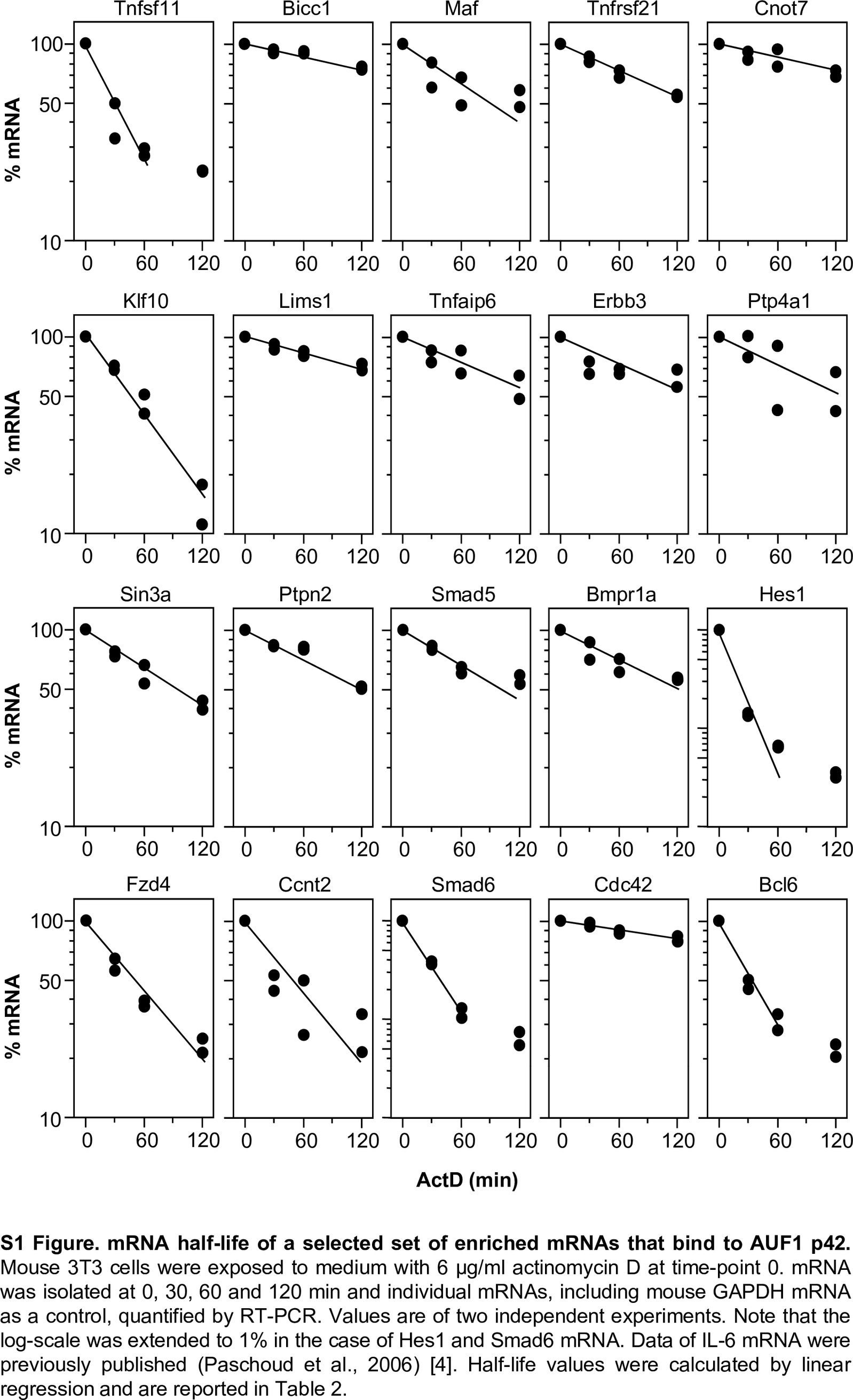

Supplement: S1 Fig — Mouse 3T3 cells were exposed to medium with 6 μg/ml actinomycin D at time-point 0. mRNA was isolated at 0, 30, 60 and 120 min and individual mRNAs, including mouse GAPDH mRNA as a control, quantified by RT-PCR. Values are of two independent experiments. Note that the log-scale was extended to 1% in the case of Hes1 and Smad6 mRNA. Data of IL-6 mRNA were previously published [4]. Half-life values were calculated by linear regression and are reported in Table 2. (TIF) [file pone.0206823.s002.tif]

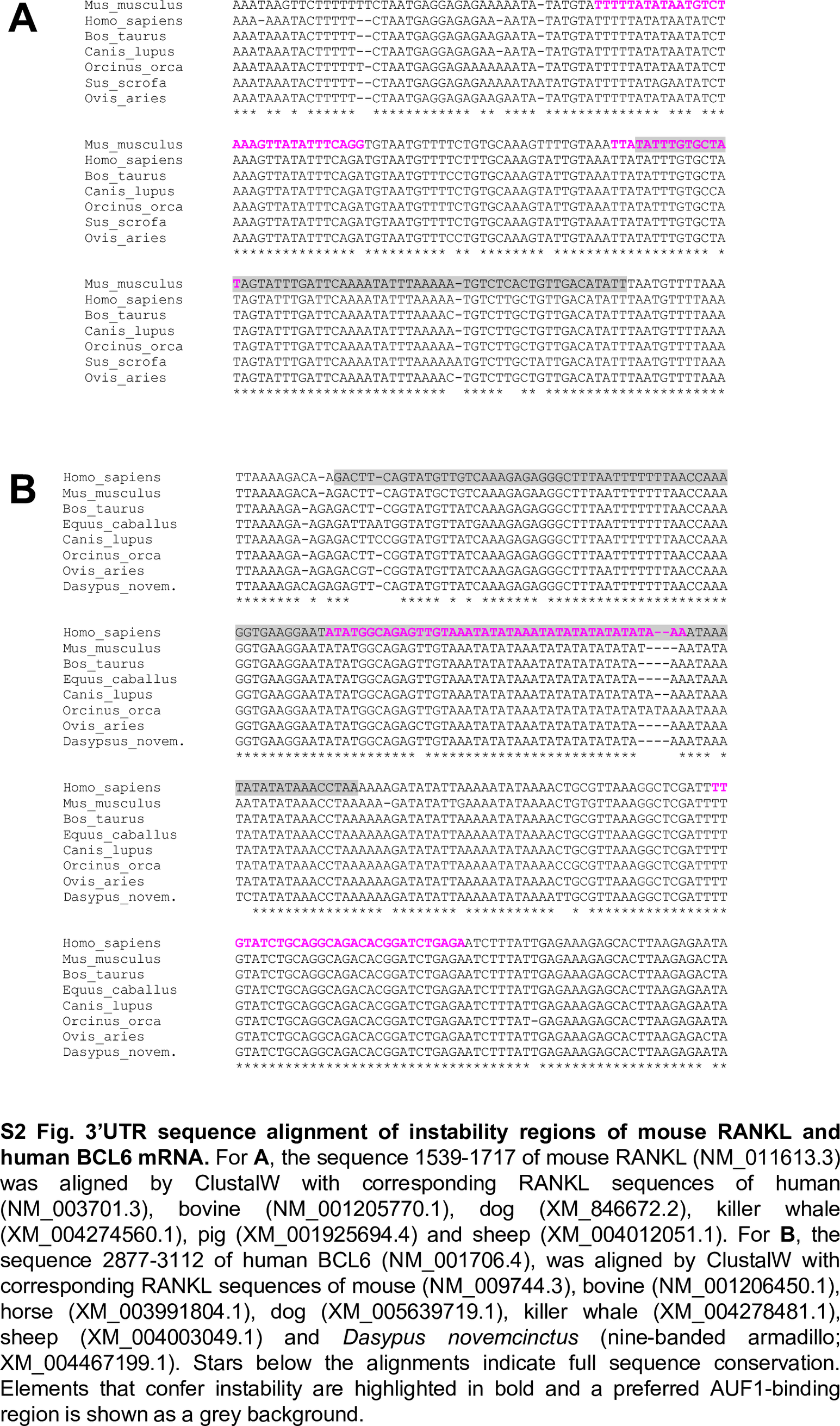

Supplement: S2 Fig — For A, the sequence 1539–1717 of mouse RANKL (NM_011613.3) was aligned by ClustalW with corresponding RANKL sequences of human (NM_003701.3), bovine (NM_001205770.1), dog (XM_846672.2), killer whale (XM_004274560.1), pig (XM_001925694.4) and sheep (XM_004012051.1). For B, the sequence 2877–3112 of human BCL6 (NM_001706.4), was aligned by ClustalW with corresponding RANKL sequences of mouse (NM_009744.3), bovine (NM_001206450.1), horse (XM_003991804.1), dog (XM_005639719.1), killer whale (XM_004278481.1), sheep (XM_004003049.1) and Dasypus novemcinctus (nine-banded armadillo; XM_004467199.1). Stars below the alignments indicate full sequence conservation. Elements that confer instability are highlighted in bold and a preferred AUF1-binding region is shown as a grey background. (TIF) [file pone.0206823.s003.tif]
