# Supplementary material for: Short-lived AUF1 p42-binding mRNAs of RANKL and BCL6 have two distinct instability elements each
Source: PLoS One. 2018 Nov 12;13(11):e0206823. doi: 10.1371/journal.pone.0206823 (PMC6231638; doi:10.1371/journal.pone.0206823)
Supplement: S1 Table — Numbering is based on NCBI reference sequences. Bold letters indicate restriction sites used for cloning. When the natural polyadenylation site is absent, a site in the vector is used for poladenylation. (PDF) [file pone.0206823.s004.pdf]

S1 Table. Primers used for 3'UTR amplification and subcloning into pZPCTHI. Numbering is based on NCBI reference sequences. Bold letters indicate restriction sites used for cloning. When the natural polyadenylation site is absent, a site in the vector is used for polyadenylation.

Construct	NCBI mRNA seq	Insert	Forward primer	Reverse primer	Natural polyadenylation site present
**Construct with mouse 3'UTR**					
GFP-mRANKL	NM_011613.3	1106-2235	CCG**GAATTC**GACTCATTTCGTGGAACATT	CTT**GCGGCCGC**AGGTTTTCGTACAAATTTATTT	yes
**Constructs with human 3'UTRs**					
GFP-RANKL	NM_003701.3	1329-2165 (missing 1111-1328)	CCG**GAATTC**TATCCATGCTCTTGACCTTG	AA**GCGGCCGC**TTTGTACCAAAAAATTAACATTC	no
GFP-KLF10	NM_005655.3	1744-3082	CCG**GAATTC**TGACAGACCGGAAAGTGAAG	AA**GCGGCCGC**AATTCTTTATTGTAAACAAGATAT	yes
GFP-IL6	NM_000600.3	752-979	CCG**GAATTC**TAGCATGGGCACCTCAG	CTT**GCGGCCGC**TAAAATGTTTCAAGTGGTACT	no
GFP-HES1	NM_005524.3	1086-1427	CCG**GAATTC**GGCTCAGGCCACCCCTC	AA**GCGGCCGC**TCAAATAAACTTCCCCAAAGG	no
GFP-SMAD6	NM_005585.4	2395-2860	CCG**GAATTC**CCTCAACAACCCCAGATAG	AA**GCGGCCGC**GAATACTTTATTATCGAGTG	yes
GFP-BCL6	NM_001706.4	2484-3529	CCG**GAATTC**TGAAGCATGGAGTGTTGATG	AA**GCGGCCGC**AGCTATATTTTACAACGCG	no
GFP-HES1CDS-UTR	NM_005524.3	240-1427 in frame	GAC**AGATCT**ACATGCCAGCTGATATAATGGA	AA**GCGGCCGC**TCAAATAAACTTCCCCAAAGG	no
