# Supplementary material for: Short-lived AUF1 p42-binding mRNAs of RANKL and BCL6 have two distinct instability elements each
Source: PLoS One. 2018 Nov 12;13(11):e0206823. doi: 10.1371/journal.pone.0206823 (PMC6231638; doi:10.1371/journal.pone.0206823)
Supplement: S2 Table — Numbering is based on NCBI reference sequence NM_011613.3. Bold letters indicate restriction sites used for cloning or linker sequence in scanning mutants. (PDF) [file pone.0206823.s005.pdf]

S2 Table. Primers used for 3'UTR deletion mutants of mouse RANKL. Numbering is based on NCBI reference sequence NM_011613.3. Bold letters indicate restriction sites used for cloning or linker sequence in scanning mutants.

**Long deletions**			
**Construct**	**Region present**	**Forward primer**	**Reverse primer**
**RANKL.1**	1106-2235	CCG**GAATTC**GACTCATTTCGTGGAACATT	CTT**GCGGCCGC**AGGTTTTCGTACAAATTTATTT
**RANKL.2**	1290-2235	CAA**GAATTC**AGATCTCATGGTGATTACACAACGG	CTT**GCGGCCGC**AGGTTTTCGTACAAATTTATTT
**RANKL.3**	1528-2235	CCG**AGATCT**GGGACCTGCAAATAAGT	CTT**GCGGCCGC**AGGTTTTCGTACAAATTTATTT
**RANKL.4**	1781-2235	CCG**AGATCT**AGAATACTGTTTCTGGTGAC	CTT**GCGGCCGC**AGGTTTTCGTACAAATTTATTT
**RANKL.5**	1991-2235	CCG**AGATCT**TAATAGGCTACTGAAATCTG	CAA**GAATTC**CAAAGGTGAAGGAATATATGG
**RANKL.6**	1290-1530	CAA**GAATTC**CATGGTGATTACACAACGG	GCC**AGATCT**CAGCGCAATGTAACAATTCA
**RANKL.7**	1290-1775	CAA**GAATTC**CATGGTGATTACACAACGG	GCC**AGATCT**TAGCTACGAGTACCTTC
**RANKL.8**	1290-1988	CAA**GAATTC**CATGGTGATTACACAACGG	TAA**GCGGCCGC**AGATCTATCCTTTTTGGCTATGTCAG
**RANKL.9**	1290-1775/1991-2235	CAA**GAATTC**CATGGTGATTACACAACGG	GCC**AGATCT**TAGCTACGAGTACCTTC
**RANKL.10**	1290-1530/1781-2235	CAA**GAATTC**CATGGTGATTACACAACGG	GCC**AGATCT**CAGCGCAATGTAACAATTCA
**RANKL.11**	1425-1988	CAA**GAATTC**CCTGGACATGTGCCACTG	TAA**GCGGCCGC**AGATCTATCCTTTTTGGCTATGTCAG
**RANKL.12**	1528-1988	CAA**GAATTC**TGGGACCTGCAAATAAGT	TAA**GCGGCCGC**AGATCTATCCTTTTTGGCTATGTCAG
**RANKL.13**	1425-1775/1991-2235	CAA**GAATTC**CCTGGACATGTGCCACTG	CTT**GCGGCCGC**AGGTTTTCGTACAAATTTATTT
**RANKL.14**	1528-1775/1991-2235	CAA**GAATTC**TGGGACCTGCAAATAAGT	CTT**GCGGCCGC**AGGTTTTCGTACAAATTTATTT
**Short deletions**			
**For constructs RANKL.15-21**		**5' fragment forward primer**	**3' fragment reverse primer**
		CAA**GAATTC**CCTGGACATGTGCCACTG	TAA**GCGGCCGC**TATCCTTTTTGGCTATGTCAG
**Construct**	**Region present**	**5' fragment reverse primer**	**3' fragment forward primer**
**RANKL.15**	1425-1527/1578-1988	AAAAATACA**AGATCT**CGCAATGTAACAATTCAAAAG	CATTGCG**AGATCT**TGTATTTTTATATAATGTCTAAAG
**RANKL.16**	1425-1577/1628-1988	CTTTGCA**AGATCT**TATATTTTTCTCTCCTCATTAG	GAAAAATATA**AGATCT**TGCAAAGTTTTGTAAATTATATTT
**RANKL.17**	1425-1627/1690-1988	GTGAGAC**AGATCT**CAGAAAACATTACACCTGAAA	CTG**AGATCT**CACTGTTGACATATTTAATGTT
**RANKL.18**	1425-1685/1734-1988	CACCAG**AGATCT**ATTTTTAAATATTTTGAATCAAATAC	ATTTAAAAAT**AGATCT**CTGGTGCACTTTGTAATTCC
**RANKL.19**	1425-1733/1792-1988	CACCAG**AGATCT**TTAAATACATCTGTACATTTAAAA	TGTATTTAA**AGATCT**CTGGTGACCACATGTAGTT
**RANKL.20**	1425-1792/1855-1988	TAGTTTT**GAGATC**TAAACAGTATTCTGCCCCCT	TACTGTTT**AGATCT**CAAAACTATGCAAGCAAAATAA
**RANKL.21**	1425-1854/1923-1988	GGTGAC**AGATCT**ACAAGTCTGAAGACTCTATTA	GACTTGT**AGATCT**GTCACCAGGTGCCTTTCA
		**Forward Primer**	**Reverse Primer**
**RANKL.22**	1425-1922	CAA**GAATTC**CCTGGACATGTGCCACTG	TAA**GCGGCCGC**CAACATCCTACTTATTATTCAA
**15-base scanning mutants**			
**For constructs RANKL 23-30**		**5' fragment forward primer**	**3' fragment reverse primer**
	1425-1988	CAA**GAATTC**CCTGGACATGTGCCACTG	TAA**GCGGCCGC**TATCCTTTTTGGCTATGTCAG
**Construct**	**Sequence mutated**	**5' fragment reverse primer**	**3' fragment forward primer**
**RANKL.23**	1569-1583	AAA**GAGCTACGCGTTGCT**CTCTCCTCATTAGAAAAAAAG	G**AGCAACGCGTAGCT**CTTTATATAATGTCTAAAGTTATATTTC
**RANKL.24**	1584-1598	TT**GAGCTACGCGTTGCT**AATACATATATTTTTCTCTCCTC	ATT**AGCAACGCGTAGCT**CAAGTTATATTTCAGGTGTAATGT
**RANKL.25**	1599-1613	CA**GAGCTACGCGTTGCT**TAGACATTATATAAAAATACATATATT	TA**AGCAACGCGTAGCT**CTGTAATGTTTTCTGTGCAAAGTT
**RANKL.26**	1614-1628	GC**GAGCTACGCGTTGCT**CCTGAAATATAACTTTAGACAT	G**AGCAACGCGTAGCT**CGCAAAGTTTTGTAAATTATATTTGTG
**RANKL.27**	1629-1643	AA**GAGCTACGCGTTGCT**ACAGAAAACATTACACCTGAA	GT**AGCAACGCGTAGCTCT**TATATTTGTGCTATAGTATTTG
**RANKL.28**	1644-1658	CT**GAGCTACGCGTTGCT**TTTACAAAACTTTGCACAGAAAA	AAA**AGCAACGCGTAGCT**CAGTATTTGATTCAAAATATTTAAAAAT
**RANKL.29**	1659-1673	AT**GAGCTACGCGTTGCT**ATAGCACAAATATAATTTACAAAAC	TAT**AGCAACGCGTAGCT**CATATTTAAAAATGTCTCACTGTT
**RANKL.30**	1674-1688	GA**GAGCTACGCGTTGCT**TTTGAATCAAATACTATAGCAC	AA**AGCAACGCGTAGCTCT**CACTGTTGACATATTTAATGT
