# Supplementary material for: Short-lived AUF1 p42-binding mRNAs of RANKL and BCL6 have two distinct instability elements each
Source: PLoS One. 2018 Nov 12;13(11):e0206823. doi: 10.1371/journal.pone.0206823 (PMC6231638; doi:10.1371/journal.pone.0206823)
Supplement: S3 Table — Numbering is based on NCBI reference sequence NM_001706.4. Bold letters indicate restriction sites used for cloning or linker sequence in scanning mutants. (PDF) [file pone.0206823.s006.pdf]

S3 Table. Primers used for amplification of 3'UTR fragments of human BCL6. Numbering is based on NCBI reference sequence NM_001706.4. Bold letters indicate restriction sites used for cloning or linker sequence in scanning mutants.

**Long deletions**			
**Construct**	**Region present**	**Forward primer**	**Reverse primer**
**BCL6.1**	2484-3529	CCG**GAATTC**TGAAGCATGGAGTGTTGATG	TAA**GCGGCCGC**AGCTATATTTTACAACGCG
**BCL6.2**	2484-2746	CCG**GAATTC**TGAAGCATGGAGTGTTGATG	TAA**GCGGCCGC**AGATCTTTGCTGACATGGTTCACC
**BCL6.3**	2734-3048	CAA**GAATTC**ATTTTATATGTCAAAGCAGG	TAA**GCGGCCGC**AGATCTCGAGCCTTTAACGCAGTT
**BCL6.4**	3030-3265	CAA**GAATTC**AGATCTAACTGCGTTAAAGGCTCGAT	TAA**GCGGCCGC**TACACATTTTTCCTTCTGCAG
**BCL6.5**	3191-3529	CAA**GAATTC**AGATCTAAGGTTTACAATTTACAAAGTG	TAA**GCGGCCGC**AGCTATATTTTACAACGCG
**BCL6.6**	2484-3048	CCG**GAATTC**TGAAGCATGGAGTGTTGATG	TAA**GCGGCCGC**AGATCTCGAGCCTTTAACGCAGTT
**BCL6.7**	2734-3265	CAA**GAATTC**ATTTTATATGTCAAAGCAGG	TAA**GCGGCCGC**TACACATTTTTCCTTCTGCAG
**BCL6.8**	3030-3529	CAA**GAATTC**AGATCTAACTGCGTTAAAGGCTCGAT	TAA**GCGGCCGC**AGCTATATTTTACAACGCG
**BCL6.9**	2483-3265	CCG**GAATTC**TGAAGCATGGAGTGTTGATG	TAA**GCGGCCGC**TACACATTTTTCCTTCTGCAG
**BCL6.10**	2734-3529	CAA**GAATTC**ATTTTATATGTCAAAGCAGG	TAA**GCGGCCGC**AGCTATATTTTACAACGCG
**For constructs BCL6.11-12**		**5' fragment forward primer**	**3' fragment reverse primer**
		CCG**GAATTC**TGAAGCATGGAGTGTTGATG	TAA**GCGGCCGC**AGCTATATTTTACAACGCG
		**5' fragment reverse primer**	**3' fragment forward primer**
**BCL6.11**	2484-2746/3030-3529	TAA**GCGGCCGC**AGATCTTTGCTGACATGGTTCACC	CAA**GAATTC**AGATCTAACTGCGTTAAAGGCTCGAT
**BCL6.12**	2483-3048/3191-3529	TAA**GCGGCCGC**AGATCTCGAGCCTTTAACGCAGTT	CAA**GAATTC**AGATCTAAGGTTTACAATTTACAAAGTG
**Short deletions**			
**Construct**	**Region present**	**Forward Primer**	**Reverse Primer**
**BCL6.13**	2825-3265	CAA**GAATTC**GTATATGTTTTGTGGGAACAG	TAA**GCGGCCGC**TACACATTTTTCCTTCTGCAG
**For constructs BCL6.13-19**		**5' fragment forward primer**	**3' fragment reverse primer**
		CAA**GAATTC**ATTTTATATGTCAAAGCAGG	TAA**GCGGCCGC**TACACATTTTTCCTTCTGCAG
**Construct**	**Region present**	**5' fragment reverse primer**	**3' fragment forward primer**
**BCL6.14**	2734-2824/2887-3265	TGAAGTC**AGATCT**ATTCCTCATTTTGCAGACTAA	GAGGAAT**AGATCT**GACTTCAGTATGTTGTCAAA
**BCL6.15**	2734-2886/2951-3265	TGAAGTC**AGATCT**TTGTCTTTTAAAAGAATGCACA	AAAGACAA**AGATCT**<u>GCAGAG</u>TTGTAAATATATAAATA
**BCL6.16**	2734-2950/3012-3265	TAATATATC**AGATCT**CATATATTCCTTCACCTTTGG	GAATATATG**AGATCT**GATATATTAAAAATATAAAACTGC
**BCL6.17**	2734-3011/3063-3265	GTCTGC**AGATCT**TTTGTTAGGTTTATATATATTTATT	CTAACAAA**AGATCT**GCAGACACGGATCTGAGA
**BCL6.18**	2734-3062/3120-3265	ATGCAATA**AGATCT**CTGCAGATACAAAATCGAGC	CTGCAG**AGATCT**TATTGCATCTGTATAAGTAAGA
**BCL6.19**	2734-3119/3191-3265	TAAACCTT**AGATCT**CTTAAAATATTCTCTTAAGTGC	TATTTTAAG**AGATCT**AAGGTTTACAATTTACAAAGTG
**Construct**	**Region present**	**Forward Primer**	**Reverse Primer**
**BCL6.20**	2734-3190	CAA**GAATTC**ATTTTATATGTCAAAGCAGG	TAA**GCGGCCGC**CACTTGCAAAAAAATACAAATAC
**15-base scanning mutants**			
**For constructs BCL6.21-32**		**5' fragment forward primer**	**3' fragment reverse primer**
	2734-3265	CAA**GAATTC**ATTTTATATGTCAAAGCAGG	TAA**GCGGCCGC**TACACATTTTTCCTTCTGCAG
**Construct**	**Region mutated**	**5' fragment reverse primer**	**3' fragment forward primer**
**BCL6.21**	2946-2960	TT**GAGCTACGCGTTGCT**ATTCCTTCACCTTTGGTTAAAA	AAT**AGCAACGCGTAGCTC**AAATATATAAATATATATATATATATA AAATAAATATATATATAAACC
**BCL6.22**	2961-2975	TA**GAGCTACGCGTTGCT**ACAACTCTGCCATATATTCC	GT**AGCAACGCGTAGCTC**TATATATATAAAATAAATATATATAAACC
**BCL6.23**	2976-2990	ATT**GAGCTACGCGTTGCT**TATATTTATATATTTACAACTCTG	TA**AGCAACGCGTAGCTC**AATATATATAAACCTAACAAAGATAT
**BCL6.24**	2991-3005	GTT**GAGCTACGCGTTGCT**TATTTTATATATATATATATATTTATATATTTACTATATATAAACTC	A**AGCAACGCGTAGCTC**AACAAAGATATATTAAAAATATAAAAC
**BCL6.25**	3006-3020	TT**GAGCTACGCGTTGCT**AGGTTTATATATATTTATTTTATATATATTTATATATTTACAAC	CT**AGCAACGCGTAGCTC**AAAATATAAAACTGCGTTAAAGGCTC
**BCL6.26**	3021-3035	AC**GAGCTACGCGTTGCT**TAATATATCTTTTTTAGGTTTATATATATTTATTTT	TA**AGCAACGCGTAGCTC**GTTAAAGGCTCGATTTTGTATC
**BCL6.27**	3036-3050	**GAGCTACGCGTTGCT**GCAGTTTTATATTTTTAATATATCTT	TGC**AGCAACGCGTAGCTC**TTGTATCTGCAGGCAGACAC
**BCL6.28**	3051-3065	TC**GAGCTACGCGTTGCT**AATCGAGCCTTTAACGCAGTTTTATATTTTT	TT**AGCAACGCGTAGCTC**GACACGGATCTGAGAATCTT
**BCL6.29**	3066-3080	GAT**GAGCTACGCGTTGCT**TGCCTGCAGATACAAAATCG	A**AGCAACGCGTAGCTC**ATCTTTATTGAGAAAGAGCAC
**BCL6.30**	3081-3095	CTC**GAGCTACGCGTTGCT**TCTCAGATCCGTGTCTGC	**AGCAACGCGTAGCTC**GAGCACTTAAGAGAATATTTTAA
**BCL6.31**	3096-3110	ATA**GAGCTACGCGTTGCT**TTTCTCAATAAAGATTCTCAGATCCGTG	AA**AGCAACGCGTAGCTC**TATTTTAAGTATTGCATCTGTATA
**BCL6.32**	3111-3125	GAT**GAGCTACGCGTTGCT**TTCTCTTAAGTGCTCTTTCTC	A**AGCAACGCGTAGCTC**ATCTGTATAAGTAAGAAAATATTTTG
