# Supplementary material for: Short-lived AUF1 p42-binding mRNAs of RANKL and BCL6 have two distinct instability elements each
Source: PLoS One. 2018 Nov 12;13(11):e0206823. doi: 10.1371/journal.pone.0206823 (PMC6231638; doi:10.1371/journal.pone.0206823)
Supplement: S4 Table — Numbering is based on NCBI reference sequence NM_011613.3. Bold letters indicate restriction sites used for cloning. (PDF) [file pone.0206823.s007.pdf]

S4 Table. Primers used for amplification of 3'UTR fragments of mouse RANKL. Numbering is based on NCBI reference sequence NM_011613.3. Bold letters indicate restriction sites used for cloning.

**Long inserts**			
**Construct**	**Region present**	**Forward primer**	**Reverse primer**
**RANKL.1-6**	1106-1530	CCG**GAATTC**GACTCATTTCGTGGAACATT	GCC**AGATCT**CAGCGCAATGTAACAATTCA
**RANKL.6/15-16i**	1290-1530/1528-1627	CCG**AGATCT**GGGACCTGCAAATAAGT	TAA**GCGGCCGC**CAGAAAACATTACACCTGAA
**RANKL.6/16-17i**	1290-1530/1578-1685	TGCG**AGATCT**TGTATTTTTATATAATGTCTAAAGTTATA	TAA**GCGGCCGC**ATTTTTAAATATTTTGAATCAAATAC
**RANKL.6/17-18i**	1290-1530/1628-1733	GAAAAATATA**AGATCT**TGCAAAGTTTTGTAAATTATATTT	TAA**GCGGCCGC**TTAAATACATCTGTACATTTAAAA
**RANKL.6/18-19i**	1290-1530/1686-1792	GTTTTCTG**AGATCT**GTCTCACTGTTGACATATTTA	TAA**GCGGCCGC**AAACAGTATTCTGCCCCCT
**RANKL.6/19-20i**	1290-1530/1734-1854	ATTTAAAAAT**AGATCT**CTGGTGCACTTTGTAATTCC	TAA**GCGGCCGC**ACAAGTCTGAAGACTCTATTA
**RANKL.6/20-21i**	1290-1530/1792-1930	TGTATTTAA**AGATCT**CTGGTGACCACATGTAGTT	TAA**GCGGCCGC**CAACATCCTACTTATTATTCAA
**RANKL.6/21-22i**	1290-1530/1855-1988	TACTGTTT**AGATCT**CAAAACTATGCAAGCAAAATAA	TAA**GCGGCCGC**TATCCTTTTTGGCTATGTCAG
**RANKL.6/5A**	1290-1530/1991-2125	CCG**AGATCT**TAATAGGCTACTGAAATCTG	CTT**GCGGCCGC**TTTTTGGCTGATAAACTAT
**RANKL.6/5B**	1290-1530/2105-2235	CCG**AGATCT**ATAGTTTATCAGCCAAAAA	CTT**GCGGCCGC**AGGTTTTCGTACAAATTTATTT
**RANKL.15-16i**	1528-1627	CCG**AGATCT**GGGACCTGCAAATAAGT	TAA**GCGGCCGC**CAGAAAACATTACACCTGAA
**RANKL.16-17i**	1578-1685	TGCG**AGATCT**TGTATTTTTATATAATGTCTAAAGTTATA	TAA**GCGGCCGC**ATTTTTAAATATTTTGAATCAAATAC
**RANKL.17-18i**	1628-1733	GAAAAATATA**AGATCT**TGCAAAGTTTTGTAAATTATATTT	TAA**GCGGCCGC**TTAAATACATCTGTACATTTAAAA
**RANKL.18-19i**	1686-1792	GTTTTCTG**AGATCT**GTCTCACTGTTGACATATTTA	TAA**GCGGCCGC**AAACAGTATTCTGCCCCCT
**RANKL.19-20i**	1734-1854	ATTTAAAAAT**AGATCT**CTGGTGCACTTTGTAATTCC	TAA**GCGGCCGC**ACAAGTCTGAAGACTCTATTA
**RANKL.20-21i**	1792-1930	TGTATTTAA**AGATCT**CTGGTGACCACATGTAGTT	TAA**GCGGCCGC**CAACATCCTACTTATTATTCAA
**RANKL.21-22i**	1855-1988	TACTGTTT**AGATCT**CAAAACTATGCAAGCAAAATAA	TAA**GCGGCCGC**TATCCTTTTTGGCTATGTCAG
**RANKL.5A**	1991-2125	CCG**AGATCT**TAATAGGCTACTGAAATCTG	CTT**GCGGCCGC**TTTTTGGCTGATAAACTAT
**RANKL.5B**	2105-2235	CCG**AGATCT**ATAGTTTATCAGCCAAAAA	CTT**GCGGCCGC**AGGTTTTCGTACAAATTTATTT
**Medium inserts**			
**Construct**	**Region present**	**Forward primer**	**Reverse primer**
**RANKL.15i**	1528-1577	CCG**AGATCT**GGGACCTGCAAATAAGT	TAA**GCGGCCGC**TATATTTTTCTCTCCTCATTAGAAAA
**RANKL.16i**	1578-1627	TGCG**AGATCT**TGTATTTTTATATAATGTCTAAAGTTATA	TAA**GCGGCCGC**CAGAAAACATTACACCTGAA
**RANKL.17i**	1628-1685	GAAAAATATA**AGATCT**TGCAAAGTTTTGTAAATTATATTT	TAA**GCGGCCGC**ATTTTTAAATATTTTGAATCAAATAC
**RANKL.18i**	1686-1733	GTTTTCTG**AGATCT**GTCTCACTGTTGACATATTTA	TAA**GCGGCCGC**TTAAATACATCTGTACATTTAAAA
**RANKL.19i**	1734-1792	ATTTAAAAAT**AGATCT**CTGGTGCACTTTGTAATTCC	TAA**GCGGCCGC**AAACAGTATTCTGCCCCCT
**RANKL.20i**	1793-1854	TGTATTTAA**AGATCT**CTGGTGACCACATGTAGTT	TAA**GCGGCCGC**ACAAGTCTGAAGACTCTATTA
**RANKL.21i**	1855-1922	TACTGTTT**AGATCT**CAAAACTATGCAAGCAAAATAA	TAA**GCGGCCGC**CAACATCCTACTTATTATTCAA
**RANKL.22i**	1923-1988	GACTTGT**AGATCT**GTCACCAGGTGCCTTTCA	TAA**GCGGCCGC**TATCCTTTTTGGCTATGTCAG
