# Supplementary material for: Short-lived AUF1 p42-binding mRNAs of RANKL and BCL6 have two distinct instability elements each
Source: PLoS One. 2018 Nov 12;13(11):e0206823. doi: 10.1371/journal.pone.0206823 (PMC6231638; doi:10.1371/journal.pone.0206823)
Supplement: S5 Table — Numbering is based on NCBI reference sequence NM_001706.4. Bold letters indicate restriction sites used for cloning. (PDF) [file pone.0206823.s008.pdf]

S5 Table. Primers used for amplification of 3'UTR fragments of human BCL6. Numbering is based on NCBI reference sequence NM_001706.4. Bold letters indicate restriction sites used for cloning.

**Long inserts**			
**Construct**	**Region present**	**Forward primer**	**Reverse primer**
**BCL6.2/13-14i**	2484-2746/2754-2887	GA**AGATCT**ATCATTTTATATGTCAAAGCAG	TAA**GCGGCCGC**TTGTCTTTTAAAAGAATGCACA
**BCL6.2/14-15i**	2484-2746/2825-2950	GA**AGATCT**GTATATGTTTTGTGGGACAG	TAA**GCGGCCGC**CATATATTCCTTCACCTTTGG
**BCL6.2/15-16i**	2484-2746/2887-3011	GAGGAAT**AGATCT**GACTTCAGTATGTTGTCAAA	TAA**GCGGCCGC**TTTTTTAGGTTTATATATATTTATT
**BCL6.2/16-17i**	2484-2746/2951-3062	AAAGACAA**AGATCT**GCAGAGTTGTAAATATATAAATA	TAA**GCGGCCGC**CTGCAGATACAAAATCGAGC
**BCL6.2/17-18i**	2484-2746/3012-3119	GAATATATG**AGATCT**GATATATTAAAAATATAAAACTGC	TAA**GCGGCCGC**CTTAAAATATTCTCTTAAGTGC
**BCL6.2/18-19i**	2484-2746/3063-3190	CTAACAAA**AGATCT**GCAGACACGGATCTGAGA	TAA**GCGGCCGC**CACTTGCAAAAAAATACAAATAC
**BCL6.2/19-20i**	2484-2746/3120-3265	CTGCAG**AGATCT**TATTGCATCTGTATAAGTAAGA	TAA**GCGGCCGC**TACACATTTTTCCTTCTGCAG
**Medium inserts**			
**Construct**	**Region present**	**Forward primer**	**Reverse primer**
**BCL6.14i**	2825-2887	GA**AGATCT**GTATATGTTTTGTGGGACAG	TAA**GCGGCCGC**TTGTCTTTTAAAAGAATGCACA
**BCL6.15i**	2887-2950	GAGGAAT**AGATCT**GACTTCAGTATGTTGTCAAAGAGA	TAA**GCGGCCGC**CATATATTCCTTCACCTTTGG
**BCL6.16i**	2951-3011	**GATCT**GCAGAGTTGTAAATATATAAATATATATATAT<br>ATAAAATAAATATATATAAACCTAAAAAAGC	**GGCCGC**TTTTTTAGGTTTATATATATTTATTTTATAT<br>ATATATATATTTAATATTTACAACTCTGCA
**BCL6.17i**	3012-3062	GAATATATG**AGATCT**GATATATTAAAAATATAAAACTGC	TAA**GCGGCCGC**CTGCAGATACAAAATCGAGC
**BCL6.18i**	3063-3119	CTAACAAA**AGATCT**GCAGACACGGATCTGAGA	TAA**GCGGCCGC**CTTAAAATATTCTCTTAAGTGC
**BCL6.19i**	3120-3190	CTGCAG**AGATCT**TATTGCATCTGTATAAGTAAGA	TAA**GCGGCCGC**CACTTGCAAAAAAATACAAATAC
**BCL6.20i**	3191-3265	TATTTTAAG**AGATCT**AAGGTTTACAATTTACAAAGTG	TAA**GCGGCCGC**TACACATTTTTCCTTCTGCAG
**BCL6.5A**	3266-3376	CCG**GAATTC**CATTTTGTTCTAGTTTTCAGTTTGT	TAA**GCGGCCGC**CACTTCAAAAAGGGATGGTG
**BCL6.5B**	3377-3528	CCG**GAATTC**TAGGCAGACACAGGGACTTG	AA**GCGGCCGC**CAGCTATATTTTACAACGCG
