# Supplementary material for: Short-lived AUF1 p42-binding mRNAs of RANKL and BCL6 have two distinct instability elements each
Source: PLoS One. 2018 Nov 12;13(11):e0206823. doi: 10.1371/journal.pone.0206823 (PMC6231638; doi:10.1371/journal.pone.0206823)
Supplement: S6 Table — (PDF) [file pone.0206823.s009.pdf]

S6 Table. Primers used for microarray data confirmation and endogenous half-life measurements by RT-PCR.

Name	Refseq	PCR-amplified	Forward primer	Reverse primer
Tnfsf11=RANKL	NM_011613.3	884-1040	ACCAGCATCAAAATCCCAAG	AAGGGTTGGACACCTGAATG
Bicc1	NM_031397.2	2307-2434	CGGCAGAAACCATTAAGGAA	GACTGCTGGACCTTGAGAGG
Maf	NM_001025577	25-140	CAGGAGGATGGCTTCAGAAC	CCGGTTCCTTTTTCACTTCA
Tnfrsf21	NM_178589.3	1048-1181	GGGACCAAGGAGACAGACAA	CATAGGTGGAGGAAGGGACA
Cnot7	NM_011135.5	496-600	CCAACTGTTGCGGTGTAATG	AACTGCCAAGTTGACGTTCC
Klf10	NM_013692.3	1395-1505	GAAGTTTGCCTGTCCCATGT	ACTTCCATTTGCCAGTTTGG
Lims1	NM_026148.3	3130-3234	CCAAGGGGGAAATCCTGTAT	TGTGTGCATGTGTGTGTGTG
Tnfaip6	NM_009398.2	339-464	CGGATACCCCATTGTGAAAC	TCCTTTGCATGTGGGTTGTA
Erbb3	NM_010153.1	1047-1173	TGGGTTCGTGAACTGTACCA	CCGGACTGTCCTGAAAACAT
Ptp4a1	NM_011200.2	243-370	GTTTCTGAAGGGCAGTGGAG	ACGCCTGTGTCCAGGTTATC
Sin3a	NM_011378.2	3638-3760	GCTCCTGGACACAGAAGAGG	TGTGCCAGATGTTCTCGAAG
Ptpn2	NM_008977.3	133-233	GAGTTCGAGGAACTGGATGC	TCTGGAAACTTGGCCACTCT
Smad5	NM_008541.3	338-446	ATTGTTGGGCTGGAAACAAG	CTCCAGCTCCTCCATAGCAC
Bmpr1a	NM_009758.4	733-832	ATGCAAGGATTCACCGAAAG	AACAACAGGGGGCAGTGTAG
Il-6	NM_031168.1	444-542	AAGCCAGAGTCCTTCAGAGA	GGAAATTGGGGTAGGAAGGA
Hes1	NM_008235.2	1014-1132	TAACGCAGTGTCACCTTCCA	AAGAGAGAGGTGGGCTAGGG
Fzd4	NM_008055.4	630-735	GCCAATGTGCACAGAGAAGA	GGCAAACCCAAATTCTCTCA
Ccnt2	NM_028399.1	1932-2048	ACCACCACTCCAAAATGAGC	GAGGGGGTAAGGGATGGTTA
Smad6	NM_008542.3	1900-2003	ACAAGCCACTGGATCTGTCC	GACATGCTGGCATCTGAGAA
Cdc42	NM_009861.3	732-859	CTGCTATGAACGCATCTCCA	GCAGGGCGTTTGTCATTATT
Bcl6	NM_009744.3	492-592	CACTTAAACCTCCCCGTGAA	TGGCATATTGTTCTCCACGA
RplP0=Arbp	NM_007475.5	540-631	CTTTGGGCATCACCACGAA	GCTGGCTCCCACCTTGTCT
