# Supplementary material for: Short-lived AUF1 p42-binding mRNAs of RANKL and BCL6 have two distinct instability elements each
Source: PLoS One. 2018 Nov 12;13(11):e0206823. doi: 10.1371/journal.pone.0206823 (PMC6231638; doi:10.1371/journal.pone.0206823)
Supplement: S7 Table — The list comprises all mRNAs that were more than 3-fold enriched on microarrays, sorted according to the n-fold enrichment. The ARE score and number of AUUUA elements in the 3'UTR was determined by the program AREScore (Spasic et al., 2012)[38]. The ARED cluster refers to the presence in the ARED Organism database (Halees et al., 2008)[33]. N/A, not available, indicates mRNAs not found in the ARED database. mRNA decay rates known prior to this study in mouse ES cells (a) (Sharova et al., 2009)[37], or for human homologs in lymphocytes (b) (Raghavan et al., 2002)[35] and HepG2 cells (c) (Yang et al., 2003)[36], or known from other studies (d) (Paschoud et al., 2006)[4]. Known half-lives >3h in Sharova et al. (2009)[37] are reported as stable. Original data can be obtained from the authors. (PDF) [file pone.0206823.s010.pdf]

S7 Table. Complete list of mRNAs of mouse NIH-3T3 cells enriched by RNP-IP of myc-AUF1 p42. The list comprises all mRNAs that were more than 3-fold enriched on microarrays, sorted according to the n-fold enrichment. The ARE score and number of AUUUA elements in the 3'UTR was determined by the program ARE*Score* (Spacic et al., 2012)[38]. The ARED cluster refers to the presence in the ARED Organism database (Halees et al., 2008)[33]. N/A, not available, indicates mRNAs not found in the ARED database. mRNA decay rates known prior to this study in mouse ES cells (a) (Sharova et al., 2009)[37], or for human homologs in lymphocytes (b) (Raghavan et al., 2002)[35] and HepG2 cells (c) (Yang et al., 2003)[36], or known from other studies (d) (Paschoud et al., 2006)[4]. Known half-lives >3h in Sharova et al. (2009)[37] are reported as stable. Microarray data are available at NCBI GEO with ID GSE64761.

Gene Symbol	Entrez Gene	Gene Description	Refseq RNA Accession	n-Fold Enrich-ment	ARE Score	3'AUUUA Count	3'UTR Length	ARED Cluster	mRNA Half-life (h)
Tnfsf11	21943	tumor necrosis factor superfamily, member 11 (RANKL)	NM_011613	26.5	7.8	6	1138	Cluster 5	1.5(a)
Mbnl3	171170	muscleblind-like 3 (Drosophila)	NM_134163	26.1	19.9	16	7513	no ARE	stable
Dck	13178	deoxycytidine kinase	NM_007832	21.9	7.5	6	2007	no ARE	stable
Dus4l	71916	dihydrouridine synthase 4-like (S. cerevisiae)	NM_028002	19.6	0	0	621	no ARE	stable
Tmed2	56334	transmembrane emp24 domain trafficking protein 2	NM_019770	19.6	6.95	5	1352	no ARE	stable
Sap18	20220	Sin3-associated polypeptide 18	NM_009119	18.1	6.7	4	2916	Cluster 5	stable
Pcsk6	18553	proprotein convertase subtilisin/kexin type 6	NM_011048	16.9	0	0	1358	N/A	stable
Bicc1	83675	Bicaudal C homolog 1 (Drosophila)	XM_006514341	16.8	12.6	9	2541	N/A	stable
Cisd1	52637	CDGSH iron sulfur domain 1	NM_134007	16.7	1	1	543	no ARE	stable
Cct4	12464	chaperonin subunit 4 (delta)	NM_009837	15.7	1.3	1	185	no ARE	stable
Car13	71934	carbonic anhydrase 13	NM_024495	15.6	4.1	2	1428	no ARE	stable
Rmnd5a	68477	required for meiotic nuclear division 5 hom. A (S. cerev.)	NM_024288	15.4	14.15	11	4485	no ARE	stable
Maf	17132	avian musculoaponeurotic fibrosarcoma (v-maf) homolog	NM_001025577	15.3	4.9	4	2452	no ARE	stable
Tnfrsf21	94185	tumor necrosis factor receptor superfamily, member 21	NM_178589	13.6	2.6	2	1176	no ARE	stable
Cnot7	18983	CCR4-NOT transcription complex, subunit 7	NM_011135	13.5	6.2	5	1449	no ARE	stable
Man2a2	140481	mannosidase 2, alpha 2	NM_172903	12.9	1.3	1	2585	no ARE	stable
Klf10	21847	Kruppel-like factor 10	NM_013692	12.9	4.9	4	1499	no ARE	2.0(a)
Lims1	110829	LIM and senescent cell antigen-like domains 1	NM_026148	12.6	5.2	4	3123	no ARE	stable
Slc16a1	20501	solute carrier family 16, member 1	NM_009196	11.9	13.4	11	2740	Cluster 5	stable
Gatad1	67210	GATA zinc finger domain containing 1	NM_026033	11.4	5.2	4	1427	Cluster 5	stable
Tmem62	96957	transmembrane protein 62	NM_175285	11.2	0	0	717	no ARE	stable
Lpgat1	226856	lysophosphatidylglycerol acyltransferase 1	NM_172266	11.2	16.7	14	5726	no ARE	stable
Dnajb4	67035	DnaJ (Hsp40) homolog, subfamily B, member 4	NM_025926	11.1	5.2	4	1276	Cluster 5	stable
Cmbl	69574	carboxymethylenebutenolidase homolog (Pseudomonas)	NM_181588	11.1	0	0	284	no ARE	stable
Mmd	67468	monocyte to macrophage differentiation-associated	NM_026178	11.0	2.6	2	1680	Cluster 5	stable
Atp6v1a	11964	ATPase, H+ transporting, lysosomal V1 subunit A	NM_007508	11.0	7.8	6	2020	no ARE	stable
Serpinb9	20706	serine (or cysteine) peptidase inhibitor, clade B, memb. 9	NM_009256	10.9	6.2	5	2107	no ARE	stable
Rassf6	73246	Ras association (RalGDS/AF-6) domain family 6	NM_028478	10.6	1.3	1	768	no ARE	stable
Tnfaip6	21930	tumor necrosis factor alpha induced protein 6	NM_009398	10.6	8.4	6	710	no ARE	1.8(a)
Erbb3	13867	v-erb-b2 erythrobl. leukemia viral oncogene hom. 3 (avian)	NM_010153	10.6	4.9	4	1830	no ARE	stable
Htatip2	53415	HIV-1 tat interactive protein 2, homolog (human)	NM_016865	10.5	0	0	490	no ARE	stable
Cnbd2	70873	cyclic nucleotide binding domain containing 2	NM_027585	10.3	0	0	24	N/A	stable
Lhx9	16876	LIM homeobox protein 9	NM_001025565	9.7	11.1	9	3468	no ARE	stable
Exoc6	107371	exocyst complex component 6	XM_006526591	9.2	2.3	2	1060	N/A	stable
Ptp4a1	19243	protein tyrosine phosphatase 4a1	NM_011200	9.0	18.9	15	2927	Cluster 5	stable
Mrpl50	28028	mitochondrial ribosomal protein L50	NM_178603	9.0	1	1	1593	no ARE	stable
Sin3a	20466	transcriptional regulator, SIN3A (yeast)	NM_011378	9.0	2.3	2	1009	no ARE	stable
Serpini1	20713	serine (or cysteine) peptidase inhibitor, clade I, member 1	NM_009250	9.0	17.65	13	1772	Cluster 5	stable
Reep3	28193	receptor accessory protein 3	NM_178606	8.9	7.5	6	4541	no ARE	stable
Slc35a1	24060	solute carrier family 35, member 1	NM_011895	8.9	2.3	2	779	no ARE	stable
Ptpn2	19255	protein tyrosine phosphatase, non-receptor type 2	NM_001127177	8.8	14.4	12	7091	N/A	stable
Polr1d	20018	polymerase (RNA) I polypeptide D	NM_181730	8.8	0	0	444	no ARE	stable
Sepp1	20363	selenoprotein P, plasma, 1	NM_001042613	8.7	4.9	4	798	no ARE	stable
Smad5	17129	MAD homolog 5 (Drosophila)	NM_008541	8.7	8.25	6	4835	no ARE	stable
Gnptab	432486	N-acetylglucosamine-1-phosphate transferase	NM_001004164	8.7	2.6	2	1424	Cluster 5	stable
Fkbp7	14231	FK506 binding protein 7	NM_010222	8.6	0	0	157	no ARE	stable
Metap1	75624	methionyl aminopeptidase 1	NM_175224	8.5	2.3	2	1377	no ARE	stable
Nmrk1	225994	nicotinamide riboside kinase 1	NM_145497	8.4	1	1	937	no ARE	unknown
Nadk2	68646	NAD kinase 2, mitochondrial	NM_001085410	8.1	13.3	11	2139	N/A	stable
Asf1a	66403	ASF1 anti-silencing function 1 homolog A (S. cerevisiae)	NM_025541	8.1	6.5	5	1348	no ARE	stable
Ugt1a6a	94284	UDP glucuronosyltransferase 1 family, polypeptide A2	NM_145079	8.0	3.6	3	1578	no ARE	stable
Zfp706	68036	zinc finger protein 706	NM_026521	7.9	7.2	6	3925	no ARE	stable
Tmem50b	77975	transmembrane protein 50B	NM_030018	7.8	1	1	1607	no ARE	stable
Met	17295	Met proto-oncogene	NM_008591	7.8	6.5	5	2157	no ARE	stable
Rp2h	19889	retinitis pigmentosa 2 homolog (human)	NM_133669	7.8	19.4	15	3092	no ARE	stable
Npc1	18145	Niemann Pick type C1	NM_008720	7.8	0	0	1226	no ARE	stable
Ntf3	18205	neurotrophin 3	NM_008742	7.8	1.3	1	339	Cluster 5	2.3(a)
Trim59	66949	tripartite motif-containing 59, similar to RING finger 1	NM_025863	7.7	1.3	1	1500	no ARE	unknown
Pdss1	56075	prenyl (solanesyl) diphosphate synthase, subunit 1	NM_019501	7.6	0	0	299	no ARE	stable
Dcun1d1	114893	defective in cullin neddylation 1, dom. contain. 1 (S. cerev.)	NM_033623	7.6	18	15	3511	Cluster 5	stable
Dnajc3	19107	DnaJ (Hsp40) homolog, subfamily C, member 3	NM_008929	7.5	8.2	6	3518	no ARE	stable
Sgpl1	20397	sphingosine phosphate lyase 1	NM_009163	7.5	1	1	2236	no ARE	stable
Fkbp1b	14226	FK506 binding protein 1b	NM_016863	7.5	1	1	520	no ARE	stable
Phf13	230936	PHD finger protein 13	NM_172705	7.5	1.3	1	1922	no ARE	2.0(a)
Bub3	12237	budding uninhibited by benzimidazoles 3 hom. (S. cerev.)	NM_009774	7.4	2.6	2	1010	no ARE	stable
Ero1l	50527	ERO1-like (S. cerevisiae)	NM_015774	7.4	14.4	12	2895	Cluster 5	stable
Tceb1	67923	transcription elongation factor B (SIII), polypeptide 1	NM_026456	7.3	1.3	1	536	no ARE	stable
Camsap1l1	67886	calmodulin regulated spectrin-associated protein 1-like 1	NM_001081360	7.2	16.75	12	2713	N/A	stable
Adh1	11522	alcohol dehydrogenase 1 (class I)	NM_007409	7.1	0	0	146	no ARE	stable
Hey1	15213	hairy/enhancer-of-split related with YRPW motif 1	NM_010423	7.1	1	1	1147	no ARE	2.5(a)
Eaf1	74427	ELL associated factor 1	NM_028932	7.1	10.4	8	3241	Cluster 5	stable
Srxn1	76650	sulfiredoxin 1 homolog (S. cerevisiae)	NM_029688	7.1	2.3	2	2200	no ARE	stable
Sgms2	74442	sphingomyelin synthase 2	NM_028943	7.0	11.85	9	4111	Cluster 5	stable
Taf9	108143	TAF9 RNA polymerase II, TATA box binding protein assoc.	NM_027139	7.0	1.3	1	232	no ARE	stable
Arl2bp	107566	ADP-ribosylation factor-like 2 binding protein	NM_024191	6.9	1.3	1	1339	no ARE	stable
Tpd52	21985	tumor protein D52	NM_009412	6.9	7.2	6	1616	Cluster 5	stable

Txnl1	53382	thioredoxin-like 1	NM_016792	6.8	11.15	8	1502	no ARE	stable
Ino80c	225280	INO80 complex subunit C	NM_172625	6.7	8.5	7	1730	no ARE	stable
Gosr1	53334	golgi SNAP receptor complex member 1	NM_016810	6.6	9	6	3507	Cluster 3	stable
1110007C09Rik	68480	predicted protein, c9orf89 homolog	NM_026738	6.6	1.3	1	169	no ARE	stable
Sbds	66711	Shwachman-Bodian-Diamond syndrome homol. (human)	NM_023248	6.6	3.6	3	617	Cluster 5	stable
Tmod3	50875	tropomodulin 3	NM_016963	6.6	2.6	2	2332	no ARE	stable
Mkks	59030	McKusick-Kaufman syndrome protein	NM_021527	6.6	2.3	2	506	no ARE	stable
Prrx1	18933	paired related homeobox 1	NM_175686	6.6	6.9	6	2480	Cluster 5	stable
Hiat1	15247	hippocampus abundant gene transcript 1	NM_008246	6.5	4.9	4	1123	Cluster 5	2.7(a)
Dna2l	327762	DNA2 DNA replication helicase 2-like (yeast)	NM_177372	6.5	5.2	4	832	no ARE	stable
Trmt112	67674	tRNA methyltransferase 11-2	NM_026306	6.5	0	0	182	no ARE	stable
Mcart1	230125	mitochondrial carrier triple repeat 1	NM_001009949	6.4	4.6	4	3248	no ARE	stable
Rtn3	20168	reticulon 3	NM_053076	6.4	2.6	2	1929	no ARE	stable
Dr1	13486	down-regulator of transcription 1	NM_026106	6.4	14.3	11	1856	no ARE	stable
Dynlrb1	67068	dynein light chain roadblock-type 1	NM_025947	6.4	0	0	282	no ARE	stable
Sgms1	208449	sphingomyelin synthase 1	NM_001168526	6.4	6.2	5	1635	N/A	2.5(a)
Luc7l	66978	Luc7 homolog (S. cerevisiae)-like	NM_028190	6.4	7.25	5	3740	no ARE	stable
Cbx1	12412	chromobox homolog 1 (Drosophila HP1 beta)	NM_007622	6.4	2.6	2	434	no ARE	stable
Pank3	211347	pantothenate kinase 3	NM_145962	6.4	20.4	16	5827	no ARE	stable
Zfp266	77519	zinc finger protein 266	NM_001135019	6.4	17.75	13	4017	N/A	2.0(a)
Bzw1	66882	basic leucine zipper and W2 domains 1	NM_025824	6.4	3.6	3	1433	no ARE	stable
Txndc17	52700	thioredoxin domain containing 17	NM_026559	6.3	3.6	3	1004	no ARE	stable
Adipor1	72674	adiponectin receptor 1	NM_028320	6.3	3.35	2	683	no ARE	stable
Rad21	19357	RAD21 homolog (S. pombe)	NM_009009	6.3	3.9	3	1479	Cluster 5	stable
Bmpr1a	12166	bone morphogenetic protein receptor, type 1A	NM_009758	6.3	8.5	7	3476	no ARE	stable
Nhsl1	215819	NHS-like 1	NM_173390	6.3	5.6	4	1933	N/A	stable
Tmem56	99887	transmembrane protein 56	NM_178936	6.3	30.6	24	5144	Cluster 5	unknown
Rwdd2	69519	RWD domain containing 2	NM_027100	6.2	2.6	2	264	no ARE	2.9(a)
Foxj3	230700	forkhead box J3	NM_172699	6.2	10.4	8	2734	Cluster 5	1.4(a),1.3(c)
Kpna3	16648	karyopherin (importin) alpha 3	NM_008466	6.2	5.9	5	2445	no ARE	stable
Ankrd40	71452	ankyrin repeat domain 40	NM_027799	6.2	3.9	3	2148	no ARE	stable
Mocs2	17434	molybdenum cofactor synthesis 2	NM_001113374	6.2	6.5	5	1063	N/A	stable
Rassf8	71323	Ras association (RalGDS/AF-6) domain family 8	NM_027760	6.2	1.3	1	396	no ARE	2.8(a)
Med13l	76199	mediator complex subunit 13-like	NM_172424	6.1	6.5	5	2595	no ARE	stable
Rnd3	74194	Rho family GTPase 3	NM_028810	6.1	5.2	4	1855	no ARE	2.1(a)
Trp53inp2	68728	tumor protein p53 inducible nuclear protein 2	NM_178111	6.1	1.3	1	3055	no ARE	stable
Ptprk	19272	protein tyrosine phosphatase, receptor type, K	NM_008983	6.0	6.5	5	1542	no ARE	stable
Tiprl	226591	TIP41, TOR signalling pathway regulator-like (S. cerev.)	NM_145513	6.0	11.5	10	3454	Cluster 5	stable
Sh3glb1	54673	SH3-domain GRB2-like B1 (endophilin)	NM_019464	5.9	19.3	13	4503	no ARE	stable
Slc30a1	22782	solute carrier family 30 (zinc transporter), member 1	NM_009579	5.9	7.8	6	3492	Cluster 3	2.5(a),1.6(c)
Tmem106b	71900	transmembrane protein 106B	NM_027992	5.9	14.3	11	4960	no ARE	stable
Il6	16193	interleukin 6	NM_031168	5.8	7.1	5	420	Cluster 5	0.5(d)
Ubfd1	28018	ubiquitin family domain containing 1	NM_138589	5.8	2.6	2	3639	no ARE	stable
Ptx3	19288	pentraxin related gene	NM_008987	5.8	2.6	2	603	no ARE	stable
Dusp16	70686	dual specificity phosphatase 16	NM_130447	5.7	5.1	3	2416	Cluster 4	2.1(a)
Nmd3	97112	NMD3 homolog (S. cerevisiae)	NM_133787	5.7	0	0	145	no ARE	2.8(a)
Hes1	15205	hairy and enhancer of split 1 (Drosophila)	NM_008235	5.7	0	0	367	no ARE	1.2(a)
Slc2a3	20527	solute carrier family 2, member 3	NM_011401	5.6	4.6	4	2136	Cluster 3	1.0(b),stable
Tspyl3	241732	TSPY-like 3	NM_198617	5.6	1.3	1	1897	no ARE	2.0(a)
Bcl7c	12055	B-cell CLL/lymphoma 7C	XM_006507269	5.6	3.3	3	1884	N/A	stable
Ext1	14042	exostoses (multiple) 1	NM_010162	5.6	0	0	417	no ARE	2.5(a)
Fbxl3	50789	F-box and leucine-rich repeat protein 3	NM_015822	5.6	5.2	4	2585	Cluster 5	2.8(a)
Zmynd19	67187	zinc finger, MYND-type containing 19	XM_006498240	5.6	3.3	3	2564	N/A	2.6(a)
Ndufs4	17993	NADH dehydrogenase (ubiquinone) Fe-S protein 4	NM_010887	5.5	4.6	4	968	no ARE	stable
Nov	18133	nephroblastoma overexpressed gene	NM_010930	5.5	6.6	5	1257	no ARE	stable
Camk2n1	66259	calcium/calmodulin-dependent protein kinase II inhibitor 1	NM_025451	5.5	2.6	2	3333	no ARE	stable
Qdpr	110391	quininoid dihydropteridine reductase	NM_024236	5.5	0	0	541	no ARE	stable
Asph	65973	aspartate-beta-hydroxylase	NM_023066	5.4	9.2	8	4210	Cluster 5	stable
Ppp2cb	19053	protein phosphatase 2 , catalytic subunit, beta isoform	NM_017374	5.4	3.9	3	576	Cluster 5	0.7(b),stable
Plekhf2	71801	pleckstrin homol. domain containing, family F member 2	NM_175175	5.4	8.8	7	1965	no ARE	2.3(a)
Fzd4	14366	frizzled homolog 4 (Drosophila)	NM_008055	5.4	3.6	3	1750	no ARE	unknown
Tnpo1	238799	transportin 1	NM_178716	5.4	5.9	5	2575	no ARE	stable
Cep76	225659	centrosomal protein 76	XM_006525899	5.3	16	10	1426	N/A	stable
Tomm7	66169	translocase of outer mitoch. membrane 7 homolog (yeast)	NM_025394	5.3	2.6	2	886	no ARE	stable
Cpne3	70568	copine III	NM_027769	5.3	26.7	19	3932	Cluster 5	stable
Hexim1	192231	hexamethylene bis-acetamide inducible 1	NM_138753	5.2	3.6	3	1733	no ARE	stable
Cap2	67252	CAP, adenylate cyclase-associated protein, 2 (yeast)	NM_026056	5.2	2.6	2	1932	no ARE	stable
Snapc3	77634	small nuclear RNA activating complex, polypeptide 3	NM_029949	5.2	1.3	1	153	no ARE	1.4(b),stable
Sc5d	235293	sterol-C5-desaturase homolog (S. cerevisae)	NM_172769	5.2	2.6	2	1165	no ARE	stable
Sept2	18000	septin 2	NM_010891	5.2	7.5	6	2039	Cluster 5	stable
Rnf38	73469	ring finger protein 38	NM_175201	5.2	7.2	6	3382	no ARE	stable
Rnf7	19823	ring finger protein 7	NM_011279	5.2	0	0	712	no ARE	stable
Vta1	66201	Vps20-associated 1 homolog (S. cerevisiae)	NM_025418	5.2	2.3	2	363	no ARE	unknown
Prps1	19139	phosphoribosyl pyrophosphate synthetase 1	NM_021463	5.2	4.35	3	878	no ARE	stable
Tpbg	21983	trophoblast glycoprotein	NM_011627	5.2	4.9	4	1795	no ARE	stable
Dnaja4	58233	DnaJ (Hsp40) homolog, subfamily A, member 4	NM_021422	5.1	3.35	2	1605	no ARE	stable
Fgfr3	14184	fibroblast growth factor receptor 3	NM_008010	5.1	6.2	5	1508	Cluster 5	stable
Lats2	50523	large tumor suppressor 2	NM_015771	5.1	7.9	6	1648	Cluster 5	2.7(a)
Shroom3	27428	shroom family member 3	NM_015756	5.1	1.3	1	1035	no ARE	2.7(a)
Commd10	69456	COMM domain containing 10	NM_178377	5.1	2.6	2	929	no ARE	stable
Tmem30a	69981	transmembrane protein 30A	NM_133718	5.1	6.2	5	2365	no ARE	stable
Arsb	11881	arylsulfatase B	NM_009712	5.1	3.6	3	2239	no ARE	stable
Rhobtb3	73296	Rho-related BTB domain containing 3	NM_028493	5.1	11.1	9	2809	no ARE	stable
Mtpn	14489	myotrophin	NM_008098	5.1	12.1	9	3407	Cluster 5	stable
Arl4a	11861	ADP-ribosylation factor-like 4A	NM_007487	5.1	11.7	9	2853	no ARE	2.2(a),0.8(c)
Ccnt2	72949	cyclin T2	NM_028399	5.0	6.2	5	1274	no ARE	0.7(b),1.3(c)
Tnfrsf23	79201	tumor necrosis factor receptor superfamily, member 23	NM_024290	5.0	2.3	2	2736	no ARE	stable
Abhd5	67469	abhydrolase domain containing 5	NM_026179	5.0	7.9	7	1921	no ARE	stable
Pisd	320951	phosphatidylserine decarboxylase	NM_177298	5.0	1.3	1	916	Cluster 5	stable

Ptgs2	19225	prostaglandin-endoperoxide synthase 2	NM_011198	5.0	21.05	12	2235	Cluster 3	stable
Zfp386	56220	zinc finger protein 386 (Kruppel-like)	NM_019565	5.0	6.3	6	2665	no ARE	stable
Gltp	56356	glycolipid transfer protein	NM_019821	5.0	0	0	918	no ARE	stable
Nutf2	68051	nuclear transport factor 2	NM_026532	5.0	2	2	1484	no ARE	stable
Cldnd1	224250	claudin domain containing 1	NM_171826	5.0	7.7	5	1188	Cluster 4	stable
Pkn2	109333	protein kinase N2	NM_178654	4.9	6.5	5	2909	no ARE	stable
Galk2	69976	galactokinase 2	NM_175154	4.9	3.6	3	1019	no ARE	stable
Tmed10	68581	transmembrane emp24-like trafficking protein 10 (yeast)	NM_026775	4.9	1	1	2814	no ARE	stable
Cdk2ap1	13445	CDK2 (cyclin-dependent kinase 2)-associated protein 1	NM_013812	4.8	1.3	1	653	no ARE	stable
Dhrs7	66375	dehydrogenase/reductase (SDR family) member 7	NM_025522	4.8	1.3	1	234	no ARE	stable
Gin1	252876	gypsy retrotransposon integrase 1	NM_026250	4.8	1.3	1	400	N/A	2.5(a)
Tsfm	66399	Ts translation elongation factor, mitochondrial	NM_025537	4.8	1.3	1	188	Cluster 5	stable
Josd3	75316	Josephin domain containing 3	NM_027261	4.8	0	0	412	no ARE	stable
Enpp2	18606	ectonucleotide pyrophosphatase/phosphodiesterase 2	NM_015744	4.8	2.3	2	464	Cluster 5	stable
Gng12	14701	guanine nucleotide binding protein (G protein), gamma 12	NM_025278	4.7	8.8	7	3772	Cluster 5	stable
Gclm	14630	glutamate-cysteine ligase , modifier subunit	NM_008129	4.7	0	0	546	no ARE	stable
H2afv	77605	H2A histone family, member V	NM_029938	4.7	1.3	1	1076	N/A	stable
Ccdc50	67501	coiled-coil domain containing 50	NM_001025615	4.7	16.3	13	5966	no ARE	stable
Nipa1	233280	non imprinted in Prader-Willi/Angelman syndrome 1 hom.	NM_153578	4.7	3.9	3	908	no ARE	stable
Slc11a1	18173	solute carrier family 11, member 1	NM_013612	4.7	0	0	545	no ARE	stable
Ier5l	72500	immediate early response 5-like	NM_030244	4.7	0	0	102	no ARE	1.4(a)
Coprs	66423	coordinator of PRMT5, differentiation stimulator	NM_025556	4.7	0	0	242	no ARE	stable
Lass2	76893	longevity assurance homolog 2 (S. cerevisiae)	NM_029789	4.7	1.3	1	803	no ARE	stable
Tbpl1	237336	TATA box binding protein-like 1	NM_011603	4.7	3.6	3	1944	Cluster 5	2.2(a)
Mturn	68235	maturin, neural progen. diff. regul. homolog (Xenopus)	NM_001289740	4.6	10.3	7	4747	N/A	unknown
Pdlim5	56376	similar to PDZ and LIM domain protein 5	NM_019808	4.6	6.2	5	3110	Cluster 5	stable
Kif26b	269152	kinesin family member 26B	NM_001161665	4.6	0	0	632	N/A	unknown
Poc5	67463	POC5 centriolar protein homolog	NM_026173	4.6	2.3	2	968	no ARE	2.7(a)
Fusip1	14105	FUS interacting protein (serine-arginine rich) 1	NM_010178	4.6	14.7	11	2666	Cluster 5	stable
Sat1	20229	spermidine/spermine N1-acetyl transferase 1	NM_009121	4.6	2.6	2	476	no ARE	stable
Myef2	17876	Myelin basic protein expression factor 2, repressor	NM_010852	4.6	5.4	3	1132	no ARE	stable
Mettl16	67493	methyltransferase like 16	NM_026197	4.6	1.3	1	934	no ARE	stable
Mrps6	121022	mitochondrial ribosomal protein S6	NM_080456	4.5	1	1	305	no ARE	stable
Alg10b	380959	asparagine-linked glycosylation 10B	NM_001033441	4.5	8.2	7	2117	Cluster 5	stable
Slc26a4	23985	solute carrier family 26, member 4	NM_011867	4.5	0	0	525	no ARE	stable
1810011O10Rik	69068	predicted protein, C8orf4 homolog	NM_026931	4.5	1.3	1	960	no ARE	1.1(a)
Pf4	56744	platelet factor 4	NM_019932	4.5	1.3	1	150	no ARE	0.7(b),stable
Ap2b1	71770	Adaptor-related protein complex 2, beta 1 subunit	NM_027915	4.5	2.3	2	2391	no ARE	stable
Stard4	170459	StAR-related lipid transfer (START) domain containing 4	NM_133774	4.4	3.9	3	2165	no ARE	stable
Hbp1	73389	high mobility group box transcription factor 1	NM_153198	4.4	3.9	3	1035	no ARE	stable
Slc16a10	72472	solute carrier family 16, member 10	NM_028247	4.4	0	0	1132	no ARE	stable
Smad6	17130	Mothers against decapentaplegic homolog 6 (SMAD 6)	NM_008542	4.4	2.6	2	448	Cluster 5	1.9(a)
Slk	20874	STE20-like kinase (yeast)	NM_009289	4.4	4.9	4	3170	no ARE	stable
Creb1	12912	cAMP responsive element binding protein 1	NM_001037726	4.4	20	17	7180	N/A	stable
Anxa4	11746	annexin A4	NM_013471	4.4	2.6	2	924	no ARE	stable
Atl3	109168	atlastin GTPase 3	NM_146091	4.4	18.1	14	4567	no ARE	stable
BC031181	407819	putative NFkB activating protein, C18orf32 homolog	NM_001001181	4.4	7.25	5	625	Cluster 5	stable
Mettl9	59052	methyltransferase like 9	NM_021554	4.3	1.3	1	527	no ARE	stable
Cbll1	104836	Casitas B-lineage lymphoma-like 1	NM_134048	4.3	9.1	7	2450	no ARE	2.2(a)
Api5	11800	apoptosis inhibitor 5	NM_007466	4.3	2.6	2	2032	no ARE	stable
Ankrd13a	68420	ankyrin repeat domain 13a	NM_026718	4.3	0	0	1460	no ARE	stable
Rfk	54391	riboflavin kinase	NM_019437	4.3	7.8	6	1746	no ARE	stable
Acot13	66834	acyl-CoA thioesterase 13	NM_025790	4.3	0	0	80	no ARE	stable
Atg12	67526	autophagy-related 12 (yeast)	NM_026217	4.3	8.8	7	2033	Cluster 5	stable
Hsdl2	72479	hydroxysteroid dehydrogenase like 2	NM_024255	4.3	2.6	2	963	no ARE	stable
Cdc42	12540	cell division cycle 42 homolog (S. cerevisiae)	NM_009861	4.3	1	1	1381	no ARE	stable
Dgat2	67800	diacylglycerol O-acyltransferase 2	NM_026384	4.3	2.3	2	883	no ARE	stable
Ptpmt1	66461	protein tyrosine phosphatase, mitochondrial 1	NM_025576	4.3	1.3	1	472	no ARE	stable
Kif21a	16564	kinesin family member 21A	NM_016705	4.2	3.6	3	1175	no ARE	stable
Bri3bp	76809	Bri3 binding protein	NM_029752	4.2	7.8	6	6091	Cluster 5	stable
Uap1	107652	UDP-N-acetylglucosamine pyrophosphorylase 1	NM_133806	4.2	1.3	1	463	Cluster 5	stable
Tpd52l2	66314	tumor protein D52-like 2	NM_025482	4.2	1.3	1	2787	no ARE	stable
Rab6b	270192	RAB6B, member RAS oncogene family	NM_173781	4.2	2.8	2	4024	no ARE	stable
Ypel5	383295	yippee-like 5 (Drosophila)	XM_006524539	4.2	3.9	3	1596	N/A	stable
Prr3	75210	proline-rich polypeptide 3	NM_145487	4.2	0	0	1168	no ARE	stable
Ube2a	22209	ubiquitin-conjugating enzyme E2A, RAD6 hom. (S. cerev.)	NM_019668	4.2	6.5	5	1082	no ARE	stable
Prc1	233406	protein regulator of cytokinesis 1	NM_145150	4.2	1	1	1098	Cluster 5	stable
Bmp4	12159	bone morphogenetic protein 4	NM_007554	4.2	2.6	2	339	no ARE	2.5(a),0.9(c)
Tax1bp3	76281	Tax1 (human T-cell leukemia virus type I) binding prot. 3	NM_029564	4.2	1.3	1	821	no ARE	stable
Nr4a2	18227	Nuclear receptor subfamily 4, group A, member 2	NM_013613	4.2	3.3	3	930	no ARE	0.7(b),stable
Prr11	270906	proline rich 11	NM_175563	4.1	9.3	7	2641	no ARE	stable
Hivep2	15273	human immunodef. virus type I enhancer binding prot. 2	NM_010437	4.1	3.9	3	1662	Cluster 5	2.0(a)
Cntf	12803	ciliary neurotrophic factor	NM_170786	4.1	0	0	370	no ARE	unknown
Glud1	14661	glutamate dehydrogenase 1	NM_008133	4.1	2.6	2	1268	Cluster 5	stable
Rnf113a2	66381	ring finger protein 113A2	NM_025525	4.1	0	0	243	no ARE	2.4(a),1.5(b)
Stxbp6	217517	syntaxin binding protein 6 (amisyn)	NM_144552	4.1	6.5	5	3368	Cluster 5	stable
Ddx31	227674	DEAD/H (Asp-Glu-Ala-Asp/His) box polypeptide 31	NM_001033294	4.1	0	0	808	no ARE	stable
Fam214a	235493	family with sequence similarity 214, member A	NM_153584	4.1	5.95	4	800	no ARE	stable
Fyttd1	69823	forty-two-three domain containing 1	NM_027226	4.1	16	13	3277	Cluster 5	stable
Ppil5	69706	peptidylprolyl isomerase (cyclophilin) like 5	NM_001081406	4.0	3.6	3	171	N/A	stable
Eif2s2	67204	eukaryotic translation initiation factor 2, subunit 2 (beta)	NM_026030	4.0	0	0	1315	no ARE	stable
Asun	71177	asunder, spermatogenesis regulator	NM_138757	4.0	1.3	1	326	no ARE	stable
Ifi30	65972	interferon gamma inducible protein 30	NM_023065	4.0	1.3	1	218	no ARE	stable
Mtmr9	210376	myotubularin related protein 9	NM_177594	4.0	0	0	505	no ARE	stable
Bmper	73230	BMP-binding endothelial regulator	NM_028472	4.0	6.5	5	1183	no ARE	stable
Csda	56449	cold shock domain protein A	NM_139117	4.0	2.6	2	581	no ARE	stable
Uba3	22200	ubiquitin-like modifier activating enzyme 3	NM_011666	4.0	4.9	4	964	no ARE	stable
Znhit3	448850	zinc finger, HIT type 3	NM_001005223	4.0	4.65	3	512	no ARE	2.9(a)
Grb2	14784	growth factor receptor bound protein 2	NM_008163	4.0	2.6	2	1639	no ARE	stable

Rab2a	59021	RAB2A, member RAS oncogene family	NM_021518	4.0	1.3	1	1199	no ARE	stable
Alox5ap	11690	arachidonate 5-lipoxygenase activating protein	NM_009663	3.9	2.3	2	331	no ARE	stable
Mfsd1	66868	major facilitator superfamily domain containing 1	NM_025813	3.9	2.3	2	1533	no ARE	stable
Lypla1	18777	lysophospholipase 1	NM_008866	3.9	10.4	8	1733	Cluster 5	stable
Fam60a	56306	family with sequence similarity 60, member A	NM_019643	3.9	11.5	7	1814	Cluster 3	stable
Slc40a1	53945	solute carrier family 40, member 1	NM_016917	3.9	2.6	2	1336	Cluster 5	stable
Ube2r2	67615	ubiquitin-conjugating enzyme E2R 2	NM_026275	3.9	4.9	4	2472	no ARE	stable
Pten	19211	phosphatase and tensin homolog	NM_008960	3.8	26	20	6149	no ARE	stable
Aldh1a7	26358	aldehyde dehydrogenase family 1, subfamily A7	NM_011921	3.8	1	1	510	no ARE	stable
Bcl6	12053	B-cell leukemia/lymphoma 6	NM_009744	3.8	1.3	1	1114	no ARE	1.3(a),1.4(c)
Nudt19	110959	nudix (nucleoside diphosph. linked moiety X)-type motif 19	NM_033080	3.8	0	0	729	no ARE	stable
Zfand4	67492	zinc finger, AN1-type domain 4	NM_001290338	3.8	2.6	2	786	N/A	stable
Pde6d	18582	phosphodiesterase 6D, cGMP-specific, rod, delta	NM_008801	3.8	1.3	1	479	no ARE	stable
Slc25a46	67453	solute carrier family 25, member 46	NM_026165	3.8	16.7	13	3023	Cluster 5	stable
Stat3	20848	signal transducer and activator of transcription 3	NM_213659	3.8	2.6	2	1895	Cluster 5	stable
Klhdc9	68874	kelch domain containing 9	NM_001033039	3.8	1.3	1	365	no ARE	stable
Ccne2	12448	cyclin E2	NM_009830	3.8	11.8	7	1681	Cluster 3	stable
Cdca7l	217946	cell division cycle associated 7 like	NM_146040	3.8	2.3	2	1236	no ARE	stable
Rankl	192193	ER degradation enhancer, mannosidase alpha-like 1	NM_138677	3.8	6.5	5	3800	Cluster 5	stable
Xrcc6bp1	68876	XRCC6 binding protein 1	NM_026858	3.8	0	0	252	no ARE	stable
Ogfrl1	70155	opioid growth factor receptor-like 1	NM_001081079	3.8	13.7	11	3304	N/A	stable
Spin1	20729	spindlin	NM_146043	3.8	10.5	9	3335	Cluster 5	unknown
Lrrc1	214345	leucine rich repeat containing 1	NM_172528	3.8	3.9	3	1299	no ARE	stable
Eif3j1	78655	eukaryotic translation initiation factor 3, subunit J1	NM_144545	3.8	6.2	5	1555	no ARE	stable
Kcmf1	74287	potassium channel modulatory factor 1	NM_019715	3.8	7.8	6	1711	no ARE	stable
Fam96a	68250	family with sequence similarity 96, member A	NM_026635	3.8	1.3	1	534	no ARE	stable
Cxcl15	20309	chemokine (C-X-C motif) ligand 15	NM_011339	3.8	2.6	2	1586	Cluster 5	stable
Zfp248	72720	zinc finger protein 248	NM_028335	3.8	7.65	6	1582	no ARE	1.6(a)
March5	69104	membrane-associated ring finger (C3HC4) 5	NM_027314	3.7	10.8	6	2923	no ARE	stable
Psmd10	53380	proteosome 26S subunit, non-ATPase, 10	NM_016883	3.7	1.3	1	716	no ARE	0.9(b),stable
Smim14	68552	small integral membrane protein 14	NM_133697	3.7	4.9	4	1650	no ARE	stable
Tgoln1	22134	trans-golgi network protein	NM_009444	3.7	3.6	3	1147	no ARE	stable
Gla	11605	galactosidase, alpha	NM_013463	3.7	4.3	4	1698	no ARE	stable
Tmed4	103694	transmembrane emp24 prot. transport domain cont. 4	NM_134020	3.7	0	0	957	no ARE	stable
Tor1aip1	208263	torsin A interacting protein 1	NM_001160019	3.7	10.5	8	2326	N/A	stable
Inip	66209	INTS3 and NABP interacting protein	NM_001013577	3.7	3.6	3	2661	no ARE	stable
Pnp	18950	purine-nucleoside phosphorylase	NM_013632	3.7	4.6	4	1751	Cluster 5	stable
Tmem64	100201	transmembrane protein 64	NM_181401	3.7	17	14	3403	Cluster 5	stable
Kpna1	16646	karyopherin (importin) alpha 1	NM_008465	3.7	8.9	7	2254	no ARE	stable
Mark1	226778	MAP/microtubule affinity-regulating kinase 1	NM_145515	3.7	5.95	4	2100	no ARE	stable
Fam103a1	67148	family with sequence similarity 103, member A1	NM_025997	3.7	3.9	3	932	Cluster 5	stable
Cep19	66994	centrosomal protein 19	NM_025892	3.7	0	0	787	no ARE	stable
Cdk1	12534	cyclin-dependent kinase 1	NM_007659	3.7	4.3	4	1863	no ARE	stable
6330416G13Rik	230279	uncharacterized protein C9orf91 homolog	NM_144905	3.7	1.3	1	2388	no ARE	stable
Prpf31	68988	PRP31 pre-mRNA processing factor 31 homol. (yeast)	NM_027328	3.7	1.3	1	1533	no ARE	stable
Mras	17532	muscle and microspikes RAS	NM_008624	3.7	1	1	3011	no ARE	stable
Klhl11	217194	kelch-like 11 (Drosophila)	NM_172565	3.7	0	0	252	no ARE	2.8(a)
Rrm2	20135	ribonucleotide reductase M2	NM_009104	3.7	2.3	2	933	no ARE	stable
Cklf	75458	chemokine-like factor	NM_001037841	3.7	1	1	1423	no ARE	stable
Scamp1	107767	secretory carrier membrane protein 1	NM_029153	3.7	9.1	7	2555	no ARE	stable
Arl6	56297	ADP-ribosylation factor-like 6	NM_019665	3.6	3.9	3	462	no ARE	stable
Rfc3	69263	replication factor C (activator 1) 3	NM_027009	3.6	0	0	25	N/A	stable
Dlgap1	224997	discs, large (Drosophila) homolog-associated protein 1	NM_177639	3.6	17.2	13	3173	no ARE	stable
Lgals2	107753	lectin, galactose-binding, soluble 2	NM_025622	3.6	1.3	1	117	no ARE	stable
Kitl	17311	kit ligand	NM_013598	3.6	18.8	14	4431	no ARE	stable
Mrpl49	18120	mitochondrial ribosomal protein L49	NM_026246	3.6	4.3	3	1206	no ARE	stable
Slc20a2	20516	solute carrier family 20, member 2	NM_011394	3.6	2.3	2	1196	no ARE	2.9(a)
Eif4b	75705	eukaryotic translation initiation factor 4B	NM_145625	3.6	1.3	1	1841	no ARE	stable
Dnajc12	30045	DnaJ (Hsp40) homolog, subfamily C, member 12	NM_013888	3.6	4.65	3	573	no ARE	stable
Fut8	53618	fucosyltransferase 8	NM_016893	3.6	1	1	680	no ARE	stable
Kif14	381293	kinesin family member 14	NM_001287179	3.6	13.05	9	3833	N/A	stable
Smim8	66291	small integral membrane protein 8	NM_025471	3.6	0	0	317	no ARE	stable
Dab2	13132	disabled homolog 2 (Drosophila)	NM_023118	3.6	4.6	4	1936	Cluster 5	stable
Snap29	67474	synaptosomal-associated protein	NM_023348	3.6	3.6	3	2526	no ARE	stable
Derl1	67819	Der1-like domain family, member 1	NM_024207	3.6	3.35	2	2266	no ARE	stable
Zbtb44	235132	zinc finger and BTB domain containing 44	NM_172765	3.6	27.95	21	7149	no ARE	2.9(a)
Lsm2	27756	LSM2 hom., U6 small nuclear RNA associated (S.cer.)	NM_030597	3.6	0	0	259	no ARE	stable
Osgin2	209212	oxid. stress induced growth inhibitor family member 2	NM_145950	3.6	11.3	9	847	Cluster 5	stable
Cmc1	67899	COX assembly mitochondrial protein 1	NM_026442	3.6	2.6	2	706	no ARE	stable
Rbm25	67039	RNA binding motif protein 25	NM_027349	3.6	2.6	2	1514	N/A	unknown
Cab39	12283	calcium binding protein 39	NM_133781	3.6	7.8	6	2356	no ARE	stable
Polr2d	69241	polymerase (RNA) II (DNA directed) polypeptide D	NM_027101	3.6	0	0	514	no ARE	stable
Otud1	71198	OTU domain containing 1	NM_027715	3.6	3.9	3	1164	N/A	1.3(a)
Fabp4	11770	fatty acid binding protein 4, adipocyte	NM_024406	3.6	1.3	1	182	no ARE	stable
Nkiras1	69721	NFKB inhibitor interacting Ras-like protein 1	NM_023526	3.5	14.45	11	3814	no ARE	stable
Dazap2	23994	DAZ associated protein 2	NM_011873	3.5	3.6	3	1303	no ARE	stable
Hsbp1	68196	heat shock factor binding protein 1	NM_024219	3.5	2.3	2	909	no ARE	stable
Yipf4	67864	Yip1 domain family, member 4	NM_026417	3.5	10.05	7	1169	Cluster 5	stable
Car5b	56078	carbonic anhydrase 5b, mitochondrial	NM_181315	3.5	5.2	4	2345	no ARE	stable
Ubap1	67123	ubiquitin-associated protein 1	NM_023305	3.5	0	0	964	no ARE	stable
Abhd13	68904	abhydrolase domain containing 13	NM_001081119	3.5	12.1	10	3674	N/A	3.0(a)
Ptbp2	56195	polypyrimidine tract binding protein 2	NM_019550	3.5	7.5	6	1586	no ARE	stable
Chp1	56398	calcineurin-like EF hand protein 1	NM_019769	3.5	6.65	5	1856	no ARE	stable
Mllt11	56772	myeloid/lymphoid leukemia (trithorax homol., Drosoph.)	NM_019914	3.5	19.2	10	1641	Cluster 1	2.0(a)
Smndc1	76479	survival motor neuron domain containing 1	NM_172429	3.5	3.9	3	1250	no ARE	0.7(b),stable
Zfand1	66361	zinc finger, AN1-type domain 1	NM_025512	3.5	3.9	3	834	no ARE	stable
Gsto1	14873	glutathione S-transferase omega 1	NM_010362	3.5	0	0	340	no ARE	stable
Egln3	112407	EGL nine homolog 3 (C. elegans)	NM_028133	3.5	4.9	4	1660	Cluster 5	stable
Tnnc1	21924	troponin C, cardiac/slow skeletal	NM_009393	3.5	0	0	174	no ARE	stable

Zfp644	52397	hypothetical Zinc finger protein 644	NM_026856	3.5	11.4	9	1500	no ARE	2.3(a)
Mpp6	56524	membrane prot., palmitoylated 6 (MAGUK p55 member 6)	NM_019939	3.5	0	0	47	no ARE	stable
Crct1	74175	cysteine-rich C-terminal 1	NM_028798	3.5	0	0	315	no ARE	stable
Trim21	20821	tripartite motif protein 21	NM_009277	3.5	1.3	1	1180	no ARE	2.4(a)
Nip7	66164	nucleolar pre-rRNA processing protein	NM_025391	3.5	2.6	2	2435	no ARE	stable
Ormdl2	66844	ORM1-like 2 (S. cerevisiae)	NM_024180	3.5	3.9	3	1423	Cluster 5	stable
Coq10b	67876	coenzyme Q10 homolog B (S. cerevisiae)	NM_026424	3.5	3.9	3	914	no ARE	stable
Ndufb4	68194	NADH dehydrogenase 1 beta subcomplex 4	NM_026610	3.5	0	0	55	no ARE	stable
Arl5b	75869	ADP-ribosylation factor-like 5B	NM_029466	3.5	23.3	15	2887	Cluster 3	2.7(a)
Lsm3	67678	LSM3 hom., U6 small nuclear RNA assoc. (S. cerev.)	NM_026309	3.4	0	0	299	no ARE	stable
Zfp825	235956	zinc finger protein 825	NM_146231	3.4	0	0	429	no ARE	stable
Lysmd2	70082	LysM, putative peptidoglycan-binding, domain cont. 2	NM_027309	3.4	2.6	2	519	no ARE	stable
Tmbim4	68212	transmembrane BAX inhibitor motif containing 4	NM_026617	3.4	0	0	71	no ARE	stable
Ube2d3	66105	ubiquitin-conjugating enzyme E2D 3	NM_025356	3.4	12.7	10	1938	no ARE	stable
Pigx	72084	phosphatidylinositol glycan, class X	NM_024464	3.4	0	0	39	no ARE	stable
Rab27b	80718	RAB27b, member RAS oncogene family	NM_030554	3.4	17.3	14	6057	Cluster 5	unknown
Hmces	232210	5-hydroxymethylcytosine (hmC) binding, ES cell specific	NM_173737	3.4	0	0	302	no ARE	stable
Antxr2	71914	anthrax toxin receptor 2	NM_133738	3.4	4.3	4	1886	no ARE	stable
Cks1b	54124	CDC28 protein kinase 1b	NM_016904	3.4	2.6	2	439	no ARE	stable
Dusp12	80915	dual specificity phosphatase 12	NM_023173	3.4	1.3	1	260	no ARE	stable
Bnip3l	12177	BCL2/adenovirus E1B interacting protein 3-like	NM_009761	3.4	5.2	4	2487	no ARE	0.9(b),stable
Slu7	193116	SLU7 splicing factor homolog (S. cerevisiae)	NM_148673	3.4	3.9	3	1790	no ARE	stable
Nadk	192185	NAD kinase	NM_138671	3.4	1.3	1	1542	no ARE	stable
Banf1	23825	barrier to autointegration factor 1	NM_011793	3.4	0	0	379	no ARE	stable
Hadh	15107	L-3-hydroxyacyl-Coenzyme A dehydrogenase, short chain	NM_008212	3.4	0	0	762	no ARE	stable
Thbs2	21826	thrombospondin 2	NM_011581	3.4	14.2	10	2013	no ARE	stable
Mcfd2	193813	multiple coagulation factor deficiency 2	NM_176808	3.4	1.3	1	1483	Cluster 5	stable
Dpm1	13480	dolichol-phosphate (beta-D) mannosyltransferase 1	NM_010072	3.4	7.5	6	1484	no ARE	stable
Exosc6	72544	exosome component 6	NM_028274	3.4	1	1	492	N/A	unknown
Ube2f	67921	ubiquitin-conjugating enzyme E2F (putative)	XM_006529838	3.4	2.6	2	1135	N/A	stable
Neo1	18007	neogenin	NM_008684	3.4	4.6	4	2596	no ARE	stable
Myl12b	67938	myosin, light chain 12B, regulatory	NM_023402	3.4	1.3	1	361	no ARE	stable
Cxxc5	67393	CXXC finger 5	NM_133687	3.3	1.3	1	1033	no ARE	2.9(a)
Rbm4	19653	RNA binding motif protein 4	NM_009032	3.3	5.3	5	1303	no ARE	stable
Atp6v1g1	66290	ATPase, H+ transporting, lysosomal V1 subunit G1	NM_024173	3.3	2.3	2	599	no ARE	stable
Gpr161	240888	G protein-coupled receptor 161	XM_006496850	3.3	7.7	5	5027	N/A	stable
Ly6c1	17067	lymphocyte antigen 6 complex, locus C1	NM_010741	3.3	1	1	403	no ARE	stable
Rpia	19895	ribose 5-phosphate isomerase A	NM_009075	3.3	1.3	1	851	no ARE	stable
Dnaaf2	109065	dynein, axonemal assembly factor 2	NM_027269	3.3	1.3	1	74	Cluster 5	stable
Me2	107029	malic enzyme 2, NAD(+)-dependent, mitochondrial	NM_145494	3.3	1.3	1	586	no ARE	stable
Rnf141	67150	ring finger protein 141	NM_025999	3.3	0	0	165	Cluster 5	stable
Ppfibp1	67533	PTPRF interacting protein, binding prot. 1 (liprin beta 1)	NM_001170433	3.3	3.3	3	1641	N/A	stable
G3bp2	23881	GTPase activating prot. SH3 domain binding protein 2	NM_011816	3.3	6.2	5	2635	Cluster 5	stable
Med21	108098	mediator complex subunit 21	NM_025315	3.3	0	0	313	no ARE	stable
Lyrm4	380840	LYR motif containing 4	NM_201358	3.3	2.6	2	1025	no ARE	unknown
Trim7	94089	tripartite motif protein 7	NM_053166	3.3	6.5	5	1900	N/A	stable
Rbbp4	19646	retinoblastoma binding protein 4	NM_009030	3.3	6.9	6	2992	Cluster 5	stable
1600012H06Rik	67912	validated uncharacterized protein, c6orf120 homolog	NM_026451	3.3	5.3	4	1769	no ARE	2.5(a)
Cttnbp2nl	80281	CTTNBP2 N-terminal like	NM_030249	3.3	4.9	4	2706	no ARE	2.9(a)
Crtc3	70461	CREB regulated transcription coactivator 3	NM_173863	3.3	1	1	3111	no ARE	stable
Txnrd1	50493	thioredoxin reductase 1	NM_015762	3.3	6.1	4	1679	no ARE	1.5(b),stable
Chmp2b	68942	chromatin modifying protein 2B	NM_026879	3.3	5.2	4	1007	no ARE	stable
Rap1b	215449	RAS related protein 1b	NM_024457	3.3	3.6	3	1167	no ARE	stable
Atpbd1c	68080	ATP binding domain 1 family, member C	NM_024216	3.3	2.6	2	459	no ARE	stable
Cacul1	78832	CDK2 associated, cullin domain 1	NM_030197	3.3	4	4	4338	no ARE	stable
Mtfr1	67472	mitochondrial fission regulator 1	NM_026182	3.3	8.5	7	1721	Cluster 5	stable
Uhrfbp1l	75089	UHRF1 (ICBP90) binding protein 1-like	NM_029166	3.3	8.2	6	1818	no ARE	stable
Tmem85	68032	transmembrane protein 85	NM_026519	3.3	1.3	1	300	no ARE	stable
Anapc16	52717	anaphase promoting complex subunit 16	NM_025514	3.3	1.3	1	814	no ARE	stable
Psme3	19192	proteaseome (prosome, macropain) 28 subunit, 3	NM_011192	3.3	1.3	1	1659	no ARE	stable
Krr1	52705	KRR1, small subunit processome comp., homol. (yeast)	NM_178610	3.3	8	6	3749	no ARE	stable
Rnf4	19822	ring finger protein 4	NM_011278	3.3	1.3	1	2050	no ARE	stable
2310030G06Rik	66952	predicted protein, c11orf52 homolog	NM_025865	3.2	2.6	2	864	no ARE	2.2(a)
Gabpa	14390	GA repeat binding protein, alpha	NM_008065	3.2	12.15	9	3193	Cluster 5	stable
Csnk2a1	12995	casein kinase II, alpha 1 polypeptide	NM_007788	3.2	10.8	9	2738	Cluster 5	stable
Spg21	27965	spastic paraplegia 21 homolog (human)	NM_138584	3.2	1.3	1	1521	no ARE	stable
Plekha3	83435	pleckstrin homol. domain-containing, family A member 3	NM_031256	3.2	14	9	1833	Cluster 3	stable
Tmem9b	56786	TMEM9 domain family, member B	NM_020050	3.2	2.6	2	1000	no ARE	stable
Ube2j2	140499	ubiquitin-conjugating enzyme E2, J2 homolog (yeast)	NM_021402	3.2	6.4	4	2239	Cluster 3	stable
Pgpep1	66522	pyroglutamyl-peptidase I	NM_023217	3.2	5.4	3	4112	no ARE	stable
Sgcb	24051	sarcoglycan, beta (dystrophin-associated glycoprotein)	NM_011890	3.2	9.8	8	2746	no ARE	stable
Emc7	73024	ER membrane protein complex subunit 7	XM_006500254	3.2	9.5	8	5019	N/A	stable
Elf2	69257	E74-like factor 2	NM_023502	3.2	7.5	6	3590	no ARE	stable
Ikzf5	67143	IKAROS family zinc finger 5	NM_175115	3.2	21.95	16	3038	Cluster 5	stable
Yaf2	67057	YY1 associated factor 2	NM_024189	3.2	5.2	4	1548	no ARE	stable
Zfand3	21769	zinc finger, AN1-type domain 3	NM_148926	3.2	2.3	2	1824	no ARE	stable
Map2k6	26399	mitogen activated protein kinase kinase 6	NM_011943	3.2	5.2	4	771	Cluster 5	stable
Alg14	66789	asparagine-linked glycosylation 14 homolog (yeast)	NM_024178	3.2	2.6	2	225	Cluster 5	stable
Etf1	225363	eukaryotic translation termination factor 1	NM_144866	3.2	8.2	7	2216	no ARE	stable
Fgfr1	14182	fibroblast growth factor receptor 1	NM_010206	3.2	3.6	3	1797	no ARE	stable
Stx7	53331	syntaxin 7	NM_016797	3.2	8	5	1239	Cluster 4	stable
Sms	20603	spermine synthase	NM_009214	3.2	9.1	7	2183	no ARE	stable
Tiparp	99929	TCDD-inducible poly(ADP-ribose) polymerase	NM_178892	3.2	11.1	9	1953	Cluster 3	1.1(a)
Ccl9	20308	chemokine (C-C motif) ligand 9	NM_011338	3.2	6.65	5	2467	no ARE	unknown
Farsb	23874	phenylalanine-tRNA synthetase-like, beta subunit	NM_011811	3.2	1.3	1	384	no ARE	stable
Ap1s2	108012	adaptor-related protein complex 1, sigma 2 subunit	NM_026887	3.2	13.2	10	2587	no ARE	stable
Cd24a	12484	CD24a antigen	NM_009846	3.2	3.9	3	1509	no ARE	stable
Acvr1	11477	activin A receptor, type 1	NM_007394	3.2	7.5	6	1115	Cluster 5	2.5(a),1.1(b)
Casp9	12371	caspase 9	NM_015733	3.2	2.6	2	2291	no ARE	stable

Ube2c	68612	ubiquitin-conjugating enzyme E2C	NM_026785	3.2	0	0	340	no ARE	stable
Pank2	74450	pantothenate kinase 2 (Hallervorden-Spatz syndrome)	NM_153501	3.2	6.2	5	2865	no ARE	stable
Senp8	71599	SUMO/sentrin specific peptidase 8	NM_027838	3.2	6.2	5	2842	no ARE	stable
Map3k7	26409	mitogen activated protein kinase kinase kinase 7	NM_172688	3.2	8.2	7	3786	Cluster 5	stable
Bax	12028	Bcl2-associated X protein	NM_007527	3.2	0	0	167	no ARE	stable
Mrpl53	68499	mitochondrial ribosomal protein L53	NM_026744	3.2	1.3	1	259	no ARE	stable
Rchy1	68098	ring finger and CHY zinc finger domain containing 1	NM_026557	3.2	0	0	894	no ARE	stable
1810030O07Rik	69155	predicted protein, cXorf38 homolog	NM_175141	3.2	3.6	3	1592	no ARE	stable
Figf	14205	c-fos induced growth factor	NM_010216	3.1	1.3	1	490	no ARE	stable
Ccl25	20300	chemokine (C-C motif) ligand 25	NM_009138	3.1	0	0	520	no ARE	stable
Atf2	11909	activating transcription factor 2	NM_009715	3.1	6.2	5	2413	Cluster 5	stable
Pcgf5	76073	polycomb group ring finger 5	NM_029508	3.1	1.3	1	529	Cluster 5	stable
Tcf12	21406	transcription factor 12	NM_011544	3.1	5.2	4	2320	Cluster 5	stable
Klf3	16599	Kruppel-like factor 3 (basic)	NM_008453	3.1	2.6	2	914	no ARE	1.4(a)
Dpy30	66310	dpy-30 homolog (C. elegans)	NM_024428	3.1	3.6	3	239	no ARE	stable
Suv420h1	225888	suppressor of variegation 4-20 homolog 1 (Drosophila)	NM_144871	3.1	7.5	6	2714	no ARE	2.0(a)
Arf4	11843	ADP-ribosylation factor 4	NM_007479	3.1	4.9	4	1276	no ARE	stable
Isca1	69046	iron-sulfur cluster assembly 1 homolog (S. cerevisiae)	NM_026921	3.1	1.3	1	1440	no ARE	stable
Katna1	23924	katanin p60 (ATPase-containing) subunit A1	NM_011835	3.1	0	0	162	no ARE	stable
Skp1a	21402	S-phase kinase-associated protein 1A	NM_011543	3.1	2.6	2	838	no ARE	stable
Etnk1	75320	ethanolamine kinase 1	NM_029250	3.1	28.8	21	5047	N/A	stable
Cdkn2aipnl	52626	CDKN2A interacting protein N-terminal like	NM_029976	3.1	5.65	4	1441	Cluster 3	stable
Tmpo	21917	thymopoietin	NM_001080129	3.1	13.1	10	1946	N/A	stable
Gnpda2	67980	glucosamine-6-phosphate deaminase 2	NM_001038015	3.1	5.2	4	887	Cluster 5	stable
Rnf138	56515	ring finger protein 138	NM_019706	3.1	9.85	7	2069	no ARE	stable
2310009B15Rik	69549	predicted protein, c1orf53 homolog	NM_001081226	3.1	0	0	81	N/A	stable
Atf7ip	54343	activating transcription factor 7 interacting protein	NM_019426	3.1	2.3	2	483	no ARE	2.1(a)
Lta4h	16993	leukotriene A4 hydrolase	NM_008517	3.1	0	0	106	no ARE	stable
Cops2	12848	COP9 homolog, subunit 2 (Arabidopsis thaliana)	NM_009939	3.1	11.1	9	1907	Cluster 5	stable
Spast	50850	spastin	NM_016962	3.1	4.9	4	2733	no ARE	stable
Capza2	12343	capping protein (actin filament) muscle Z-line, alpha 2	NM_007604	3.1	3.6	3	1130	no ARE	stable
2510009E07Rik	72190	validated uncharacterized protein, c3orf70 homolog	NM_001001881	3.1	6.2	5	4114	no ARE	stable
Ikbkg	16151	inhibitor of kappaB kinase gamma	NM_010547	3.1	20.9	14	5419	no ARE	stable
Manba	110173	mannosidase, beta A, lysosomal	NM_027288	3.1	1	1	958	no ARE	stable
Kif11	16551	kinesin family member 11	NM_010615	3.1	2.3	2	1490	no ARE	stable
Nudt4	71207	nudix (nucleoside diphosph. linked moiety X)-type motif 4	NM_027722	3.1	1	1	978	no ARE	2.8(a)
Mpp5	56217	membrane prot., palmitoylated 5 (MAGUK p55 member 5)	NM_019579	3.1	9.55	7	3094	no ARE	2.7(a)
Ube2l3	22195	ubiquitin-conjugating enzyme E2L 3	NM_009456	3.1	3.6	3	2113	no ARE	stable
Msl1	74026	male-specific lethal 1 homolog (Drosophila)	NM_028722	3.1	2.3	2	2398	no ARE	stable
Gxylt2	232313	glucoside xylosyltransferase 2	NM_198612	3.1	1	1	356	no ARE	unknown
Egln1	112405	EGL nine homolog 1 (C. elegans)	NM_053207	3.1	3.9	3	2121	no ARE	stable
Acpl2	235534	acid phosphatase-like 2	NM_153420	3.1	2.3	2	1342	no ARE	stable
Aldh1a1	11668	aldehyde dehydrogenase family 1, subfamily A1	NM_013467	3.1	0	0	496	no ARE	stable
Bag4	67384	BCL2-associated athanogene 4	NM_026121	3.1	16.6	13	3386	no ARE	3.0(a)
Nln	75805	neurolysin (metallopeptidase M3 family)	NM_029447	3.1	2.6	2	1507	Cluster 5	stable
Rab7	19349	RAB7, member RAS oncogene family	XM_006505762	3.1	2.6	2	1271	N/A	stable
Jak2	16452	Janus kinase 2	NM_008413	3.1	2.6	2	1232	no ARE	stable
Camk2d	108058	calcium/calmodulin-dependent protein kinase II, delta	NM_023813	3.1	9.3	7	2293	Cluster 5	stable
Ski	20481	Sloan-Kettering viral oncogene homolog	NM_011385	3.1	12.9	10	3241	no ARE	stable
Armc2	213402	armadillo repeat containing 2	NM_001034858	3.1	9.3	7	2319	no ARE	stable
Fam107b	66540	family with sequence similarity 107, member B	NM_025626	3.1	5.2	4	2517	no ARE	stable
Tmem41b	233724	transmembrane protein 41B	NM_153525	3.1	6.1	5	2595	no ARE	stable
Tmem218	66279	transmembrane protein 218	NM_025464	3.1	0	0	635	no ARE	stable
Grasp	56149	general rec. for phosphoinositides 1-assoc. scaffold prot.	NM_019518	3.1	6.9	3	751	Cluster 2	stable
Pts	19286	6-pyruvoyl-tetrahydropterin synthase	NM_011220	3.0	5.3	4	611	no ARE	0.4(b),stable
Socs4	67296	suppressor of cytokine signaling 4	NM_080843	3.0	6.5	5	671	no ARE	0.9(b),1.3(c)
1110001J03Rik	66117	predicted protein, c7orf55 homolog	NM_025363	3.0	0	0	87	no ARE	stable
Elavl1	15568	ELAV (embryonic lethal, abnormal vision)-like 1 (HuR)	NM_010485	3.0	30.7	18	4818	no ARE	stable
Ccdc56	52469	coiled-coil domain containing 56	NM_026618	3.0	0	0	370	no ARE	stable
Ufd1l	22230	ubiquitin fusion degradation 1 like	NM_011672	3.0	7.2	6	951	Cluster 5	stable
Ppp1cc	19047	protein phosphatase 1, catalytic subunit, gamma isoform	NM_013636	3.0	2.6	2	1125	no ARE	stable
Timm8b	30057	translocase of inner mitoch. membrane 8 homol. b (yeast)	NM_013897	3.0	1.3	1	220	no ARE	stable
Adnp2	240442	ADNP homeobox 2	XM_006526466	3.0	2.3	2	1346	N/A	1.7(a),1.4(c)
Tspan5	56224	tetraspanin 5	NM_019571	3.0	1	1	1912	no ARE	stable
1110059E24Rik	66206	predicted protein, c9orf85 homolog	NM_025423	3.0	1.3	1	877	no ARE	stable
Rbmx	19655	RNA binding motif protein, X chromosome	NM_011252	3.0	2	2	1053	no ARE	stable
Serp1	28146	stress-associated endoplasmic reticulum protein 1	NM_030685	3.0	6.5	5	1865	no ARE	stable
Hbxip	68576	hepatitis B virus x interacting protein	NM_026774	3.0	1	1	345	N/A	stable
Uchl3	50933	ubiquitin carboxyl-terminal esterase L3	NM_016723	3.0	0	0	157	no ARE	stable
Nufip2	68564	nucl. fragile X mental retardation prot. interacting protein 2	NM_001024205	3.0	34.65	26	8159	no ARE	stable
Ugcg	22234	UDP-glucose ceramide glucosyltransferase	NM_011673	3.0	7.8	6	2440	Cluster 5	stable
Bpnt1	23827	bisphosphate 3'-nucleotidase 1	NM_011794	3.0	7.6	6	1132	Cluster 5	stable
Arpc3	56378	actin related protein 2/3 complex, subunit 3	NM_019824	3.0	0	0	278	no ARE	stable
Timm17a	21854	translocase of inner mitochondrial membrane 17a	NM_011590	3.0	5.2	4	383	no ARE	stable
Seh1l	72124	SEH1-like (S. cerevisiae	NM_001039088	3.0	5.2	4	2325	no ARE	stable
Nagk	56174	N-acetylglucosamine kinase	NM_019542	3.0	0	0	101	no ARE	stable
