# Supplementary material for: Short-lived AUF1 p42-binding mRNAs of RANKL and BCL6 have two distinct instability elements each
Source: PLoS One. 2018 Nov 12;13(11):e0206823. doi: 10.1371/journal.pone.0206823 (PMC6231638; doi:10.1371/journal.pone.0206823)
Supplement: S8 Table — The test sequences correspond to the instability elements (pink sequences) determined for mouse Rankl mRNA and human BCL6 mRNA in this study, and for human IL6 mRNA in a previous study (Paschoud et al., 2006)[4]. They include adjacent AUF1-binding regions. In additon a mouse Smad6 sequence with a predicted good alignment with the mouse Rankl A test sequence was used as a test sequence. Each test sequence was aligned with 3'UTRs of mRNAs listed in S9 Table. (PDF) [file pone.0206823.s011.pdf]

S8 Table. Test sequences used for optimal local sequence alignments between instability elements and selected 3'UTRs. The test sequences correspond to the instability elements (pink sequences) determined for mouse Rankl mRNA and human BCL6 mRNA in this study, and for human IL6 mRNA in a previous study (Paschoud et al., 2006)[4]. They include adjacent AUF1-binding regions. In additon a mouse Smad6 sequence with a predicted good alignment with the mouse Rankl A test sequence was used as a test sequence. Each test sequence was aligned with 3'UTRs of mRNAs listed in S9 Table.

Test sequence	Quality	Sequence
mouse Rankl A	decay element 1	TTTTTATATAATGTCTAAAGTTATATTTCAGG
mouse Rankl B	decay element 2; AUF1-binding region	TTATATTTGTGCTATAGTATTTGATTCAAAATATTTAAAAATGTCTCACTGTTGACATATT
mouse Rankl C	decay element 2; same extended 5'	TGCAAAGTTTTGTAAATTATATTTGTGCTATAGTATTTGATTCAAAATATTTAAAAAT
human BCL6 A	decay element 1; AUF1 binding region long	AGACTTCAGTATGTTGTCAAAGAGAGGGCTTTAATTTTTTTAACCAAAGGTGAAGGAATATATGG CAGAGTTGTAAATATATAAATATATATATATATAAAATAAATATATATAAACCTAA
human BCL6 B	decay element 2; potential hairpin	TTGTATCTGCAGGCAGACACGGATCTGAGA
human BCL6 C	decay element 1; AUF1 binding region short	AATTTTTTTAACCAAAGGTGAAGGAATATATGGCAGAGTTGTAAATATATAAATATATATATATA TAAAATAAATATATATAAACCTAA
human IL6 A	decay element 1; potential hairpin	AACTTATGTTGTTCTCTATGGAGAACTAAAAGTATGAGCGTTAGG
human IL6 B	decay element 2; AUF1 binding region long	TTTTAATTATTTTTAATTTATTAATATTTAAATATGTGAAGCTGAGTTAATTTATGTAAGTCATA TTTATATTTTT
human IL6 C	decay element 1; minimal region for hairpin	AACTTATGTTGTTCTCTATGGAGAACTAAAAGTAT
human IL6 D	decay element 2; AUF1 binding region short	ATTTAAATATGTGAAGCTGAGTTAATTTATGTAAGTCATATTTATATTTTT
mouse Smad6 A	predicted similarity with Rankl A test-sequence	ATTTAATATAAAGTTTTATATATTATATGGAAATATATATTATA
