# Supplementary material for: Short-lived AUF1 p42-binding mRNAs of RANKL and BCL6 have two distinct instability elements each
Source: PLoS One. 2018 Nov 12;13(11):e0206823. doi: 10.1371/journal.pone.0206823 (PMC6231638; doi:10.1371/journal.pone.0206823)
Supplement: S9 Table — These target sequences were aligned with the test sequences of S8 Table. They correspond to 3'UTRs of the most unstable mRNAs of this study (S7 Table), to 3'UTRs of mRNAs previously recognized as unstable, to known binding targets of AUF1, or to potential targets of Zc3h12a. mRNA half-lives are either known prior to this study in mouse ES cells (a) (Sharova et al., 2009)[37], or for human homologs in lymphocytes (b) (Raghavan et al., 2002)[35] and HepG2 cells (c) (Yang et al., 2003)[36], or known from other studies (d) (Paschoud et al., 2006)[4], (e) (Shaw and Kamen, 1986)[26], (f) (Li et al., 2012)[56], (h) (Gorospe et al., 1993), or were determined in this study (g). For Cdkn2a, Rel, and Zfp266 the alignment between mouse and human UTRs were very poor. (PDF) [file pone.0206823.s012.pdf]

S9 Table. Target 3'UTR sequences used in optimal local sequence alignments. These target sequences were aligned with the test sequences of S8 Table. They correspond to 3'UTRs of the most unstable mRNAs of this study (S7 Table), to 3'UTRs of mRNAs previously recognized as unstable, to known binding targets of AUF1, or to potential targets of Zc3h12a. mRNA half-lives are either known prior to this study in mouse ES cells (a) (Sharova et al., 2009)[37], or for human homologs in lymphocytes (b) (Raghavan et al., 2002)[35] and HepG2 cells (c) (Yang et al., 2003)[36], or known from other studies (d) (Paschoud et al., 2006)[4], (e) (Shaw and Kamen, 1986)[26], (f) (Li et al., 2012)[56], (h) Gorospe et al., 1993), or were determined in this study (g). For Cdkn2a, Rel, and Zfp266 the alignment between mouse and human UTRs were very poor.

Gene	References for AUF1-binding or AREs	Particular features	Half-life
Tnfsf11=Rankl	This study		1.5(a),0.5(g)
Bcl6	This study		1.3(a),1.4(c),0.6(g)
IL6	Paschoud et al., 2006; Matsushita et al., 2009	putative target of Zc3h12a Rnase	0.5(d)
Smad6	This study		1.9(a),0.3(g)
Acvr1	This study		2.5(a),1.1(b)
Adnp2	This study		1.7(a),1.4(c)
Arl4a	This study		2.2(a),0.8(c)
Bcl2	Lapucci et al., 2002	reported AUF1 target	6.0(a)
Bmp4	This study		2.5(a),0.9(c)
c8orf4	This study		1.1(a)
Ccnd1	Lin et al., 2000; Lal et al., 2004	reported AUF1 target	8.6(a)
Ccnt2	This study		0.7(b),1.3(c),0.9(g)
Cdkn2a=p16	Wang et al., 2005	reported AUF1 target	14.0(a)
Csf2=GMCSF	Shaw and Kamen, 1986	typical ARE	0.5(e)
Fos	Treisman, 1985	typical ARE	1.3(a)
Foxj3	This study		1.4(a),1.3(c)
Fzd4	This study		0.9(g)
Gadd45a	Lal et al., 2006		2.5(a)
Hes1	This study		1.2(a),0.3(g)
Hivep2	This study		2.0(a)
Ierl5l	This study		1.4(a)
IL12b	Matsushita et al., 2009	putative target of Zc3h12a Rnase	12.0(a)
IL1b	Mizgalska et al., 2009	putative target of Zc3h12a Rnase	0.5(h)
IL2	Li et al., 2012	putative target of Zc3h12a Rnase	0.8(f)
Klf3	This study		1.4(a)
Klf10	This study		2.0(a),0.8(g)
Maf	This study		1.5(g)
Mllt11	This study		2.0(a)
Myc	Brewer, 1991		1.0(a)
Otud1	This study		1.3(a)
Phf13	This study		2.0(a)
Ptgs2=Cox2	This study; Cok et al., 2004	reported AUF1 target	6.1(a)
Rel	Uehata et al., 2013	putative target of Zc3h12a Rnase	5.2(a)
Rnd3	This study		2.1(a)
Rnf113a2	This study		2.4(a),1.5(b)
Sin3a	This study		1.5(g)
Slc30a1	This study		2.5(a),1.6(c)
Socs4	This study		0.9(b),1.3(c)
Suv420h1	This study		2.0(a)
Tiparp	This study		1.1(a)
Tnf	Wilson et al., 2001	typical ARE; reported AUF1 target	1.2(a)
Tnfaip6	This study		1.8(a),2.0(g)
Tspyl3	This study		2.0(a)
Zfp248	This study		1.6(a)
Zfp266	This study		2.0(a)
