# Supplementary material for: Short-lived AUF1 p42-binding mRNAs of RANKL and BCL6 have two distinct instability elements each
Source: PLoS One. 2018 Nov 12;13(11):e0206823. doi: 10.1371/journal.pone.0206823 (PMC6231638; doi:10.1371/journal.pone.0206823)
Supplement: S10 Table — 3'UTRs of mouse mRNAs listed in S9 Table were aligned with specific instability elements and AUF1-binding regions described in detail in S8 Table. Most alignments are in regions with conservation between mouse and human mRNAs. (PDF) [file pone.0206823.s013.pdf]

S10 Table. Significant local sequence alignments in 3'UTRs. 3'UTRs of mouse mRNAs listed in S9 Table were aligned with specific instability elements and AUF1-binding regions described in detail in S8 Table. Most alignments are in regions with conservation between mouse and human mRNAs.

Test sequence	Quality	Rankl	Bcl6	IL6	Smad6	Acvr1	Adnp2	Arl4a	Bcl2	Bmp4	c8orf4	Ccnd1	Ccnt2	Cdkn2a	Csf2	Fos	Foxj3	Fzd4	Gadd45a	Hes1	Hivep2	Ierl5l	IL12B	IL1B	IL2	Klf3	Klf10	Maf	Mllt11	Myc	Otud1	Phf13	Ptgs2	Rel	Rnd3	Rnf113a2	Sin3a	Slc30a1	Socs4	Suv420h1	Tiparp	Tnf	Tnfaip6	Tspyl3	Zfp248	Zfp266
mouse Rankl A	decay element 1	grey			yellow										yellow				yellow																											
mouse Rankl B	decay element 2; AUF1-binding region	grey					yellow				orange							yellow	orange		orange					yellow				orange																
mouse Rankl C	decay element 2; extended 5'	grey																	red	yellow	orange				yellow						yellow															
human BCL6 A	decay element 1; AUF1 binding region long		grey							yellow				orange																										yellow			yellow			
human BCL6 B	decay element 2; potential hairpin		grey															yellow																								yellow				
human BCL6 C	decay element 1; AUF1 binding region short		grey							yellow				orange																										yellow						
human IL6 A	decay element 1; potential hairpin			grey																	orange																			yellow						
human IL6 B	decay element 2; AUF1 binding region long			grey							yellow				orange	orange			yellow						orange			yellow			yellow		orange							yellow		red				
human IL6 C	decay element 1; minimal region for hairpin			grey																	orange																		yellow	orange						
human IL6 D	decay element 2; AUF1 binding region short			grey											orange	yellow							yellow	orange	red	yellow			yellow				red									yellow				
mouse Smad6 A	predicted similarity with Rankl A test sequence				grey																				yellow														yellow					yellow		

Colorcode for grading of significance (p-values)

0.05 - >0.01	yellow
0.01 - >0.001	orange
0.001 and lower	red
